# GPCR-MAPS: high-resolution functional and allosteric mapping of G-protein-coupled receptor activation functions and bias

**DOI:** 10.1038/s41594-026-01907-3

**Published:** 2026-11-11

**Authors:** Taylor L. Mighell, Ben Lehner

**Affiliations:** 1 Centre for Genomic Regulation (CRG), The Barcelona Institute of Science and Technology, Barcelona, Spain; 2 Wellcome Sanger Institute, Wellcome Genome Campus, Hinxton, UK; 3 Universitat Pompeu Fabra (UPF), Barcelona, Spain; 4 Institució Catalana de Recerca i Estudis Avançats (ICREA), Barcelona, Spain

**Keywords:** Molecular biology, Systems biology, Genomics, Drug discovery

## Abstract

G-protein-coupled receptors (GPCRs) are important drug targets. Approaches targeting allosteric modulation and biased signaling, as opposed to the orthosteric pocket, can improve safety profiles of therapeutics. However, rational design of precision therapies is limited by the lack of high-resolution quantitative functional annotation of GPCR structures. Here we present GPCR-MAPS—a fast and general approach to build complete, high-resolution, multimodal functional and allosteric maps of GPCRs. GCPR-MAPS quantifies direct recruitment of proteins and deconvolves the substantial effects of amino acid substitutions on receptor expression. We applied GPCR-MAPS to the β_2_ adrenergic receptor to generate >7,500 dose–response curves and reveal a modular receptor architecture, with a core activation network surrounded by residues controlling quantitative parameters and bias. We used this information to identify multiple allosteric surface pockets. The application of this approach across the superfamily of GPCRs could unlock deep understanding of these receptors and enable rational design of precision GPCR therapeutics.

## Abstract (web summary)

Mighell and Lehner present GPCR-MAPS, which enables scalable functional mapping of G-protein-coupled receptors. They obtained measurements across multiple phenotypes of the β_2_ adrenergic receptor to reveal a modular activation architecture and determinants of signaling bias, prioritizing undrugged allosteric sites.

## Main

G-protein-coupled receptors (GPCRs) are the largest family of transmembrane (TM) proteins that transmit signals from outside to inside cells. These signals are diverse and include neurotransmitters, metabolites, immune system molecules, pH and photons of light^1^. GPCRs have seven TM helices connected by alternating intracellular and extracellular loops (ECLs and ICLs). Binding of an agonist to the extracellular portion of the receptor results in an allosteric conformational change^2^ enabling binding and activation of one or more heterotrimeric G proteins, which subsequently activate diverse downstream signaling pathways^3^. Activation also results in phosphorylation of the receptor, including in the C-terminal tail, and recruitment of β-arrestin, which terminates G-protein signaling by internalizing the receptor and also scaffolds G-protein-independent signaling^4^. Because of their role as master signal transducers and their availability on the cell surface, GPCRs are the most important class of drug targets, with one third of current clinical therapeutics targeting GPCRs^5,6^.

The vast majority of GPCR drugs bind the orthosteric site^7^. However, alternative modalities exist, including biased agonism^8^ (also called ‘functional selectivity’) and allosteric modulation^9^. Biased agonists preferentially signal through one downstream pathway (for example, G protein over β-arrestin), which can improve therapeutic efficacy. A therapeutic benefit for biased agonism has been proposed for at least 30 GPCRs^10^.

Allosteric drugs bind receptors outside the orthosteric site. Structural conservation of orthosteric sites makes it difficult to target one receptor without engaging other family members, often causing toxicity and other side-effects that result in clinical failure^7^. In contrast, allosteric drugs targeting nonconserved sites can have high specificity and reduced toxicity. Furthermore, as allosteric drugs modulate responses to physiological ligands, their effects temporally and spatially match physiological signaling processes^11^. Because of these major advantages, increasing effort is being put toward the development of biased and allosteric modulators^12^. However, with our current understanding of GPCR function, these molecules are typically only found by blind screening or iteration upon previously discovered molecules^13,14^.

To design better GPCR drugs, an improved understanding of the functional and allosteric architecture linking agonist binding with intracellular signaling is required. Structural studies have identified conserved ‘microswitches’, including the CWxP, DRY, PIF and NPxxY motifs, where local structural rearrangements occur upon activation^15,16^.

A powerful complementary approach for understanding proteins is mutagenesis followed by functional characterization^17^. The effects of hundreds of alanine substitutions on GPCR signaling through one^18^ or two^19,20^ pathways have revealed a diversity of signaling responses. Pooled approaches have characterized surface expression^21–25^, ligand binding^23,26^ and single or few concentration transcriptional responses downstream of GPCRs^27–30^. However, the limited throughput^18–20^, focus on only one of 19 variants^18–20^ and lack of quantification of multimodal responses and signaling parameters limit the insights that can be obtained^21–30^.

With respect to drug discovery, a key challenge is to understand how ligands modulate receptor signaling. Are all ligand-contacting sites equally important for a response or just a subset of ‘hotspot’ residues^31^?

A further challenge is to understand how perturbations across a receptor’s structure alter bias in the recruitment of different transducer proteins^32^ and to identify allosteric sites where perturbations can inhibit, activate or modulate signaling. GPCRs have many small pockets to potentially therapeutically target^33^ and it would be extremely useful to know at which of these sites perturbations alter receptor responses and how.

Here, we present a general high-throughput method, GPCR-MAPS (GPCR multiplexed assays with pathway specificity), to rapidly generate comprehensive functional maps of GPCRs. We apply GPCR-MAPS to the β_2_ adrenergic receptor (β_2_AR), one of the most extensively characterized receptors. β_2_AR is expressed in the cardiovascular system where it modulates smooth muscle relaxation and bronchodilation; many β_2_AR agonists have been developed and are used for treating asthma and chronic obstructive pulmonary disease^34^.

GPCR-MAPS has four components. First, we generate a complete site-saturation library of a receptor, consisting of all possible amino acid substitutions in all positions. Second, we quantify the cell surface expression of the library of variants to quantify defects in fold stability and trafficking. Third, we use pooled experiments to quantify the recruitment of conformation-specific interaction partners to every receptor variant in response to ligand treatment. Using ligand titrations allows the complete concentration–response activation functions of all possible receptor variants to be quantified in a single experiment. Measuring the recruitment of different interaction partners quantifies changes in receptor bias. Fourth, we integrate the resulting data to generate rich multimodal functional maps of how the quantitative and multimodal functions of a receptor are altered by chemically diverse perturbations throughout its entire structure.

Applied to β_2_AR, GPCR-MAPS quantifies the activation functions and multimodal characteristics of >7,500 different receptor variants. The resulting maps reveal a modular receptor architecture, with a core activation network surrounded by residues tuning quantitative parameters and bias. The maps also identify multiple allosteric surface pockets. We believe that the application of GPCR-MAPS across the GPCR superfamily will advance our understanding of the largest family of human membrane receptors and enable rational, precision drug design.

## Results

### GPCR-MAPS

To allow quantification of multimodal receptor phenotypes for thousands of receptor variants in parallel, including full concentration–response curves, we developed a modular platform that we call GPCR-MAPS. Our goal is to read out receptor states as directly as possible. Therefore, in contrast to other approaches^27–30^, we focused on quantifying direct binding interactions with receptors. Quantifying direct binding has proven powerful for understanding signaling by soluble proteins^35^ and GPCRs^36^ and avoids the confounding influence of nonlinearities, feedback and differential signal amplification when quantifying activity downstream of endogenous signaling networks.

GPCR-MAPS is compatible with cassette-based^37,38^ or SUNi mutagenesis^39^ to generate a pooled library of receptor variants containing all possible single amino acid substitutions. The library is barcoded and rapidly integrated into a single locus in engineered HEK293 ‘landing pad’ cells^40^ such that each cell expresses a single copy of a single variant.

**Fig. 1:**
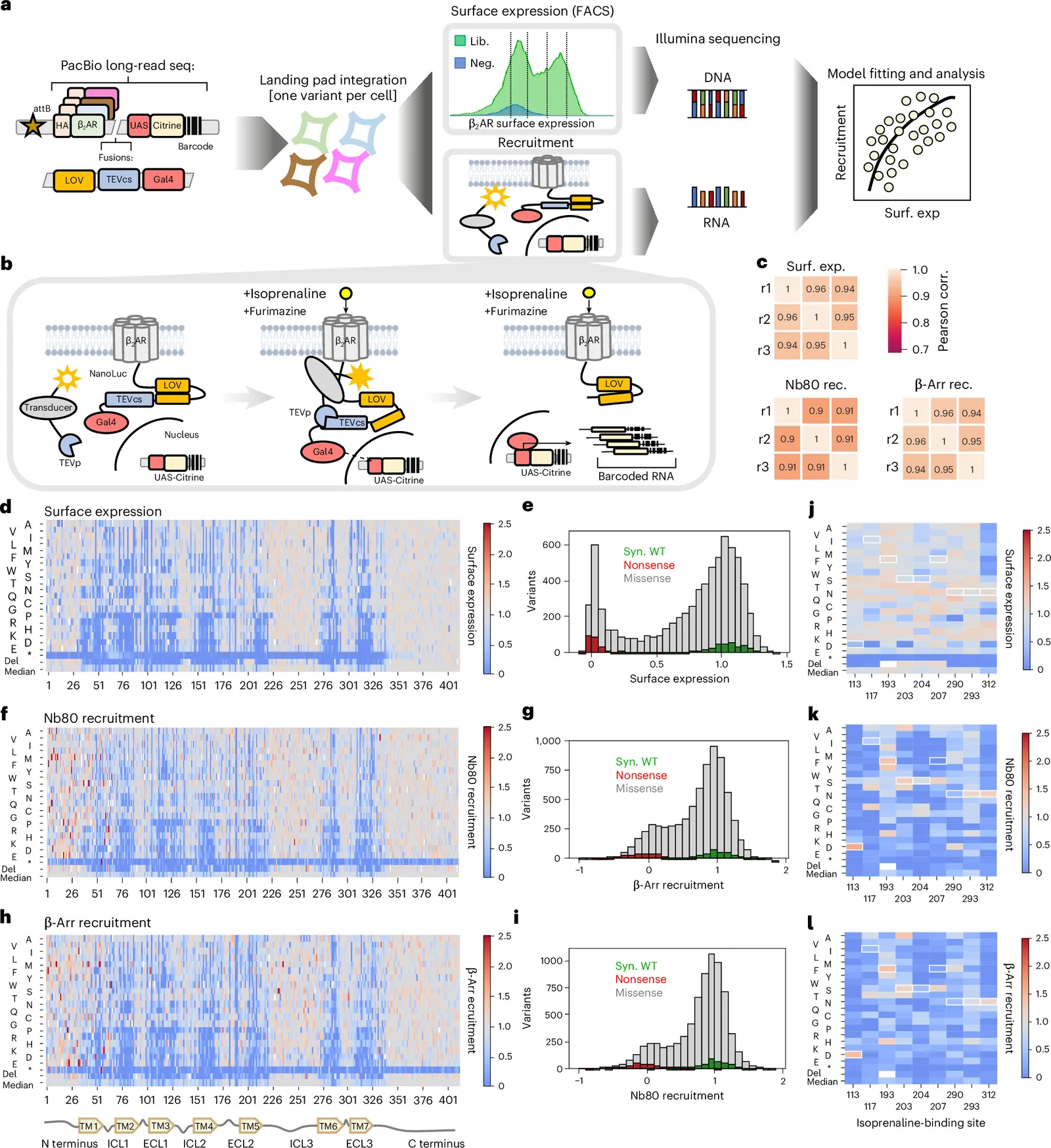
GPCR-MAPs. **a**, Schematic of the experimental framework. A mutagenized GPCR (in this case β_2_AR) is encoded in a plasmid that also includes the attB recombination site, an N-terminal HA epitope tag, C-terminal fusions of an LOV domain, the TEVcs and the Gal4 transcription factor. Downstream, on the same plasmid, is the fluorescent protein Citrine, driven by a Gal4-responsive UAS promoter, with a degenerate DNA barcode included in the 3′ UTR. This construct is recombined into HEK 293T LLP-iCasp9-Blast landing pad cells. Then, two assays are performed to measure the surface expression (FACS-based) and recruitment activity (transcriptional reporter) of variants en masse. The outputs of these experiments (DNA for FACS and RNA for transcriptional reporter) are sequenced with short-read technology. Estimated surface expression and recruitment scores are then modeled to understand the contribution of each of these to the variant effects. **b**, Detailed schematic of the recruitment assay. The receptor construct is expressed from landing pad cells while the transducer construct is transiently transfected. Addition of agonist (isoprenaline in this case) and furimazine results in recruitment of transducer to the receptor fusion and subsequent cleavage of the TEVcs, liberating Gal4, which moves to the nucleus and drives transcription of the UAS–Citrine–barcode construct. **c**, Replicate Pearson correlations for the surface expression (surf. exp.), Nb80 recruitment (rec.) and β-arrestin recruitment (*P* < 10^−100^ in each case). **d**, Heat map representation of the surface expression data. β_2_AR positions are shown on the *x* axis; substitutions are shown on the *y* axis. Gray cells indicate expression similar to the wild type while blue and red cells indicate reduced and increased expression, respectively. The asterisk indicates the stop codon. **e**, Histogram of surface expression variant scores; nonsense variants are shown in red, synonymous wild-type variants (Syn. WT) are shown in green and missense variants are shown in gray. **f**, Heat map representation of the Nb80 recruitment data. The axes and color scale are as in **d**. **g**, Histogram of Nb80 recruitment scores. Colors are as in **e**. **h**, Heat map representation of β-arrestin (β-Arr) recruitment scores. The axes and color scale are as in **d**. **i**, Histogram of β-arrestin recruitment scores. Colors are as in **e**. **j**, Surface expression scores for residues in the isoprenaline-binding site in the solved structure (PDB 7DHR). **k**, Nb80 recruitment scores for residues in the isoprenaline-binding site. **l**, β-Arrestin recruitment scores for residues in the isoprenaline-binding site. del, deletion. Source data

**Extended Data Fig. 1:**
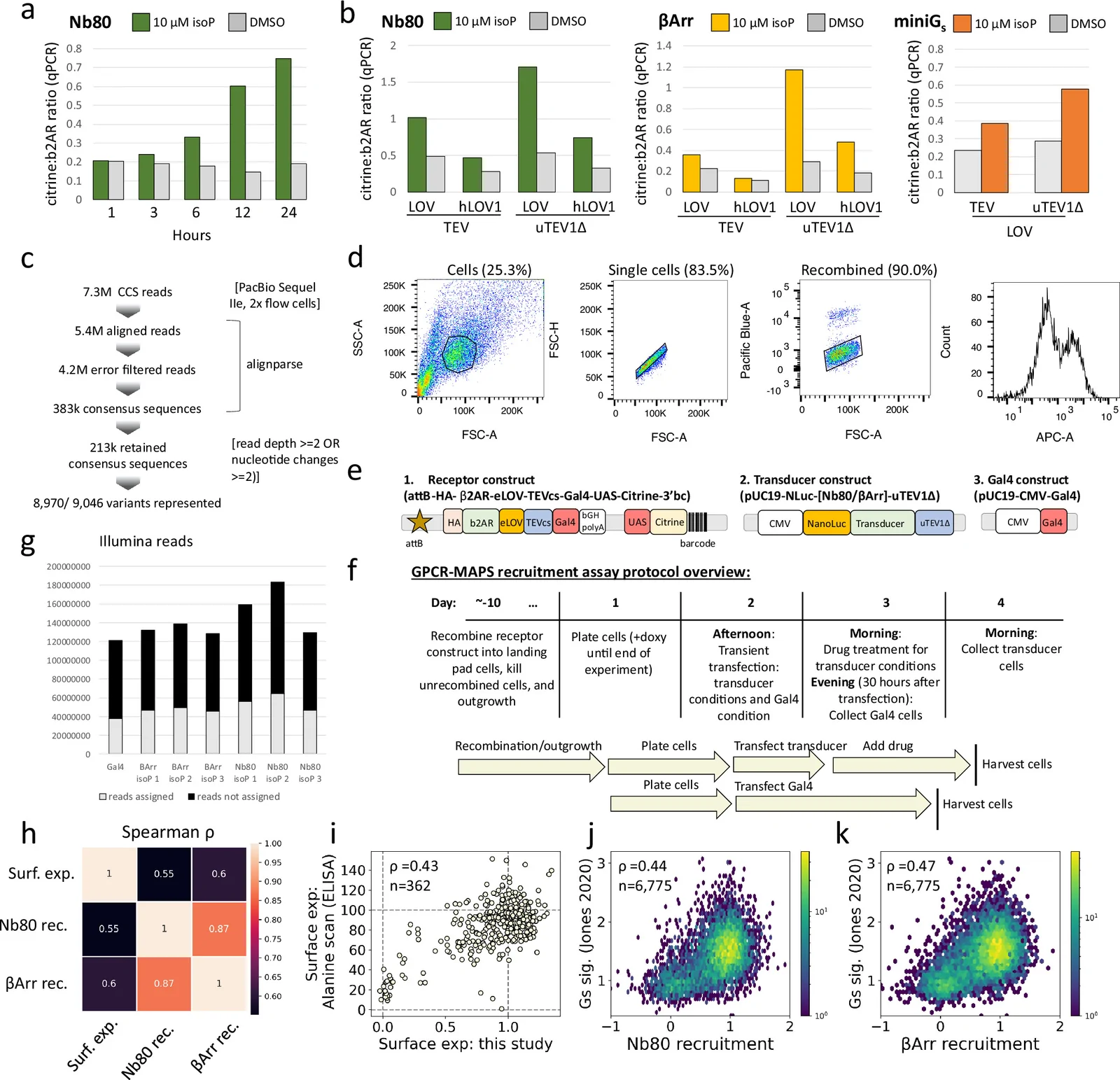
GPCR-MAPS development. **a** GPCR-MAPS timecourse experiment. Cells expressing the β2AR receptor construct in the landing pad were transiently transfected with transducer construct bearing Nb80 and the next day treated with 10 μΜ isoprenaline. Drug-treated cells were then incubated for varying amounts of time before cells were harvested and qPCR was used to measure reporter abundance. The qPCR was performed with a set of primers amplifying the Citrine reporter construct and a set of primers amplifying the β2AR coding sequence as a reference. Recruitment was then inferred by the ratio of Citrine to β2AR. **b** GPCR-MAPS experiment, performed with 24 h drug incubation, but with different genotypes for the TEV protease and the LOV domain. Expriments were done as in (a) but with Nb80, β-Arrestin, or miniG_s_ as the transducer. **c** Processing steps and numbers of reads/sequences at each step for the processing and analysis of PacBio long read sequencing linking variants to barcodes. **d** Gating strategy for FACS. **e** Schematics of the constructs used in GPCR-MAPS. The receptor construct encodes the HA-tagged receptor, fused to eLOV, TEVcs, and Gal4; downstream is Citrine driven by the UAS promoter with the barcode in the 3′ UTR. This construct is integrated into the landing pad. The transducer construct contains a fusion of NanoLuciferase with the transducer and TEV protease, driven by CMV promoter. This construct is transiently transfected. The final construct is Gal4 alone, driven by CMV promoter. This construct is used to stimulate maximal reporter levels and serves to normalize differences in barcode abundance across the library as well as any barcode-specific effects on RNA stability in cells. **f** Timeline schematic of the GPCR-MAPS recruitment experiment, and the operations that are performed each day. **g** The number of Illumina reads acquired for each sample, with black and gray portions of bars indicating the fraction of reads that were assigned or not to barcodes. **h** Spearman correlations between surface expression, Nb80 recruitment, and β-Arrestin recruitment. **i** Surface expression measurements from this study compared to those generated in Heydenreich et al. ^18^. **j** Nb80 recruitment versus G_s_ signaling from Jones et al. ^27^. **k** β-arrestin recruitment versus G_s_ signaling from Jones et al. ^27^. Source data

The cell surface expression of each variant is measured using fluorescence-activated cell sorting (FACS) followed by sequencing^21,22,25^ (Fig. 1a). To quantify recruitment of receptor interaction partners in response to ligand treatment, we developed a modular protein interaction assay based on a previously described platform^41^ but extensively reengineered to enable massively multiplexed experiments. We improved the signal-to-noise ratio of the platform and extended it to allow modular readout of the recruitment of diverse GPCR protein interaction partners including the active-state-specific nanobody Nb80 (ref. ^42^), β-arrestin and mini-G_s_^43^ (Extended Data Fig. 1a,b).

The platform uses three genetic constructs: (1) a GPCR of interest with a fusion of the exogenous transcription factor Gal4, linked by a sequence encoding an evolved light, oxygen and voltage sensitive (eLOV) domain and a tobacco etch virus protease cleavage site (TEVcs); (2) a GPCR binding partner (for example, β-arrestin) fused to NanoLuciferase (NLuc) and TEV; and (3) the fluorescent protein Citrine under the control of the UAS promoter (Fig. 1b and Extended Data Fig. 1e). Agonist-mediated activation of the GPCR causes recruitment of the binding partner to the receptor. In the absence of light, the eLOV domain cages the TEVcs and prevents cleavage; however, coaddition of the NLuc substrate furimazine results in bioluminescence and, when the transducer brings NLuc into proximity with the eLOV domain, the steric hindrance is relieved and TEVp can cleave at TEVcs. Cleavage relies on coincidence of the NLuc:eLOV interaction, as well as interactions between the GPCR and its binding partner, reducing background signal^41^. This cleavage liberates Gal4, which translocates to the nucleus and drives transcription of the UAS–Citrine reporter construct, which is quantified by short-read RNA sequencing (Fig. 1b).

The recruitment platform allows the binding of multiple interaction partners to be quantified in a single condition (for example, ligand concentration). However, because of its highly multiplexed RNA-seq-based readout, it also allows the quantification of protein recruitment across many different conditions (for example, ligand combinations, changes in culture conditions or, as we demonstrate here, ligand titrations), allowing the construction of full concentration–response curves for thousands of different receptor variants in a single experiment.

### Application to β_2_AR

We generated a saturation mutagenesis library of β_2_AR containing all missense and nonsense substitutions, synonymous variants and single-residue deletions (Extended Data Fig. 1e). After introducing degenerate nucleotide barcodes into the 3′ untranslated region (UTR) of Citrine, we used long-read sequencing to make 212,840 β_2_AR genotype–barcode links (Extended Data Fig. 1c). These represented 8,970 of an intended 9,046 unique genotypes (99.2%). We introduced this library into HEK 293T landing pad cells^40^, ensuring that a single variant is expressed per cell.

### β_2_AR surface expression

To quantify surface expression, cells were immunostained with anti-HA antibody and sorted into four bins by fluorescence intensity (Extended Data Fig. 1d); barcodes were quantified using short-read sequencing to yield 8,740 measurements that were highly reproducible across three replicates (Pearson *r* = 0.94–0.96; Fig. 1a,c and Supplementary Table 1). Nonsense substitutions are uniformly damaging, except those occurring within ICL1 and those in the C terminus, suggesting that the receptor can be stably expressed as either a single-pass TM protein or a C-terminal tail truncation. The TM helices are more substitution sensitive than ECLs or ICLs, consistent with previous work^21–25,28^ (Fig. 1d). Synonymous wild-type and nonsense variants have well separated expression distributions, with missense variants spanning the full range between those two classes (Fig. 1e). Our surface expression estimates are in line with previously estimated surface expression levels of alanine substitutions^18^ (Spearman’s *ρ* = 0.43; Extended Data Fig. 1i).

### β-arrestin and Nb80 recruitment

We next quantified recruitment of two conformation-specific binders: Nb80 (as a surrogate for G protein, as Nb80 returns a higher signal-to-noise ratio than mini-G_s_; Extended Data Fig. 1b) and β-arrestin. We transiently transfected one of the binder constructs (pUC19-NLuc-[Nb80/β-arrestin]-uTEV1Δ; Extended Data Fig. 1e) into cells bearing the β_2_AR receptor construct in the landing pad. We also included a condition where Gal4 was transfected alone (pUC19-CMV-Gal4; Extended Data Fig. 1e) to measure relative abundance in the library combined with any barcode-specific effects on RNA stability. The next day, a saturating concentration of isoprenaline (10 μM) was added to the experimental samples to induce activation and cells were collected 24 h later. The Gal4 maximum induction samples were collected 30 h after transfection. RNA was collected from all samples, reverse-transcribed, converted to short-read sequencing libraries and sequenced (Extended Data Fig. 1f,g).

Recruitment scores were calculated using the variant frequency in the experimental samples compared to variant frequency in the Gal4 condition and rescaled, with 0 representing the median of nonsense variants recruitment and 1 representing wild-type recruitment. Low-frequency variants were filtered to yield 7,850 high-confidence Nb80 and β-arrestin recruitment scores (*r* = 0.9–0.91 for Nb80 replicates, *r* = 0.88–0.89 for β-arrestin; Fig. 1c and Supplementary Table 1). TM regions are most sensitive to substitution (Fig. 1f–h). Missense variants again span the whole distribution from nonsense to wild-type-like (Fig. 1g,i). The Nb80 and β-arrestin recruitment scores are also correlated with each other (Spearman’s *ρ* = 0.87), as well as with previous multiplexed measurements of β_2_AR variants signaling through the G_s_ pathway^27^ (Spearman’s *ρ* = 0.44 and 0.47 for Nb80 and β-arrestin recruitment, respectively; Extended Data Fig. 1j,k).

### Deconvolving recruitment and expression

**Fig. 2:**
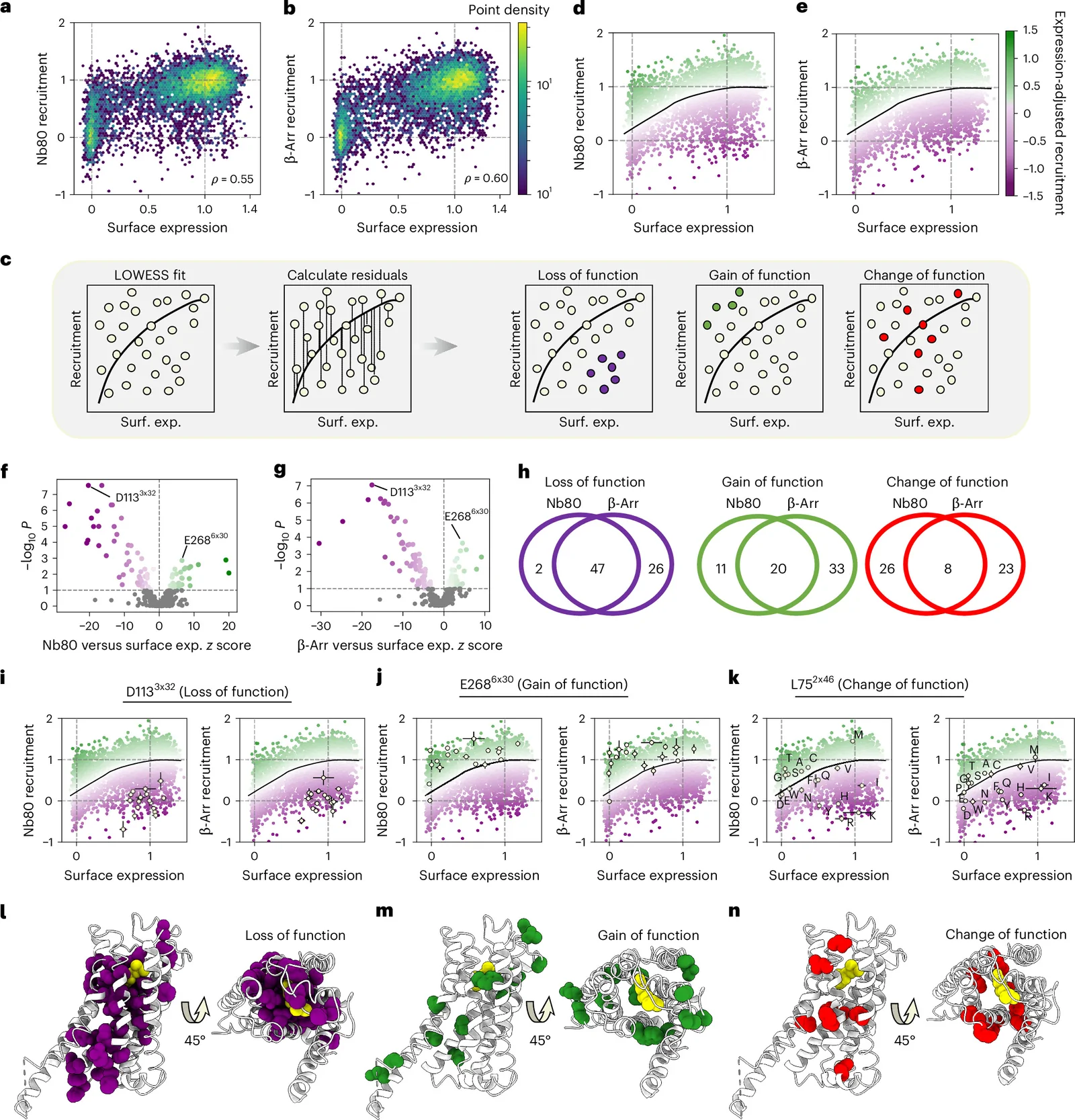
Deconvolving recruitment and expression. **a**, Surface expression versus Nb80 recruitment scores for missense variants (Spearman’s *ρ* = 0.55, *P* < 10^−100^). **b**, Surface expression versus β-arrestin recruitment scores for missense variants (Spearman’s *ρ* = 0.55, *P* < 10^−100^). **c**, Schematic of the method for identifying residues contributing to recruitment beyond what can be explained by surface expression. First, the recruitment data are related to surface expression with a LOWESS regression. Then, for each variant, residuals to this fit are calculated. Then, residues with significantly biased residuals are identified as LoF or GoF using a Mann–Whitney *U*-test. Lastly, positions where variants have a wide range of effects, quantified using the IQR of variant *z* scores, are identified as CoF. **d**, LOWESS fit of surface expression versus Nb80 recruitment, with variant points colored by residual to the fit. **d**, Same as **e**, but for β-arrestin recruitment. **f**, Volcano plot showing the position-average *z* score and the −log_10_-transformed *P* value (Mann–Whitney *U*-test, two-sided) of all residues for Nb80 recruitment. Residues with significantly biased residuals (FDR = 0.1) are colored according to the average *z* score. **g**, Same as in **f**, but for β-arrestin recruitment. **h**, Venn diagrams showing the number of residues called LoF, GoF and CoF for Nb80 and β-arrestin recruitment. **i**, Surface expression and recruitment effects of variants at D113^3×32^, plotted over the population of variant effects, for Nb80 and β-arrestin recruitment versus surface expression. Error bars represent the s.e.m. of three experimental replicates. **j**, Same as **i**, but with variants at position E268^6×30^. **k**, Same as **i**,**j**, but with variants at position L75^2×46^. **l**, LoF residues highlighted in purple on the isoprenaline-bound β_2_AR structure (PDB 7DHR; isoprenaline in yellow). **m**, GoF residues highlighted in green on β_2_AR. **n**, CoF residues highlighted in red on β_2_AR. Source data

Plotting Nb80 and β-arrestin recruitment scores against receptor expression reveals that changes in expression are an important cause of changes in recruitment (*ρ* = 0.55 and 0.6, for Nb80 and β-arrestin, respectively; Fig. 2a,b and Extended Data Fig. 1h). However, some substitutions have much larger effects on recruitment than can be explained by changes in expression. For example, missense substitutions at the nine positions making up the isoprenaline-binding site^44^ have a minimal effect on expression (Fig. 1j), while these same substitutions are strongly deleterious for recruitment of both binding partners (Fig. 1k,l).

### Residues contributing to recruitment beyond expression changes

The relationship between recruitment and expression is nonlinear (Fig. 2a,b). Therefore, we fit a LOWESS (locally weighted scatterplot smoothing) regression to the data, which acts as a null expectation of recruitment produced by a given surface expression level. This null expectation explains a significant amount of variance (*R*^2^ = 0.37 and 0.42 for Nb80 and β-arrestin, respectively).

We quantified changes in recruitment not explained by expression changes by calculating recruitment residuals and *z* scores (that is, residual divided by the s.e.m. for each variant; Fig. 2c–e). Comparing the group of *z* scores at each position to the *z* scores from all other positions with a two-sided Mann–Whitney *U*-test allows us to identify residues where substitutions have greater or lesser recruitment than would be expected on the basis of their surface expression level (Supplementary Table 2).

### Complete functional maps of Nb80 and β-arrestin recruitment

**Extended Data Fig. 2:**
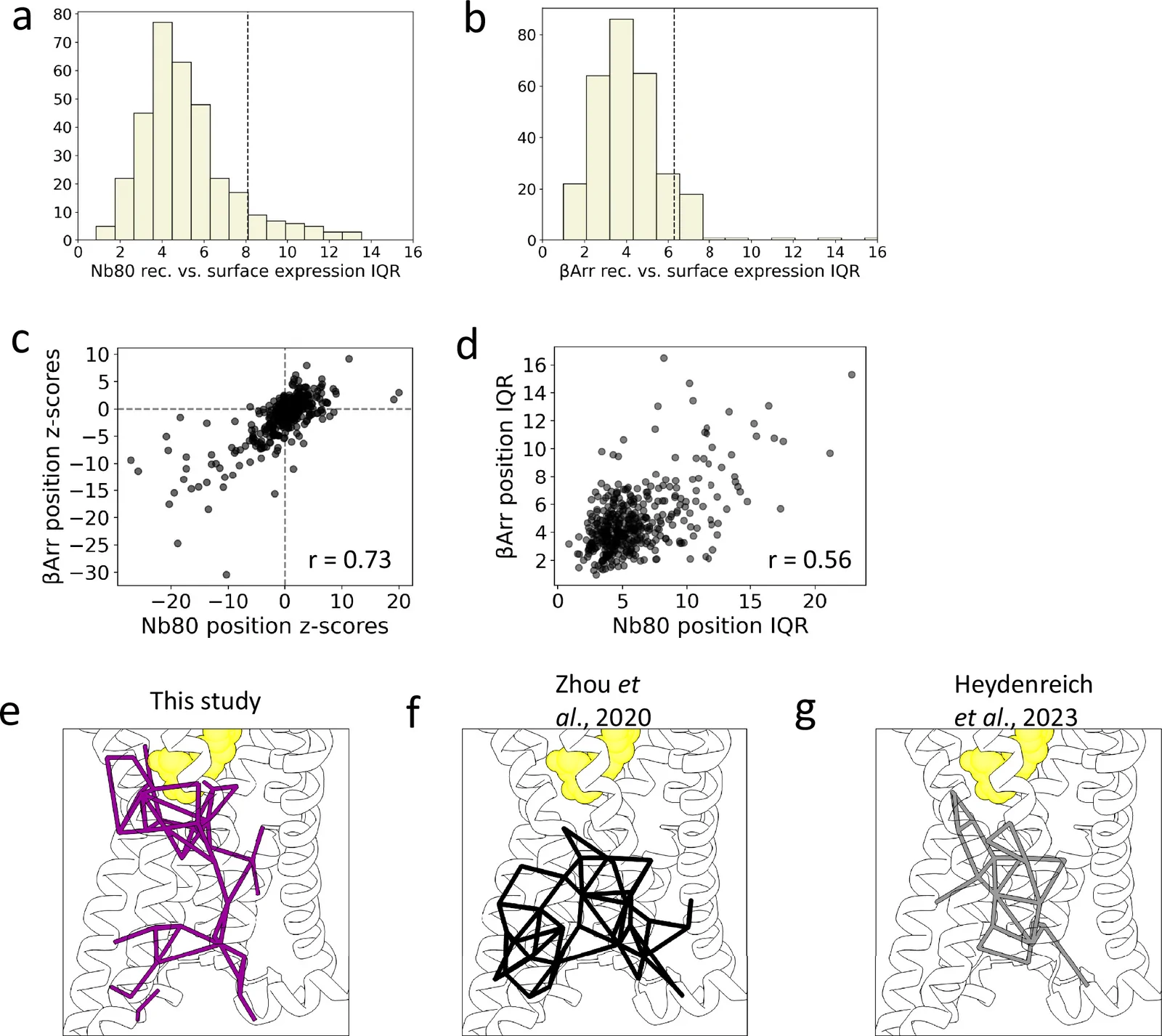
Extended analysis of effects on recruitment beyond those on expression. **a** Distribution of interquartile ranges of z-scores for all residues not in the loss-of-function or gain-of-function categories for Nb80 maximum recruitment. Dashed line indicates the 90^th^ percentile; residues to the right of this line are prioritized as change-of-function. **b** Same as in (a) but for β-Arrestin recruitment. **c** Position-average z-scores for Nb80 and β-Arrestin recruitment. **d** Position interquartile ranges of z-scores for Nb80 and β-Arrestin recruitment. **e** Activation network defined in this study, inter-residue contacts shown as purple bars. **f** Class A GPCR activation network defined by Zhou et al., 2020, inter-residue contacts shown as black bars. **g** β2AR activation network defined by Heydenreich et al. ^18^, inter-residue contacts shown as gray bars. Source data

For both Nb80 and β-arrestin, more residues confer loss of function (LoF) than gain of function (GoF) when substituted and the magnitude of loss is greater than the magnitude of gain (Fig. 2f,g). In total, 49 and 73 residues are LoF for Nb80 and β-arrestin, respectively (false discovery rate (FDR) = 0.1; Supplementary Table 2). Of these, 47 are shared (Fig. 2h; odds ratio (OR) = 304.6, *P* = 2.2 × 10^−40^, two-sided Fisher’s exact test (FET)). An example of a strong LoF residue is D113^3×32^ (Fig. 2i; GPCRdb numbering^45^ in superscript), which participates directly in isoprenaline binding. There are 31 and 53 GoF residues for Nb80 and β-arrestin (FDR = 0.1), respectively, of which 20 are shared (OR = 19.2, *P* = 1.3 × 10^−12^, two-sided FET). E268^6×30^ is the most significant GoF residue (Fig. 2j), consistent with previous work demonstrating constitutively activating substitutions at this site^46^. We also identified residues where substitutions have highly variable effects on recruitment using the interquartile range (IQR) of *z* scores at all positions (Extended Data Fig. 2a,b). After excluding LoF and GoF residues, we categorized those residues with IQR above the 90th percentile as change of function (CoF): 34 and 31 residues for Nb80 and β-arrestin, respectively, with eight shared (Fig. 2h; OR = 2.9, *P* = 0.037). An example of a strong CoF residue is L75^2×46^ (Fig. 2k), which is near the allosteric sodium-binding site and neighbors LoF residue A76^2×47^. Substitutions to small, uncharged residues (G, S, T, A and C) decrease surface expression but increase Nb80 and β-arrestin recruitment relative to the modeled expectation. On the other hand, positively charged residues (H, R and K) are well expressed but strongly decrease recruitment of both proteins.

Average position *z* scores and position IQRs are well correlated (*r* = 0.73 and 0.56, respectively; Extended Data Fig. 2c,d), indicating that many residues contribute similarly to recruitment of both proteins. Plotting the residues identified for both proteins onto the structure (PDB 7DHR) reveals that LoF residues are predominantly within the core of the receptor (Fig. 2l), whereas GoF and CoF residues are more peripheral (Fig. 2m,n).

### LoF residues define a core activation network

**Fig. 3:**
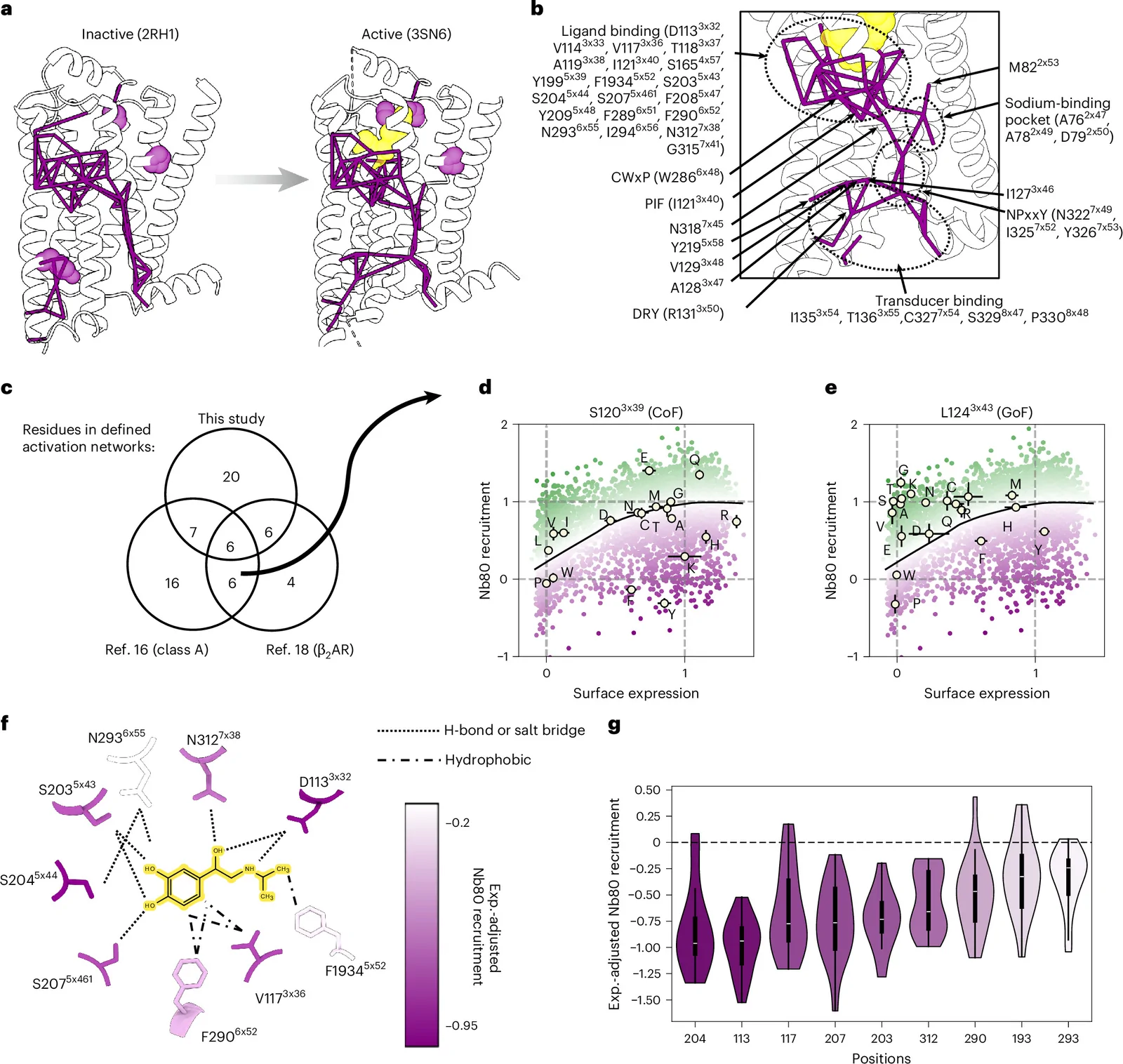
A core activation network links ligands to transducers. **a**, Inactive-state and active-state structures of β_2_AR with LoF residues colored purple. Those LoF residues that make a direct contact with other LoF residues are visualized as pseudobonds connecting them, while disconnected LoF residues are shown with spheres. **b**, Enlarged view of the core activation network with residues labeled and their participation in different structural motifs. **c**, Venn diagram showing the overlap between network residues identified in this work and previous work^16,18^. **d**, Variant surface expression and Nb80 recruitment at S120^3×39^, a CoF residue, are plotted against the population of all variants. Error bars represent the s.e.m. of three experimental replicates. **e**, Same as **d**, but with variants at L124^3×43^, a GoF residue. **f**, The isoprenaline-binding pocket, with isoprenaline highlighted in yellow and the residues making up the binding site colored according to their median-expression-adjusted Nb80 recruitment level. **g**, Violin plot of expression-adjusted Nb80 recruitment at residues in the isoprenaline-binding site. Violins are colored according to their median value, with the same color scale as in **f**. The center value (white circle) represents the median, the box represents the IQR and the whiskers extend to either the most extreme value or 1.5× the IQR, whichever is smaller. Source data

The majority of the shared LoF residues (39 of 47) form an interconnected path linking the ligand-binding site all the way to the transducer-binding site in the active-state receptor structure (Fig. 3a; hereafter referred to as the ‘core activation network’). All nine residues of the isoprenaline-binding pocket are identified (D113^3×32^, V117^3×36^, F193^45×52^, S203^5×43^, S204^5×44^, S207^5×461^, F290^6×52^, N293^6×55^ and N312^7×38^), along with many residues in the second shell of the ligand-binding pocket (that is, residues that contact residues of the binding site; Fig. 3b). These include W286^6×48^ of the CWxP motif and I121^3×40^ of the PIF motif^47,48^. The core activation network then connects to N322^7×49^ of the NPxxY motif^47^ through two pathways; W286^6×48^ contacts N318^7×45^, which then connects to N322^7×49^, while A119^3×38^ goes through A78^2×49^ and D79^2×50^ of the allosteric sodium-binding pocket, which then contacts N322^7×49^. Of the NPxxY motif, N322^7×49^, I325^7×52^ and Y326^7×53^ are LoF. Lastly, in the active-state structure, Y326^7×53^ of the NPxxY motif contacts R131^3×50^ (of the DRY motif) and I127^3×46^ of helix 3. Y326^7×53^ is proposed to make an important water-mediated hydrogen bond with Y219^5.58^ (ref. ^49^), which is also LoF and makes a direct contact with R131^3×50^.

Of the eight LoF residues not connected to the core activation network, three likely directly disrupt transducer binding^42,49^ (P138^34×50^, F139^34×51^ and Y141^34×53^), while three more form disulfide bonds between ECL2 and TM3 (C106^3×25^, C190^ECL2^ and C191^45×50^). F193^45×52^ is a disconnected LoF residue that makes direct contact with bound isoprenaline. P88^2×58^ in TM2 is the last disconnected residue.

Previous work used structural analysis to define an activation network for class A GPCRs^16^ and alanine scanning mutagenesis to define an activation network for β_2_AR^18^. About half of the residues identified in our network are present in at least one of the others (19/39; Fig. 3c). We identify more residues near the ligand-binding site compared to the other networks (Extended Data Fig. 2e–g). Interestingly, of the six residues shared by the other two networks that are not part of our core activation network, we identify four of these as either CoF (S120^3×39^ and F332^8×50^) or GoF (L124^3×43^ and D130^3×43^), emphasizing the value of empirically assaying all 19 substitutions per position. At S120^3×39^, which neighbors I121^3×40^ of the PIF motif, positively charged substitutions (R, K and H) yield stable receptors with significantly reduced recruitment, while E and Q also yield stable receptors but increase recruitment (Fig. 3d). At L124^3×43^, most variants decrease surface expression but increase recruitment, with the exception of the aromatic amino acids (W, F and Y) and proline, which recruit Nb80 less than expected given their expression level (Fig. 3e).

### GoF sites are at the ionic lock, near the NpxxY motif and in the extracellular vestibule

**Extended Data Fig. 3:**
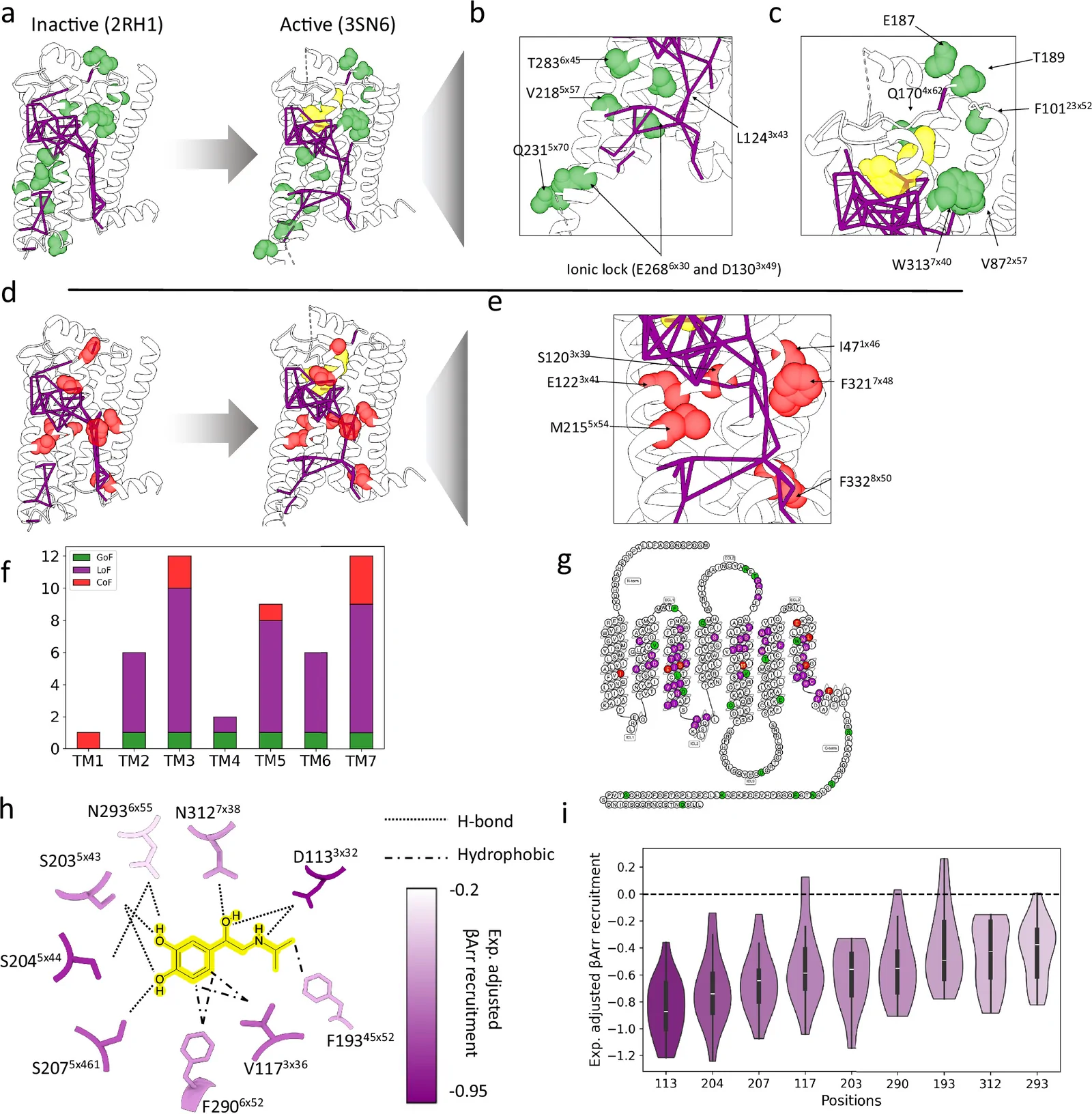
Structural and sequence locations of maximum recruitment gain- and change-of-function residues. **a** Gain-of-function residues colored green on the inactive and active structures, with the core activation network represented as purple sticks. **b** Enlarged view of the ionic lock and surrounding residues in the active structure. **c** Enlarged view of the gain-of-function residues near the orthosteric site. **d** Change-of-function residues colored red on the inactive and active structures, with the core activation network represented as purple sticks. **e** Enlarged view of the change-of-function residues near the PIF and NPxxY motifs. **f** The number of each type of annotated residue in each transmembrane helix. **g** The sequence-based locations of the annotated residues in a snake plot representation (made with GPCRdb). **h** The isoprenaline binding pocket, with isoprenaline highlighted in yellow, and the residues making up the binding site colored according to their median expression adjusted β-arrestin recruitment level. **i** Violin plot of expression adjusted β-arrestin recruitment at the isoprenaline binding site residues. Violins are colored according to their median value, with the same color scale as in (h). Source data

We next examined the location of the GoF residues (Extended Data Fig. 3a,f). Two of the GoF residues, D130^3×49^ and E268^6×30^, participate in the conserved ionic lock (with R131^3×50^ of the DRY motif^50^). While R131^3×50^ of the DRY motif is an LoF residue that contacts Nb80 (ref. ^42^) and is predicted to contact β-arrestin^49^, some substitutions at D130^3×49^ of the DRY motif can constitutively activate the receptor^51^. The other GoF residues are predominantly in two clusters: one consisting of TM3, TM5 and TM6, near the NPxxY and DRY motifs (Extended Data Fig. 3b). L124 sits in the center of the receptor, near the I of PIF motif, DRY motif and NPxxY. A few substitutions at L124^3×43^ have been shown to cause constitutive activity at this site^52^ but we show that most variants activate more than expected (discussed above). The other cluster is near the ligand-binding site and in the extracellular vestibule (Extended Data Fig. 3c). E187 and T189 are in ECL2, where they may affect ligand binding or activation^53^. W313^7×39^ and V87^2×57^ form two van der Waals interactions in the inactive state (PDB 2RH1) but only one in the active state (PDB 3SN6); our data suggest that disrupting this interaction leads to increased recruitment. Lastly, there are seven GoF residues in the C-terminal tail (Extended Data Fig. 3g).

### CoF sites are adjacent to the core network

Plotting the CoF residues onto the inactive and active structures (Extended Data Fig. 3d) reveals that they cluster near important LoF residues that make structural changes during activation. For instance, S120^3×39^ and E122^3×41^ are adjacent to I121^3×40^ of the PIF motif. Likewise, F321^7×48^ is adjacent to N322^7×49^ (LoF) of the NPxxY motif, while F332^8×50^ is nearby (Extended Data Fig. 3e). This proximity to essential switch residues suggests that CoF residues may specifically stabilize or destabilize the active or inactive receptor state depending on the amino acid substitution.

### Differential contribution of residues in the isoprenaline-binding pocket

Next, we examined the effects of substitutions at residues in the isoprenaline-binding site. While all residues are LoF, the magnitude of effects differs. D113^3×32^, which forms two hydrogen bonds with isoprenaline, has very strong effects (median-expression-adjusted Nb80 and β-arrestin recruitment = −0.94 and −0.89, respectively; Fig. 3f,g and Extended Data Fig. 3h,i). N293^6×55^ also forms a hydrogen bond with isoprenaline but substitutions here are the least damaging of the binding site (median-expression-adjusted Nb80 and β-arrestin recruitment = −0.24 and −0.4, respectively). This tolerance is surprising as N293^6×55^ also forms a hydrogen bond with S204^5×44^, which does not directly contact the ligand but has strongly deleterious effects (median-expression-adjusted Nb80 and β-arrestin recruitment = −0.96 and −0.81, respectively). This suggests that the role of S204^5×44^ may be more related to receptor activation than ligand binding.

### Genetic control of recruitment bias

**Fig. 4:**
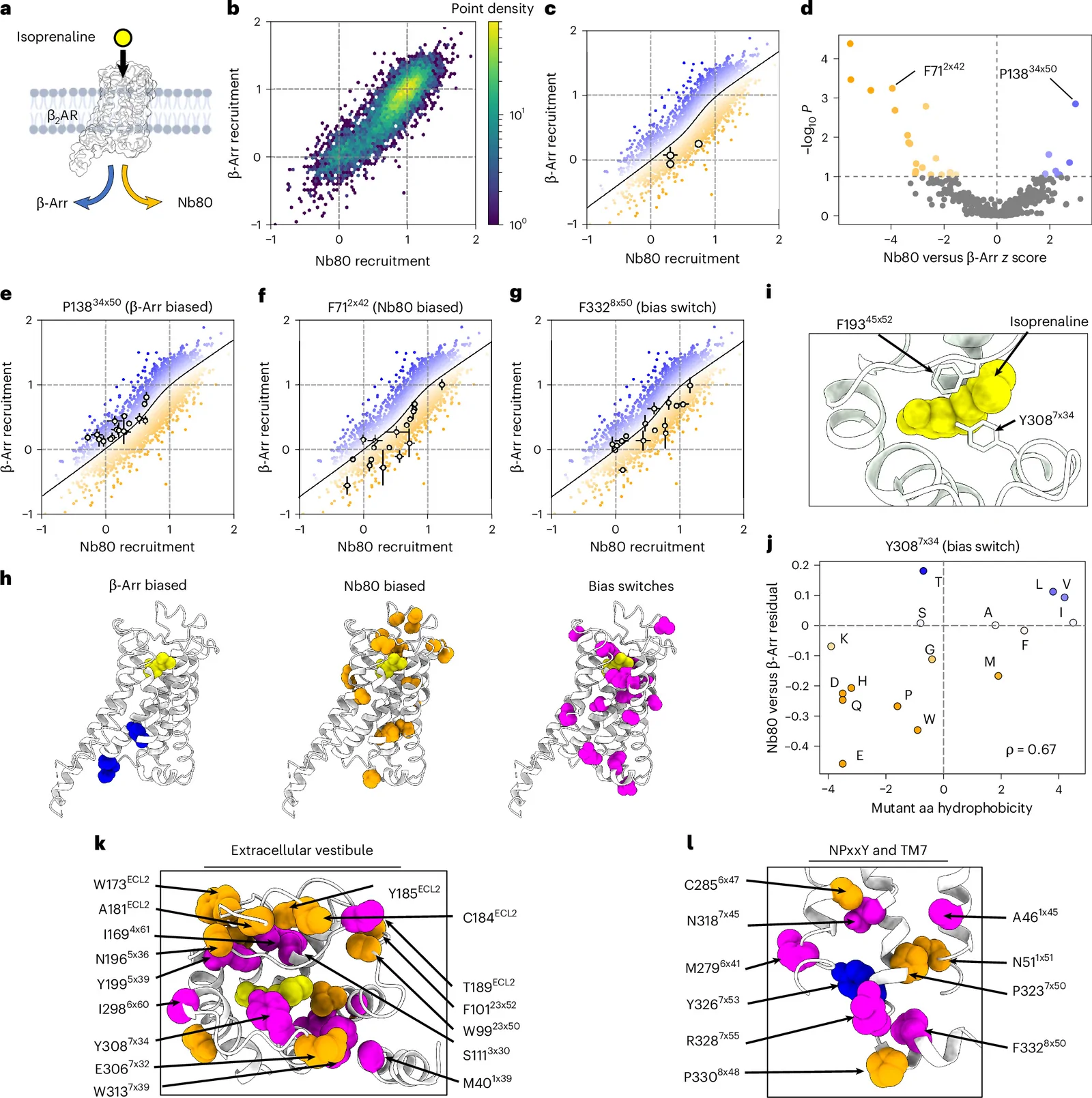
Genetic control of receptor recruitment bias. **a**, Schematic showing β_2_AR activation through β-arrestin and Nb80 in response to isoprenaline. **b**, Nb80 versus β-arrestin recruitment for missense variants (Pearson’s *r* = 0.89, *P* < 10^−100^, *n* = 6,791). **c**, Nb80 versus β-arrestin recruitment with a LOWESS fit and variant scores colored by residual to the fit line. Individual variant scores, with error bars representing the s.e.m. of three experimental replicates, are shown for three variants previously reported to cause biased signaling. **d**, Volcano plot showing the position-average *z* score and the minus log_10_-transformed *P* value (Mann–Whitney *U*-test, two-sided) of all residues for Nb80 versus β-arrestin recruitment. Residues with significantly biased residuals are colored according to the average *z* score. **e**, Nb80 and β-arrestin recruitment effects of variants at P138^34×50^, plotted over the population of variant effects. Error bars represent the s.e.m. of three experimental replicates. **f**, Same as in **e**, but with variants at F71^2×42^. **g**, Same as in **e**, but with variants at F332^8×50^. **h**, β-Arrestin-biased, Nb80-biased or bias switch residues highlighted on the solved structure (PDB 7DHR). Isoprenaline is shown in yellow. **i**, F193^45×52^ and Y318^7×34^ form a lid over isoprenaline in the solved structure (PDB 7DHR). **j**, Hydrophobicity (Kyte–Doolittle scale) of the mutant amino acid compared to the Nb80 versus β-arrestin recruitment bias. **k**, Enlarged view of the extracellular vestibule. Colored residues are significantly Nb80-biased (orange), β-arrestin-biased (blue) or bias switches (magenta). **l**, Enlarged view of NPxxY motif and TM7. Residues are colored as in **k**. Source data

**Extended Data Fig. 4:**
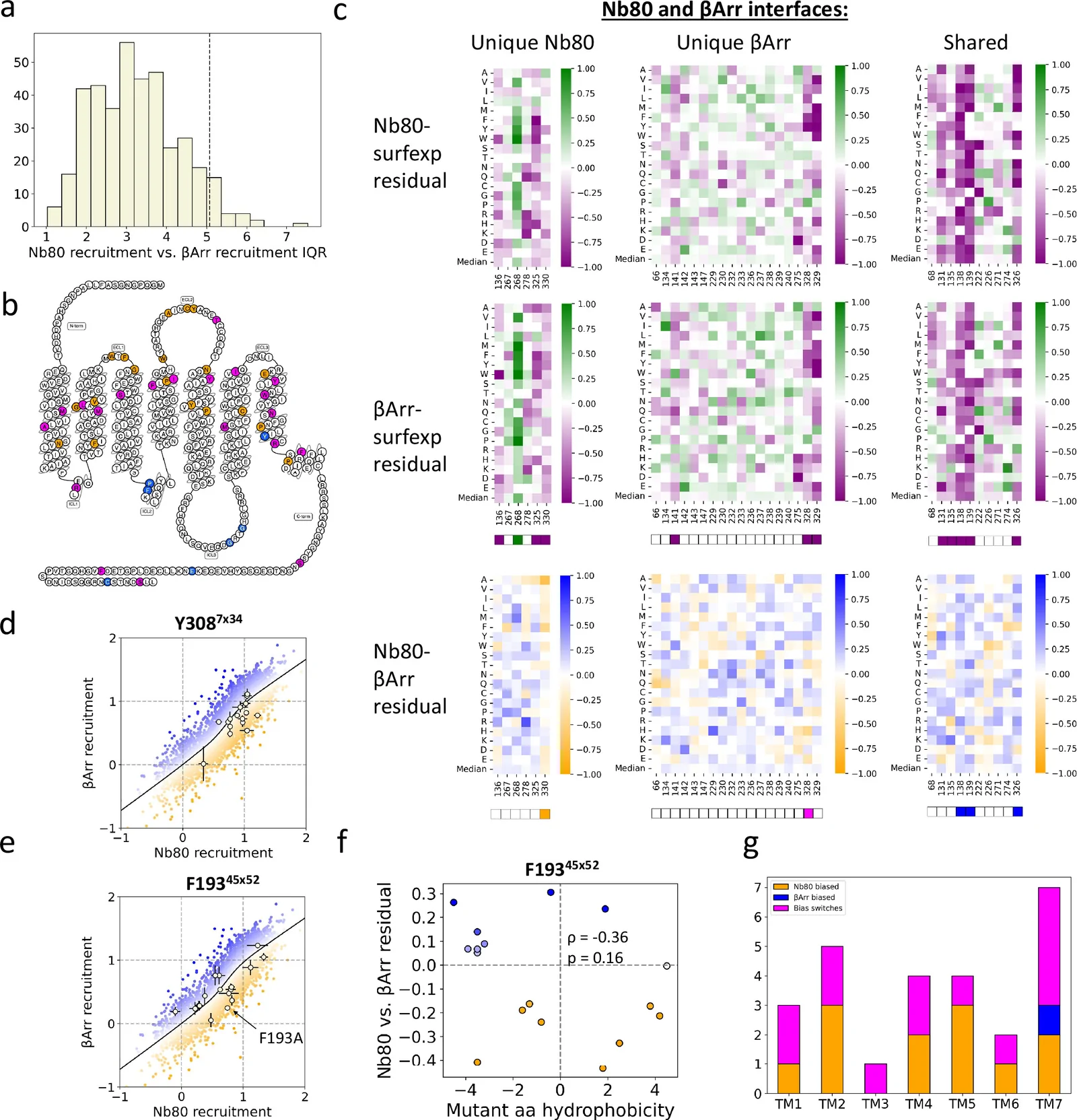
Extended analysis of biased recruitment. **a** Interquartile range of Nb80 to β-arrestin z-scores for all residues not called as Nb80 or β-Arrestin-biased. Dashed line indicates the 95^th^ percentile of IQRs; residues with IQR greater than this line are considered as change-of-function. **b** Snakeplot representation showing the location of Nb80-biased, β-Arrestin-biased, and bias switch residues. **c** Mutation effects in the binding interfaces between β2AR and Nb80 and β-Arrestin. Shown are expression adjusted recruitment effects through both transducers, split into unique Nb80, unique β-Arrestin, or residues shared in both binding interfaces. At the bottom are the biased recruitment residuals for those same groups. Beneath heatmaps are indicated the residues annotated as maximum recruitment LoF, GoF, or CoF (purple, green, or red), and residues annotated as Nb80 biased, β-Arrestin-biased, or bias switches (orange, blue, or magenta. **d** Mutation effects at Y308^7×34^ shown, with the population shown in the background and colored according to residual from the LOWESS fit line. Error bars represent standard error of the mean. **e** Same as in (d) but for F193^45×52^. **f** Hydrophobicity of the mutant amino acid compared to the Nb80 vs. β-Arrestin residual for mutations at position F193^45×52^. **g** The number of each type of annotated residue in each transmembrane helix. Source data

Next, we directly compared mutational effects on Nb80 and β-arrestin recruitment (Fig. 4a). Mutational effects on recruitment of Nb80 and β-arrestin are well correlated (Pearson’s *r* = 0.89; Fig. 4b). Single mutants previously shown to bias the receptor toward G-protein signaling (F193A (ref. ^54^), Y219A (ref. ^55^) and Y199A (ref. ^56^)) are indeed biased toward Nb80 in our experiments (Fig. 4c). To identify residues with stronger effects on one pathway, we again fitted a LOWESS regression curve to the data, calculated residuals and *z* scores for all variants in the dataset and compared *z* scores of substitutions at each position to *z* scores of substitutions at all others. This identifies 19 residues where substitutions have greater effects on β-arrestin recruitment and seven positions where substitutions have significantly greater effects on Nb80 recruitment (FDR = 0.1; Fig. 4d and Supplementary Table 3). We also compared the IQR of all remaining residues to find sites where variants have highly variable effects on bias, which we call ‘bias switches’. To more closely match the number of biased residues, we prioritized the top 5% of residues as bias switches (Extended Data Fig. 4a).

Examples of strongly biased residues are shown (Fig. 4e,f), along with a a strong bias-switching residue (Fig. 4g). One expectation is that substitutions at residues making direct contacts with either Nb80 or β-arrestin could result in biased recruitment. Nb80 and β-arrestin have overlapping but distinct binding interactions with the intracellular portion of β_2_AR. Nb80 forms direct contacts with 16 residues in TM3, TM5, TM6 and TM7 (ref. ^42^). The β-arrestin-binding interface is not experimentally determined but predicted to be larger^49^, encompassing 28 residues across ICL1, ICL2, ICL3, TM6 and TM7, with ten residues overlapping the Nb80 interface (Extended Data Fig. 4c).

Three of the seven residues biased toward β-arrestin recruitment make direct binding interactions with both Nb80 and β-arrestin (P138^34×50^, F139^34×51^ and Y326^7×53^), suggesting a more critical role for these residues in binding Nb80. The other four β-arrestin-biased residues are in either ICL3 (G252 and G255) or the C-terminal tail (E373 and C406) (Extended Data Fig. 4b). The only residue biased toward Nb80 within either binding interface is P330^8×48^, which is predicted to interact with Nb80 but not β-arrestin. There is also one bias switch residue, R328^7×55^, that is predicted to only bind β-arrestin. This relative sparsity of bias effects suggests that few residues at the transducer interface contribute substantially to the recruitment of each protein (Extended Data Fig. 4c).

### The extracellular vestibule allosterically controls recruitment bias

In contrast, there are many residues biased toward Nb80 recruitment located away from the orthosteric site in the extracellular vestibule: W99^23×50^, F101^23×52^, G102^3×21^, W173^ECL2^, A181^ECL2^, C184 ^ECL2^, Y185 ^ECL2^, N196^5×36^ and E306^7×32^ (Fig. 4h,k). Considered together, the ECLs are enriched in Nb80-biased residues compared to the whole receptor (OR = 6.4, *P* = 1.0 × 10^−3^, one-sided FET); in particular, ECL2 is enriched for Nb80-biased residues (OR = 5.6, *P* = 0.014, one-sided FET).

The importance of the extracellular vestibule for Nb80-biased activation in our data is consistent with structural data. Salmeterol is a G-protein-biased β_2_AR agonist that binds the orthosteric site in a similar way to the endogenous ligand (epinephrine) except that it has a long aryloxyalkyl tail that extends into the extracellular vestibule, where it interacts extensively with ECL2 (ref. ^57^). Furthermore, comparative structural analyses of other GPCRs have suggested that the extracellular vestibule adopts a more constricted conformation in the β-arrestin-bound state compared to the G-protein-bound state^58^. Taken together, these data point to the extracellular vestibule, particularly ECL2, as hotspots for G-protein-biased agonism in β_2_AR.

Among the bias-switching residues, the second strongest is the vestibule residue, Y308^7×34^ (Extended Data Fig. 4d). This residue has been proposed to be necessary for G-protein signaling^59^, in addition to forming a hydrophobic gate with F193^45×52^ that interacts with and dehydrates ligand molecules as they traverse the vestibule and approach the orthosteric binding site^60^. In the active state, these two residues form a ‘lid‘ over the ligand, with F193^45×52^ making a direct contact with isoprenaline (Fig. 4i). On the basis of the alanine substitution, F193^45×52^ was postulated to coordinate β-arrestin signaling^54^. Interestingly, while this single variant does have a stronger effect on β-arrestin recruitment, other variants at the same site have the opposite effect (Extended Data Fig. 4e). We examined the effects of substitutions at these two sites and found that recruitment bias is correlated with mutant hydrophobicity for Y308^7×34^ (*ρ* = 0.67, *P* = 4.3 × 10^−3^; Fig. 4j) but not for F193^45×52^ (*ρ* = −0.36, *P* = 0.16; Extended Data Fig. 4f). This suggests that the role of Y308^7×34^ in determining bias is directly tied to its hydrophobicity, while the relationship of F193^45×52^ to bias is more nuanced.

### Recruitment bias in TM7

There are also multiple bias residues in or near the NPxxY motif of TM7 (Fig. 4l) with TM7 enriched for bias residues (OR = 3.7, *P* = 0.011, one-sided FET; Extended Data Fig. 4g). P323^7×50^ is not predicted to contact directly β-arrestin^49^, suggesting that the Nb80 bias of the residue cannot be explained simply by disrupting a direct contact. N318^7×45^, F332^8×50^ and R328^7×55^ are all bias switches near the NPxxY motif (Fig. 4k). A role for TM7 in determining bias is consistent with nuclear magnetic resonance experiments demonstrating changes in TM7 conformation upon binding of biased agonists^61^.

### Activation functions for >7,500 β_2_AR variants

The data presented thus far quantify recruitment to β_2_AR with saturating levels of isoprenaline. However, because of its pooled design and RNA-seq-based output, GPCR-MAPS can also be used to quantify the full concentration–response curves (activation functions) of all receptor variants in a library.

**Fig. 5:**
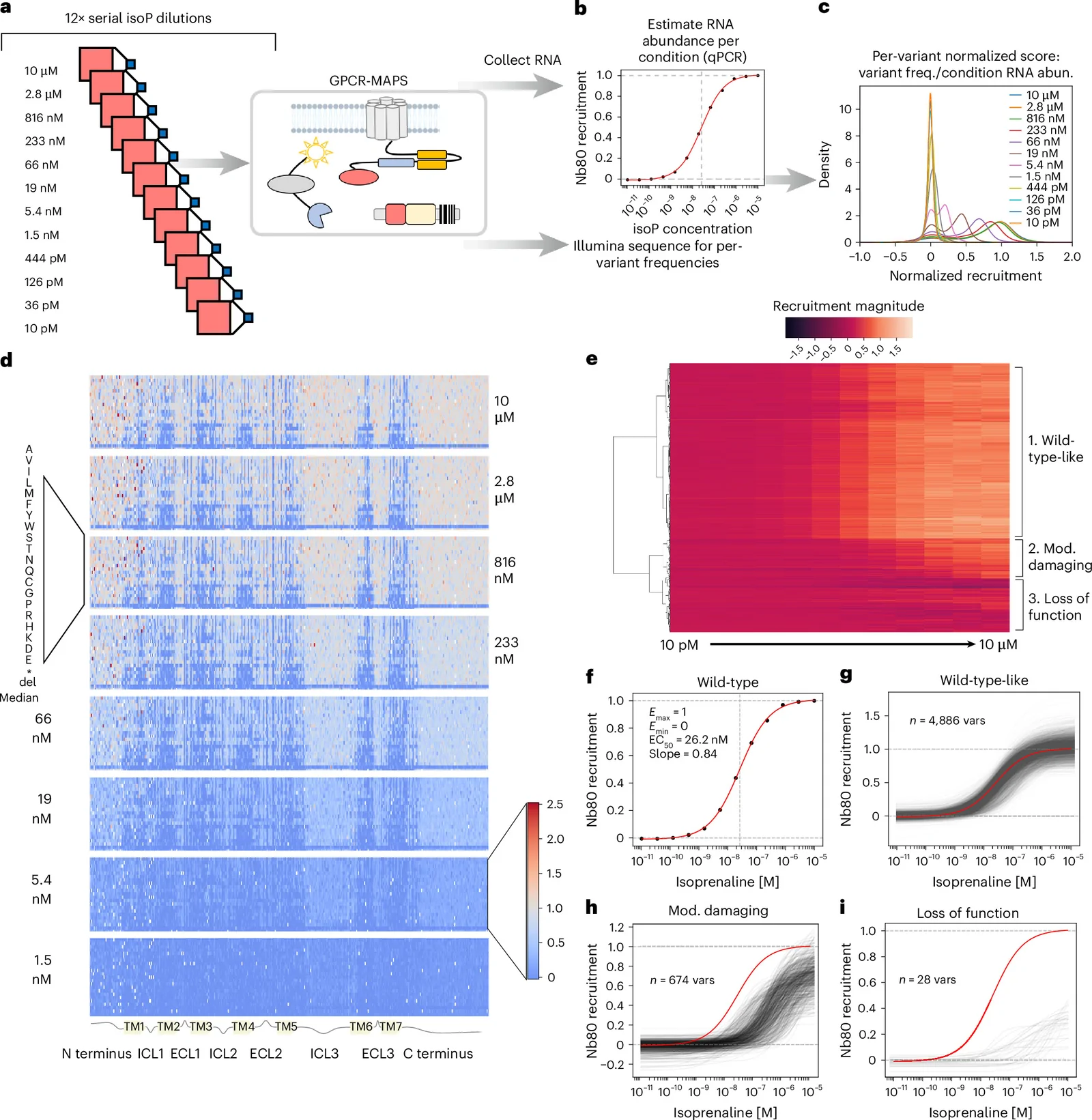
Quantifying >7,500 concentration–response relationships. **a**, Experimental design: serial dilutions of isoprenaline (isoP) were used to stimulate landing pad cells expressing the β_2_AR library. Then, the GPCR-MAPS recruitment assay was performed and RNA was collected from the stimulated cells. **b**, The RNA abundance from each sample was first quantified with qPCR and then the variant frequencies were estimated with short-read sequencing. **c**, Per-variant normalized scores were then calculated by normalizing variant frequencies (freq.) in each condition by the RNA abundance (abun.) of each condition. **d**, Heat map representations of the eight highest-concentration drug treatment conditions. β_2_AR positions are shown on the *x* axis; substitution identities are shown on the *y* axis. One *y* axis is blown up; the remaining heat maps follow this same scheme. Gray cells indicate wild-type-like recruitment; blue and red indicate reduced and increased recruitment, respectively. One color bar is blown up; the remaining heat maps follow this same scheme. The asterisk indicates the stop codon. **e**, Clustered heat map of recruitment intensities. The color bar is shown on top. Three main clusters correspond to wild-type-like, moderately (mod.) damaging and complete LoF curves. **f**, The curve fit to the wild-type data, with *E*_max_, *E*_min_, EC_50_ and slope indicated. **g**, Fit curves from the wild-type-like cluster in black, with the wild-type curve in red. **h**, Fit curves from the moderately damaging cluster in black, with the wild-type curve in red. **i**, Fit curves from the LoF cluster in black, with the wild-type curve in red. Source data

**Extended Data Fig. 5:**
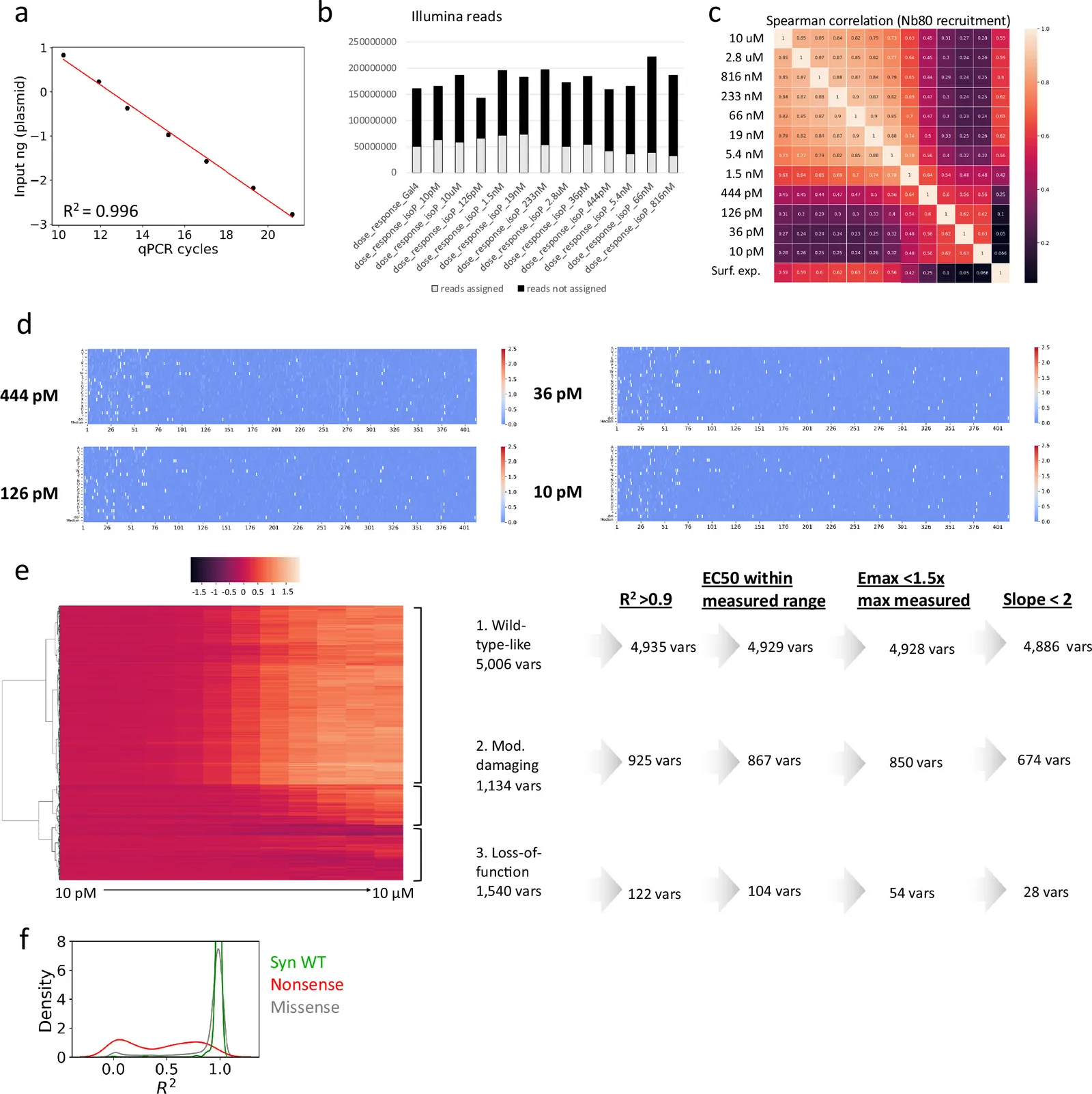
Extended concentration-response analysis. **a** To measure RNA abundance, a standard curve was generated using serial dilutions of plasmid DNA and amplifying the Citrine reporter construct. **b** The number of Illumina reads acquired for each sample, with black and gray portions of bars indicating the fraction of reads that were assigned or not to barcodes. **c** Spearman correlations of variant scores in the serial dilution conditions or the surface expression experiment. **d** Heatmap representations of the recruitment data from the four lowest concentration conditions. **e** Clustered heatmap representations of the 7,680 measured dose response relationships. To the right are the quality control heuristics and the number of variants that pass each filter step from each cluster. **f** R^2^ goodness-of-fit estimates for the curves fit to synonymous wild-type, nonsense, or missense variants. Source data

To demonstrate this capability, we performed the Nb80 GPCR-MAPS recruitment assay with 12 serially diluted concentrations of isoprenaline, ranging from 10 pM to 10 μM (Fig. 5a). To compare the magnitude of recruitment across experimental conditions, following RNA isolation, we performed qPCR to estimate the total amount of reporter transcription (Fig. 5b, Extended Data Fig. 5a and Methods). Then, by deep sequencing the barcodes and normalizing the frequencies by the estimated total reporter RNA, we derived normalized scores across the 12 concentrations (Fig. 5c, Extended Data Fig. 5b and Methods). This allowed us to quantify concentration–response relationships for 7,680 different receptor variants, a total of ~92,000 measurements (Fig. 5d and Extended Data Fig. 5c,d). Clustering the induction dataset revealed three predominant clusters: wild-type-like, moderately damaging and strong LoF (Fig. 5e).

**Fig. 6:**
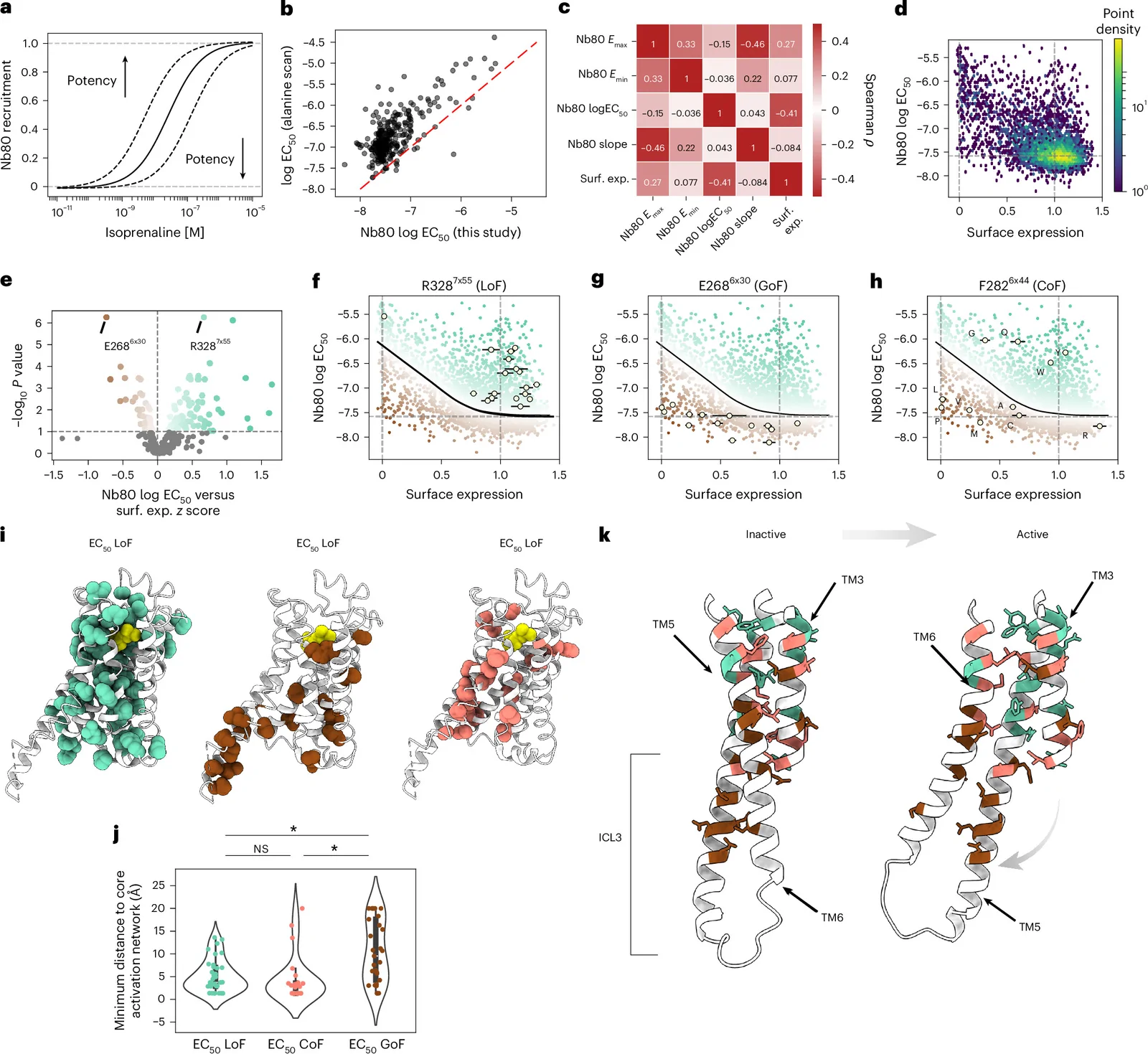
Genetic determinants of potency. **a**, Schematic showing example curves for increased and decreased potency. **b**, Comparison of variant EC_50_ estimates from this study to previously published estimates^18^. The red dashed line is the *x* = *y* line. **c**, Spearman correlations of curve-fitting parameter estimates and surface expression scores. **d**, Surface expression scores compared to log-transformed EC_50_ estimates (Spearman’s *ρ* = −0.41, *P* < 10^−100^). **e**, Volcano plot showing the position-average *z* score and the −log_10_-transformed *P* value (Mann–Whitney *U*-test, two-sided) of all residues for log EC_50_ versus surface expression. **f**, The log-transformed EC_50_ versus surface expression for variants at R328^7×55^, plotted over the population of variant effects. Error bars represent the s.e.m. of three experimental replicates. **g**, Same as in **f**, but with variants at E268^6×30^. **h**, Same as in **f**, but with variants at F282^6×44^. **i**, EC_50_ of LoF, GoF or CoF residues highlighted on the solved structure (PDB 7DHR). Isoprenaline is shown in yellow. **j**, Violin plots showing minimum distance of LoF, GoF or CoF residues from the core activation network in AlphaFold-Multistate active-state model. Distances were capped at 20 Å (*n* = 36, 23 and 29 for LoF, CoF and GoF residues, respectively). The center value (white circle) represents the median, the box represents the IQR and the whiskers extend to either the most extreme value or 1.5× the IQR, whichever is smaller. *P < 0.05; NS, not significant. **k**, Portions of TM3, TM5 and TM6, as predicted by AlphaFold-Multistate and downloaded from GPCRdb^45^. LoF residues are colored aquamarine, GoF residues are colored brown and CoF residues are colored salmon. Source data

Next, we fit a four-parameter Hill curve to the wild-type genotype, normalizing the lower asymptote (*E*_min_) and the upper asymptote (*E*_max_) to 0 and 1, respectively, and deriving a half-maximal response (EC_50_) estimate of 26.2 nM and slope (Hill coefficient) of 0.84 (Fig. 5f). Then, we fit curves to all variants; after quality control (Extended Data Fig. 5e,f), this resulted in 5,588 high-quality concentration–response curves with well-estimated parameters (Fig. 5g–i). We note that variants with reduced activation level are more poorly fit and, therefore, likelier to be filtered (Extended Data Fig. 5e). Our EC_50_ measurements correlate well with the results of individually assayed substitutions^18^ (Pearson’s *r* = 0.74, *n* = 317 variants; Fig. 6b).

### Surface expression is a major determinant of receptor potency

To investigate determinants of potency (EC_50_), we first quantified the correlations among the four Hill function parameters, as well as their dependence upon surface expression. The strongest relationships we found were between slope and *E*_max_ (*ρ* = −0.46) and between surface expression and log EC_50_ (*ρ* = −0.41) (Fig. 6c,d). Surface expression level is, thus, a major influence on potency in the β_2_AR. Therefore, to mechanistically understand changes in potency, it is necessary to again quantitatively deconvolve direct mutational effects from those mediated by changes in receptor expression.

### Potency tuning

**Extended Data Fig. 6:**
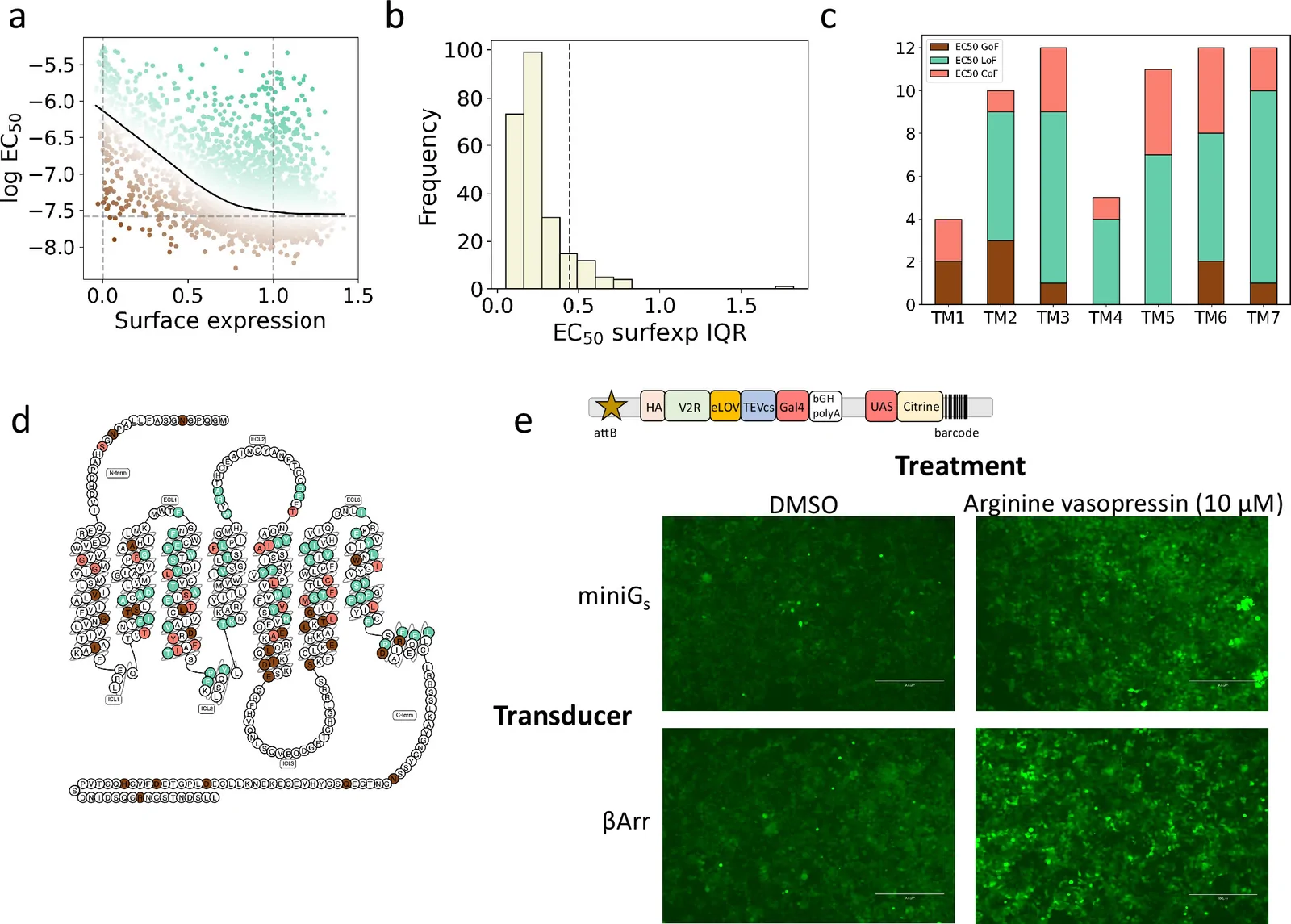
Extended potency analysis. **a** Log-transformed EC_50_ compared with surface expression estimates, fit with a LOWESS regression and with variants colored by the residual to that line. **b** Distribution of position interquartile ranges of EC_50_ to surface expression residuals for positions not identified as loss- or gain-of-function and with at least 10 variants with high quality dose response curves. **c** The number of each type of annotated residue in each transmembrane helix. **d** Snakeplot representation showing the location of EC_50_ LoF, EC_50_ GoF, and EC_50_ CoF residues. **e** A library of vasopressin 2 receptor (V2R) variants were cloned into the same receptor construct as β2AR, then integrated into the landing pad, then cells were transiently transfected with either the miniG_s_ or β-arrestin transducer constructs. Then, cells were treated with either DMSO or 10 μM arginine vasopressin, and 24 h later the cells were imaged for expression of the fluorescent Citrine reporter. Scale bar is 300 μm. Source data

We again used LOWESS regression to model the relationship between surface expression and EC_50_ (Extended Data Fig. 6a), with the residuals to that model identifying positions where substitutions have consistently higher or lower EC_50_ than expected from their impact on expression. A total of 29 residues were identified as GoF (that is, lower EC_50_ than expected) and 59 were identified as LoF (that is, higher EC_50_ than expected) (FDR = 0.1; Fig. 6e,f). There is overlap between these sets and the sets identified from the saturating concentration experiment (23/59 LoF shared, 4/29 GoF shared). Lastly, the IQR was used to identify 29 EC_50_ CoF residues (one shared with saturating concentration experiment; Extended Data Fig. 6b and Supplementary Table 4).

In principle, substitutions could affect potency beyond changes in expression by changing the affinity of isoprenaline binding, or by affecting the probability of isoprenaline-bound receptor to adopt the active conformation^62^. While substitutions at residues making direct contact with isoprenaline would be expected to reduce affinity, substitutions at these sites mostly totally abolish activation; concentration–response curves could only be fit to 39 of 165 missense variants at these sites. However, these 39 variants do have over an order of magnitude reduced potency (median EC_50_ = 359 nM, compared to wild type, 26.2 nM).

Interestingly, EC_50_ LoF residues are enriched in the second shell (15/59 LoF sites (25.4%), OR = 4.12, *P* = 1.9 × 10^−4^, FET). While not statistically enriched, a higher fraction of EC_50_ CoF sites (5/24, 20.8%) are in the second shell compared to only one of 29 GoF residues (3.4%) (Fig. 6i). Whereas substitutions in residues directly contacting the ligand have strong effects on affinity and thereby inactivate recruitment, substitutions in second shell residues can have more moderate effects and modulate the response. These effects are, by definition, allosteric, either indirectly altering ligand-binding affinity or altering the consequences of ligand engagement. Substitutions in the second shell are more likely to reduce affinity than increase it.

One way to disrupt activation could be interfering with the core activation network. Therefore, we quantified the distance of EC_50_-affecting residues to this network and found that LoF and CoF residues are closer in three-dimensional space than GoF residues (*P* = 7.4 × 10^−4^ and 1.3 × 10^−3^, respectively, two-sided Mann–Whitney *U*-test; Fig. 6j). Taken together, as with the maximum recruitment analysis, these data suggest that substitutions that reduce potency do so by perturbing the core activation pathway whereas the mechanism of action of GoF substitutions is likely distinct.

Three of the four most significant EC_50_ GoF residues are E268^6×30^ (Fig. 6g), D130^3×49^ and L272^6×34^. E268^6×30^ and D130^3×49^ were already identified in the maximum recruitment analysis and form the conserved ionic lock while L272^6×34^ is in close proximity, with substitutions thought to destabilize the inactive state^62^. Nearby E268^6×30^ in ICL3, there are nine EC_50_ GoF residues (OR = 3.38, *P* = 7.3 × 10^−3^, one-sided FET). This loop connects TM5 and TM6, which move upon receptor activation (Fig. 6k). These GoF residues could act in a similar way to D130^3×49^, E268^6×30^ and L272^6×34^, weakening the interaction between TM3 and TM6 or other helices. Alternatively, previous studies suggest that ICL3 may have an autoinhibitory role that sterically occludes transducer binding in the inactive state, and some substitutions can abrogate this autoinhibitory effect^63^. Additionally, there are two GoF residues in the N terminus that coincide with the two *N*-glycosylation sites^64^ (N5 and N15; Extended Data Fig. 6d) suggesting that loss of these glycosylation modifications increases receptor potency.

F282^6×44^ of the PIF motif, which undergoes structural rearrangement upon activation, is the strongest EC_50_ CoF residue (Fig. 6h), consistent with previous data showing multiple constitutive activating substitutions and at least one attenuating substitution^65^. Changing the F to other aromatics (Y or W) at position 282 yields stable receptors with strongly reduced potency, suggesting that these substitutions could stabilize the inactive state of the receptor (Fig. 6h). Other substitutions change expression-adjusted potency in either direction, suggesting idiosyncratic effects on the active and/or inactive conformations. Likewise, S120^3×39^ and L212^5×51^ are identified as CoF and are directly adjacent to the other two members of the PIF motif (I121^3×40^ and P211^5×50^), reinforcing that some perturbations to this key switch motif can result in gain or loss of recruitment. There are also several EC_50_ CoF residues in TM5 and TM6 (four in each; Extended Data Fig. 6c), reflecting the key role of these helices in activation.

### Known allosteric pockets

**Fig. 7:**
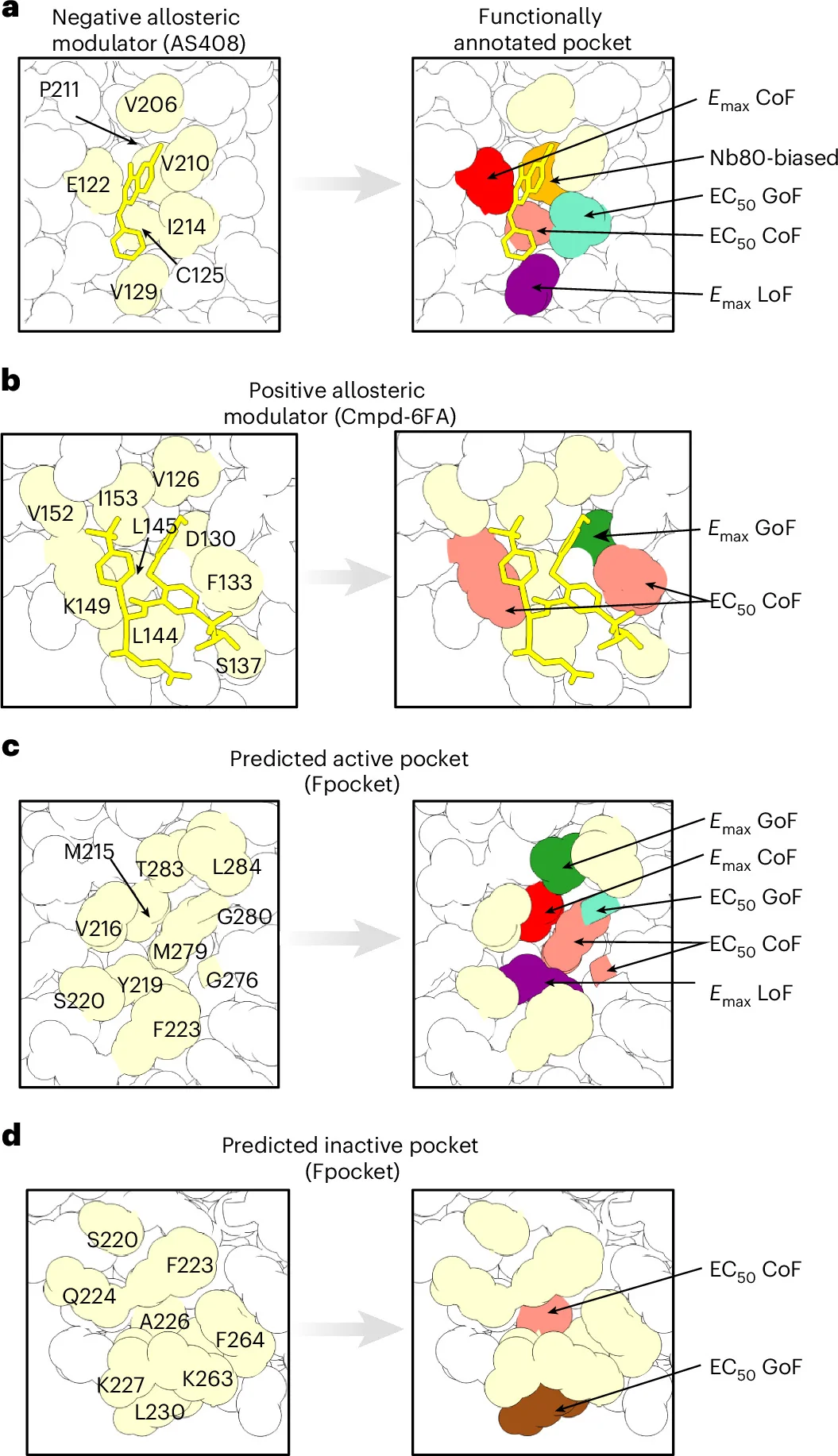
Rationalization and prioritization of allosteric pockets. **a**, Left: the binding pocket of known NAM, AS408, shown in yellow (PDB 6OBA). Residues making direct contact with the NAM are colored beige. Right: the same binding pocket, with residues colored and labeled according to their functional annotation. **b**, Left: the binding pocket of known PAM, Cmpd-6FA, shown in yellow (PDB 6N48). Residues making direct contact with the NAM are colored beige. **c**, Left: a predicted allosteric pocket predicted to be active in PDB 2RH1. Right: the same binding pocket, with residues colored and labeled according to their functional annotation. **d**, Left: a predicted allosteric pocket predicted to be inactive in PDB 2RH1. Right: the same binding pocket, with residues colored and labeled according to their functional annotation.

**Extended Data Fig. 7:**
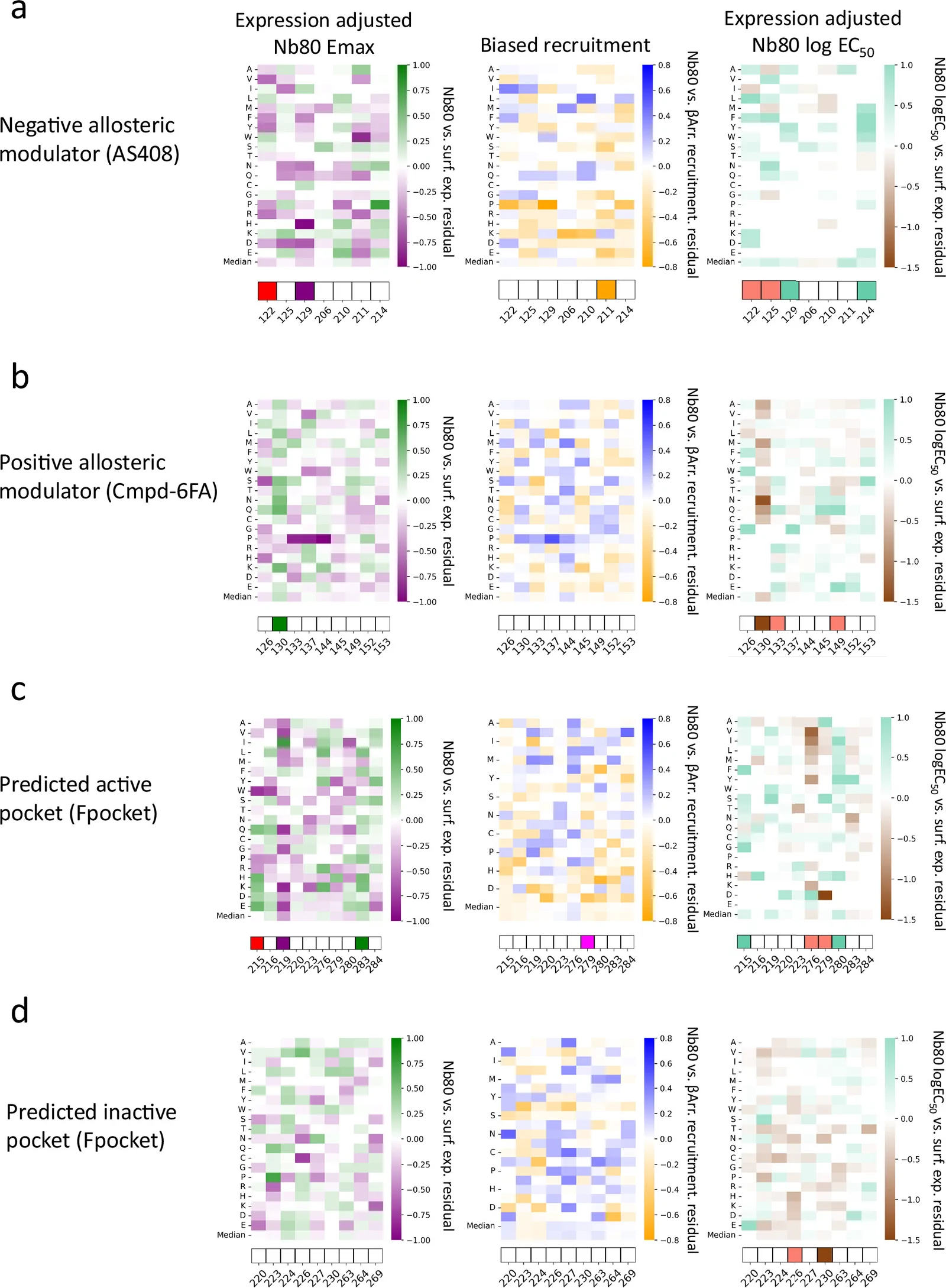
Extended pocket annotation data. **a** Expression adjusted maximum recruitment through Nb80, recruitment bias, and expression adjusted potency of the mutations at sites making up the binding pocket of AS408. Shown below the heatmaps are the categorical annotations of significant residues: purple, green, and red indicate LoF, GoF, and CoF residues for maximum recruitment. Orange, blue, and magenta indicate Nb80 biased, β-Arrestin, biased, or bias switches. Aquamarine, brown, and salmon indicate LoF, GoF, and CoF for potency. **b** Same as in (a) but for the positive allosteric modulator Cmpd-6FA, with structure 6N48. **c** Same as in (a) but for a predicted active pocket in structure 2RH1. **d** Same as in (a) but for a predicted inactive pocket in structure 2RH1. Source data

Lastly, similar to previous work with alanine scan data^18^, we used our β_2_AR data to illustrate how GPCR-MAPS can be used to functionally annotate and prioritize potential pockets in receptors. AS408 is a negative allosteric modulator (NAM) that binds away from the orthosteric and transducer-binding sites, between TM3 and TM5 (ref. ^66^) (Fig. 7a). Across the three analyses (maximum recruitment, biased recruitment and EC_50_), the seven residues making up this pocket have seven total annotations, compared to 96 annotations for all 220 structurally resolved surface-exposed residues (>10% relative solvent-accessible surface area in PDB 2RH1), indicating a functionally important pocket. In particular, V129^3×48^ is LoF for maximum recruitment and EC_50_, while E122^3×41^ is CoF for maximum recruitment and EC_50_. In addition, C125^3×44^ is CoF for EC_50_ and I214^5×53^ is LoF for EC_50_. Substitutions at P211^5×50^ are biased toward Nb80 recruitment, although AS408 is not thought to be a biased NAM (Extended Data Fig. 7a).

Cmpd-6FA, a positive allosteric modulator^67^ (PAM), binds in a pocket formed by nine residues from TM2, TM3, TM4 and ICL2 (Fig. 7b) with only four annotations. However, one of the residues is D130^3×49^, where substitutions cause strong GoF for both maximum recruitment and EC_50_ (Extended Data Fig. 7b), potentially explaining the PAM activity of Cmpd-6FA. Additionally, while Cmpd-6FA mostly forms hydrophobic interactions with β_2_AR, it does form one hydrogen bond with K149^4×41^, which is an EC_50_ CoF residue. Taken together, our functional annotations are consistent with the activities of the two known β_2_AR allosteric modulators.

### Predicted allosteric pockets

To explore prospective functional annotation, we used Fpocket^68^ to predict pockets in the inactive β_2_AR (PDB 2RH1). One pocket, between TM5 and TM6 (Fig. 7c), has eight functional annotations among ten residues, with most being maximum recruitment or EC_50_ LoF or CoF, in addition to one GoF residue (Extended Data Fig. 7c), suggesting a highly active pocket. Indeed, this pocket overlaps with orphan site 5 of the GPCR ‘pocketome’ dataset^33^. This pocket was proposed to be allosterically active on the basis of a subset of substitutions within the pocket affecting receptor activation; however, substitution effects on receptor abundance were not ruled out. An allosteric agonist was also recently found to bind and activate another GPCR, GPR88, at this same site^69^.

On the other hand, a nearby and partially overlapping pocket also between TM5 and TM6 (Fig. 7d) has only two annotations among nine residues (Extended Data Fig. 7d), suggesting that perturbation of this pocket is less likely to have functional effects on receptor activation.

## Discussion

We presented here GPCR-MAPS, a general, modular and massively scalable approach to precisely quantify the effects of substitutions on the activity and bias of GPCRs. GPCR-MAPS makes multiple important technical advancements over existing technologies^27–30^.

First, GPCR-MAPS quantifies the recruitment of proteins to receptors, minimizing the confounding effects of nonlinear and unequal signal amplification, as well as feedback by endogenous and cell-type-specific signaling networks. Second, GPCR-MAPS is modular; we demonstrate here the use of a nanobody, a mini-G protein and β-arrestin. The receptor-proximal readout also facilitates comparisons across these recruited proteins, which can otherwise be confounded by differential signal amplification and crosstalk^70^. To further demonstrate generalizability, we also show the vasopressin 2 receptor recruitment of mini-G_s_ and β-arrestin in response to arginine vasopressin (Extended Data Fig. 6e). Third, GPCR-MAPS measures and quantitatively deconvolves the large confounding influence of expression changes on signaling parameters. Fourth, GPCR-MAPS extends GPCR mutational scanning to the quantification of full concentration–response curve activation functions for all substitutions. Comparing our data to previous β_2_AR mutagenesis studies reveals the importance of all four of these innovations (Extended Data Fig. 1i–k and Fig. 6b).

Applied to β_2_AR, GPCR-MAPS charts a complete amino acid resolution picture of how quantitative multimodal receptor phenotypes respond to perturbations across the entire structure. After deconvolving mutational effects on expression, residues that disrupt recruitment form a connected core activation network linking the ligand-binding site to the transducer coupling site. The data also identify the extracellular vestibule, particularly ECL2, as a major allosteric site to control receptor bias and TM7 near the NPxxY motif as an internal region that allosterically controls receptor activation bias.

**Extended Data Fig. 8:**
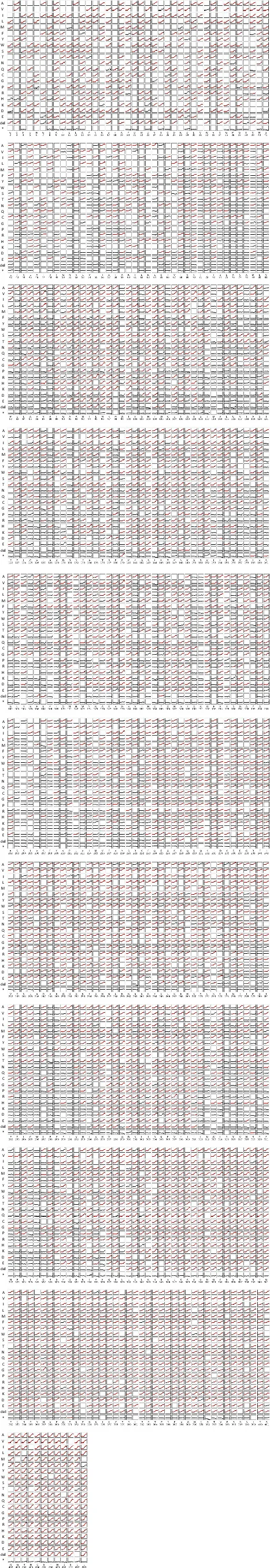
Full set of concentration response curves. β2AR positions on the x-axis and variant identity on the y-axis. Cells without any data were filtered based on too low read count; cells with only black points passed the read-count filter but did not pass the curve fitting heuristic quality controls, cells with black points and red curves are the high-confidence curves. Source data

Quantifying the full activation functions of >7,500 receptor variants in a single experiment (Extended Data Fig. 8) revealed that expression is also a major influence on EC_50_. This has been previously predicted by theoretical^71–73^ models and agrees with recent work on plant hormone receptors^35^. After correcting for the influence of expression changes, EC_50_-modulating substitutions are topologically localized, including potency increasing variants in ICL3 and at both *N*-glycosylation sites in the unstructured N terminus. Substitutions that decrease potency are near the core activation network and, thus, likely modulate it. In contrast, residues increasing potency are far from the core activation network, likely affecting potency through different mechanisms. This distinction in the location and likely mechanism of LoF and GoF variants parallels observations in Src and other kinases, where substitutions that decrease kinase activity are enriched close to the active site but substitutions that increase it are not^74^.

Precision modalities such as biased signaling and allosteric modulation would allow the development of powerful new GPCR drugs, therapeutics with improved safety and the successful targeting of ‘undruggable’ GPCRs. Despite a revolution in GPCR structural biology, we lack sufficient functional understanding of GPCR activation and effector recruitment to design allosteric and biased modulators. We believe that quantifying multimodal mutational effects will accelerate the development of new precision drugs.

Residues biased toward Nb80 recruitment are enriched in ECL2, which forms part of the exosite of salmeterol, a β_2_AR agonist strongly biased toward G-protein signaling^57^, suggesting that mutant behavior is related to drug activity binding at the same sites. Likewise, known allosteric modulators bind in pockets with high impact substitutions that match their activities. AS408, an NAM, binds in a pocket with LoF and CoF residues, while Cmpd-6FA, a PAM, binds in a pocket with GoF and CoF residues. In addition to these known allosteric sites, GPCR-MAPS prioritizes an additional allosteric pocket to target in β_2_AR and deprioritizes one where substitutions only have limited effects.

GPCR drug discovery has proceeded from an era of blind high-throughput screening to an era of structure-based drug discovery. We believe that we are now entering a third era of GPCR drug discovery, where high-resolution functional maps of quantitative signaling parameters empower the rational design of advanced GPCR therapeutics, including precision biased and allosteric modulators.

## Methods

### Plasmids

Plasmids encoding SPARK2 components were gifts from C. Kim and A. Ting and were obtained from Addgene (plasmids 125228, 104839, 104840 and 104841). From these plasmids, we generated new plasmids using Gibson cloning. attB-HA-β_2_AR-eLOV-TEVcs-Gal4-UAS-Citrine encodes the receptor fusion followed by the bGH poly(A) signal, the UAS sequence and Citrine fluorescent protein. A 26-nt barcode was inserted in the 3′ UTR of the Citrine sequence. To make cloning more efficient, we transferred the fusion coding sequence from the NanoLuc-15-aa linker-β-arrestin2-HA-GS linker-TEVp plasmid from the pAAV vector (which contains G+C-rich inverted terminal repeats) to the pUC19 vector. We then used site directed mutagenesis to introduce the S153N variant to TEV, generating uTEV1Δ). We then made versions of this plasmid with Nb80 and mini-G_s_ sequence instead of β-arrestin. We also modified the mini-G_s_ vector so that uTEV1Δ was fused N-terminally (NanoLuc-15-aa linker-GS linker-TEVp-mini-G_s_).

pCAG-NLS-HA-Bxb1 was a gift from K. Matreyek and D. Fowler.

### Development of GPCR-MAPS recruitment assays

We started with the SPARK2 system^41^ and extensively reengineered the system to enable massively multiplexed experiments. First, we combined the GPCR construct and the reporter construct onto a single plasmid and added a DNA barcode (26 nt) in the 3′ UTR of the Citrine reporter so that the GPCR variant on any given plasmid can be identified (Fig. 1a and Extended Data Fig. 1e). We also added the attB recombination site to enable integration into HEK 293T landing pad cells^40^, which ensures a copy number of exactly 1 in each cell. We used the active-state-specific nanobody Nb80 (ref. ^42^) as the binding partner to optimize the assay. While the original assay used transient transfection to introduce transgenic material and took place over the course of minutes, we found that substantially increased times were necessary to obtain detectable signal, presumably because of the reduction in copy number of the receptor and reporter constructs (24 h; Extended Data Fig. 1a). Furthermore, we tested evolved variants of both the TEV and the LOV domain, uTEV1Δ (ref. ^75^) and hLOV1 (ref. ^76^); the combination that yielded the highest signal-to-noise ratio was uTEV1Δ and eLOV (Extended Data Fig. 1b). Next, we also validated the use of β-arrestin and mini-G_s_^77^ as the binding partner in this system (Extended Data Fig. 1b). Lastly, to account for differences in barcode abundance in the library, as well as barcode-specific effects on RNA abundance, we also included a condition in which Gal4 is expressed alone (Extended Data Fig. 1f); this condition should maximally stimulate all barcodes and provides normalization for the experimental conditions^27^.

### Saturation mutagenesis library creation

We designed six pools of mutation-bearing oligonucleotides, each pool representing mutated coding sequence for 66–70 residues of the full-length (413 residues) β_2_AR with a flanking wild-type sequence for amplification from the pool (using primers dialout_tile[1–6]_[F/R]; Supplementary Table 5). For each pool, we designed primers to amplify the whole plasmid except for the mutated segment (using primers b2AR_satmut_tile[1–6]_[F/R]) and then used Gibson assembly to introduce the mutagenic oligos. Oligos were designed to encode all possible single amino acid changes (using the human optimized codon for each alternative amino acid), all premature stop codons and all synonymous codons when possible (using the most optimal codon, excluding the wild-type codon).

### Barcode integration and variant–barcode association

First, 10 μg of the attB-HA-β_2_AR-eLOV-TEVcs-Gal4-UAS-Citrine saturation mutagenesis plasmid was digested with 40 U of PpuMI (New England Biolabs) overnight at 37 °C; then, in the morning, another 10 U of PpuMI was added for a 30-min incubation at 37 °C. Then, the cut band was gel-purified. The barcode construct (oTLM_257) was made double-stranded by PCR amplification with oTLM_258 and oTLM_259 (Supplementary Table 6). The double-stranded barcode was incorporated into the cut plasmid backbone by Gibson assembly and transformed into electrocompetent bacteria. Following recovery in SOC medium, we plated 0.1% of the transformation to estimate the number of clones and inoculated the rest into Luria–Bertani medium with ampicillin for overnight growth. The estimated number of clones was 300,000. In the morning, the bacteria were pelleted and the plasmid was midi-prepped (Qiagen plasmid plus midi kit).

We used PacBio long-read sequencing to associate each variant with the barcode. We digested the plasmid pool with BsiWI (New England Biolabs) to release the coding sequence and barcode and SMRT bell adaptors were ligated according to manufacturer’s instructions. The library was sequenced on a PacBio Sequel IIe. We used alignparse^78^ to align reads, quality filter and call sequences for each barcode (Extended Data Fig. 1).

### Landing pad integration

HEK 293T LLP-iCasp9-Blast^40^ cells were a gift from K. Matreyek. Cells were grown in DMEM with GlutaMAX (Gibco), with tetracycline-free FBS. A total of 14 million cells were plated onto a T225 flask. The next day, transfection was performed using Lipofectamine 3000 (Invitrogen) with 17.5 μg of pCAG-NLS-HA-Bxb1 and 17.5 μg of the mutated and barcoded attB-HA-β_2_AR-eLOV-TEVcs-Gal4-UAS-Citrine. The cells were allowed to incubate for 2 days. Then, the medium was removed and replaced with medium containing 2 μg ml^−1^ doxycycline before incubating for 1 day. Then, the medium was changed to medium containing 2 μg ml^−1^ doxycycline and 10 nM rimiducid (MedChemExpress) before incubating for 1 day. Then, the medium was changed to include just 2 μg ml^−1^ doxycycline and outgrowth of cells was allowed for ~1 week, passaging cells when they reached 90% confluency.

### GPCR-MAPS recruitment experiment

A total of 10 million cells were plated onto each T175 flask and allowed to incubate overnight. The next day, the cells were transfected with 36 μg of transducer plasmids (pUC19-NanoLuc-β-arrestin-uTEVp1 or pUC19-NanoLuc-Nb80-uTEVp1), using Lipofectamine 3000 and allowed to incubate overnight. The next morning, cells were treated with medium containing furimazine and either drug or DMSO (13.05 ml of DMEM, 126.56 μl of furimazine, 270 μl of 1 M HEPES and 13.5 μl of isoproterenol or DMSO]. Furimazine was obtained from the Nano-Glo live-cell assay system (Promega). Isoprenaline was obtained from Sigma (15627). Cells were incubated in the dark (covered with aluminum foil) at 37 °C for 24 h. Then, cells were lifted with trypsin–EDTA, pelleted and stored at −80 °C until RNA extraction. For the Gal4 maximum stimulation condition, after 10 million cells were plated and grown overnight, 36 μg of pUC19-CMV-Gal4 was transfected with Lipofectamine 3000. The cells were allowed to incubate for 30 h; then, cells were pelleted and stored at −80 °C.

### RNA isolation and sequencing library preparation

For the first set of experiments (Nb80 and β-arrestin recruitment with 10 μM isoprenaline), cell pellets were thawed and resuspended in buffer RLT with added β-mercaptoethanol. Then, they were homogenized by centrifuging through QIAshredder columns. RNA was then purified from the homogenate with RNeasy midi prep kit (Qiagen) and eluted with 400 μl of water. Between 120 and 160 μg of total RNA was used as input for reverse transcription (RT) reactions (6–8 μg per reaction, 20 reactions per sample) using SuperScript III reverse transcriptase (Invitrogen). For the concentration–response experiment, Dynabeads mRNA direct purification kit (Thermo Fisher Scientific) was used according to the manufacturer’s instructions and mRNA was eluted in 80 μl of Tris-HCl. Total mRNA yields were between 11.5 μg and 16 μg. Between 5.75 and 8 μg of mRNA was used as input for RT (1.15–1.6 μg per reaction, ten reactions per sample). For both sets of reactions, RT was carried out with 2 pmol of primer (oTLM_266; Supplementary Table 5) and incubated at 55 °C for 1 h followed by 75 °C for 15 min. Then, RNase A was added to a final concentration of 100 μg ml^−1^ and reactions were incubated at 37 °C for 30 min. Then, reactions were cleaned up with Nucleospin PCR cleanup columns (Macherey Nagel) and eluted in 50 μl of elution buffer. Next, 0.5 μl of the cDNA eluate was used in a qPCR reaction (Q5 high-fidelity polymerase, New England Biolabs; EvaGreen fluorescent dye, Biotium) to determine the number of cycles required to reach near-plateau phase. Then, 40 μl of cDNA was amplified across four 50-μl PCR reactions with the following program: 98 °C for 30 s, followed by 13–16 cycles of 98 °C for 15 s, 64 °C for 30 s and 72 °C for 30 s.

Reactions were column-purified and 4% of this was used as input for a second round of PCR, which added the rest of the Illumina adaptor and unique index sequences. The second round PCR was 98 °C for 30 s, followed by five cycles of 98 °C for 15 s, 64 °C for 30 s and 72 °C for 30 s. Frameshifting primers were used to introduce variability in the first few cycles of the sequencing reaction (oTLM_277–282; Supplementary Table 5). Reactions were then gel-purified and analyzed on TapeStation (Agilent Technologies). Sequencing was performed with Illumina NovaSeq with 2× 50-bp paired-end reads.

An additional step was taken for the concentration–response experiment. Following RT, we performed triplicate qPCR reactions with each cDNA sample, along with a set of serial dilutions of the original library plasmid, to estimate the relative quantity of Citrine RNA produced from each experiment. We used primers oTLM_021 and oTLM_024, which amplify the 5′ UTR of Citrine. We chose to amplify this region instead of the barcode (as performed for the library prep) to avoid idiosyncratic effects of amplification efficiency caused by diverse and potentially different barcode identities across different samples. Following qPCR, we fit a standard curve (Supplementary Fig. 5b) and then used this to estimate RNA abundance for the concentration–response samples (Fig. 5b).

### Surface expression experiment cell culture and FACS

Cells were cultured in cell culture flasks (245 × 245 mm) until ~80% confluent. Then, cells were lifted with trypsin–EDTA and pelleted; the supernatant was removed and resuspended in blocking buffer (1% BSA in PBS). Cells were counted and 50 million cells were transferred to a 5-ml conical tube with 3.5 ml of blocking buffer, which was incubated on ice, covered from light, with rocking for 30 min. Then, anti-HA antibody (Cell Signaling HA tag 6E2 Alexa Fluor 647 conjugate) was added at dilution of 1:100 and incubated on ice, covered from light, with rocking for 60 min. Then, cells were pelleted; the supernatant was removed and resuspended in blocking buffer. Sorting was performed on the BD FACSAria instrument. Bins were drawn so that each represented ~25% of cells. In total, ~20 million cells were sorted for replicate 1 and ~30 million cells were sorted for replicates 2 and 3. In total, 30–45 million 2× 50-bp reads were collected on an Illumina NovaSeq instrument for each replicate.

### Surface expression sequencing library preparation

Sorted cells were pelleted and DNA extracted using Qiagen DNeasy blood and tissue kit, using two columns for each sample. Then, 200 μl of elution buffer was used to elute from each column and eluates for each sample were combined. Next, 320 μl of each sample was was used as input for 16× 50-μl PCRs with primers adjacent to the barcode that include partial Illumina adaptors and varying lengths of degenerate bases to increase nucleotide complexity in the first few cycles of the sequencing run (oTLM_277–282; Supplementary Table 5). The protocol started at 98 °C for 30 s, followed by 25 cycles of 98 °C for 15 s, 64 °C for 30 s and 72 °C for 30 s. Reactions were column-purified and 4% of this was used as input for a second round of PCR, which added the rest of the Illumina adaptor and unique index sequences. The second round PCR was performed at 98 °C for 30 s, followed by five cycles of 98 °C for 15 s, 64 °C for 30 s and 72 °C for 30 s. The products of the second PCR were gel-purified and analyzed on TapeStation, before sequencing on an Illumina NovaSeq with 2× 50-bp paired-end reads.

### Sequencing data processing

Paired-end sequencing reads were first merged with vsearch^79^ and then adaptors were trimmed with cutadapt^80^. This left each read representing the barcode sequence, which was then matched with barcodes identified in the variant–barcode association with a custom script.

### Calculation of surface expression scores

Surface expression scores were calculated as previously described^25^. Briefly, the frequency of each variant in each bin was enumerated by combining counts of all barcodes associated with that variant. The frequency, along with the total number of cells sorted into each bin, was used to estimate the number of cells in each bin. Then, the log_10_-transformed geometric mean fluorescence value of all cells sorted into each bin was combined with the estimated cell counts to arrive at the raw surface expression estimate. These estimates were then normalized between 0 (represented by the median of presumed LoF variants; for example, premature stop codons before the 300th amino acid) and 1 (the wild-type score). Surface expression scores were considered high confidence if the estimated number of cells sorted was >50.

### Calculation of recruitment scores

First, barcode read counts for all barcodes representing the same variant were combined and then the frequency of that variant was calculated by dividing that number by the total number of barcode counts in the sample. Then, to arrive at a raw score, we divided the variant frequency in the experimental sample by the variant frequency in the Gal4 sample and took the log_2_ of this value. The Gal4 sample represented a maximum stimulation condition, in which the barcode frequency in the library and any idiosyncratic effects of the barcode sequence on RNA stability should be captured. Raw scores were then scaled between 0 and 1, with the median of nonsense substitutions set as 0 and the wild-type score set as 1. Recruitment scores were considered high confidence if the frequency of that variant in the Gal4 condition was >2 × 10^−5^.

### LOWESS modeling and identifying outlier positions

The python package statsmodels.nonparametric.smoothers_lowess^81^ was used with the following parameter values: frac = 0.5, it = 3 and delta = 0.0. Following curve fitting, residuals were calculated as the distance in the *y* axis between the data point and the line and *z* scores were calculated as the residual divided by the s.e.m. In the case of Nb80 versus β-arrestin, the average s.e.m. between the two measurements was used.

### Structural analysis

Residue contacts were defined by GetContacts (https://getcontacts.github.io/index.html). We used the definition of the isoprenaline-binding site as presented in the publication solving and describing the structure^44^. Specific interactions between residues and isoprenaline atoms were taken from the publication and GetContacts. The binding interface of β_2_AR with Nb80 was defined by GetContacts analysis of structure. The predicted inactive and active structures in Fig. 6k were taken from GPCRdb and were produced with AlphaFold-Multistate^45^. These structures were used to enable visualization of the entire ICL3, which is not resolved in experimentally solved structures.

### Concentration–response curve fitting

In the package neutcurve^82^, we used the neutcurve.hillcurve.HillCurve function to fit curves with the following options: infectivity_or_neutralized = ‘neutralized’, fix_slope_first = True, fs_stderr = None, fixbottom = False, fixtop = False, fixslope = False, fitlogc = False, use_stderr_for_fit = False and no_curve_fit_first = False. Following the fitting procedure, we then filtered curves on the basis of the following heuristics: *R*^2^ > 0.9, EC_50_ within the measured range (that is, between 10 pM and 10 μM) and *E*_max_ < 1.5× the maximum measured recruitment value. The number of variants filtered is presented in Supplementary Fig. 5e.

### Fpocket pocket prediction

We used PDB 2RH1 as the structural template for pocket prediction and used the Fpocket web server with default settings to predict pockets. We chose the two pockets to simply illustrate cases of having many or few annotated residues.

### Statistics and reproducibility

Statistical analyses were performed using SciPy, NumPy and pandas. The DNA library of β_2_AR variants was generated once and transformed into landing pad cells once. Three replicates of the surface expression, as well as Nb80 and β-arrestin maximum recruitment experiments, were performed. For the concentration–response experiment, each concentration was performed in a single replicate. No statistical method was used to predetermine sample sizes. The experiments were not randomized. The investigators were not blinded to allocation during experiments and outcome assessment.

### Reporting summary

Further information on research design is available in the Nature Portfolio Reporting Summary linked to this article.

## Online content

Any methods, additional references, Nature Portfolio reporting summaries, source data, extended data, supplementary information, acknowledgements, peer review information; details of author contributions and competing interests; and statements of data and code availability are available at https://doi.org/10.1038/s41594-026-01907-3.

## Supplementary information

Reporting Summary

Peer Review File

Supplementary Tables 1–5

## Source data

Source Data Fig. 1 Statistical source data.

Source Data Fig. 2 Statistical source data.

Source Data Fig. 3 Statistical source data.

Source Data Fig. 4 Statistical source data.

Source Data Fig. 5 Statistical source data.

Source Data Fig. 6 Statistical source data.

Source Data Extended Data Fig. 1 Statistical source data.

Source Data Extended Data Fig. 2 Statistical source data.

Source Data Extended Data Fig. 3 Statistical source data.

Source Data Extended Data Fig. 4 Statistical source data.

Source Data Extended Data Fig. 5 Statistical source data.

Source Data Extended Data Fig. 6 Statistical source data.

Source Data Extended Data Fig. 7 Statistical source data.

Source Data Extended Data Fig. 8 Statistical source data.

## Data Availability

Files needed to reproduce analyses can be found via Zenodo at https://doi.org/10.5281/zenodo.15363336 (ref. ^83^). Raw sequencing reads can be found on the Sequence Read Archive under BioProject PRJNA1260170. Data and materials can be obtained from the corresponding authors upon request. Source data are provided with this paper.

## Code availability

Custom code to reproduce analyses can be found on GitHub (https://github.com/lehner-lab/GPCR-MAPS).
