# Supplementary material for: GPCR-MAPS: high-resolution functional and allosteric mapping of G-protein-coupled receptor activation functions and bias
Source: Nat Struct Mol Biol. 2026 Nov 11;33(11):1718–32. doi: 10.1038/s41594-026-01907-3 (PMC13759325; doi:10.1038/s41594-026-01907-3)
Supplement: Supplementary file 1 [file 41594_2026_1907_MOESM1_ESM.pdf]

nature portfolio

Corresponding author(s): Ben Lehner
Taylor Mighell

Last updated by author(s): Aug 20, 2026

Reporting Summary

Nature Portfolio wishes to improve the reproducibility of the work that we publish. This form provides structure for consistency and transparency in reporting. For further information on Nature Portfolio policies, see our Editorial Policies and the Editorial Policy Checklist.

Statistics

For all statistical analyses, confirm that the following items are present in the figure legend, table legend, main text, or Methods section.

n/a	Confirmed	
☐	☒	The exact sample size (*n*) for each experimental group/condition, given as a discrete number and unit of measurement
☐	☒	A statement on whether measurements were taken from distinct samples or whether the same sample was measured repeatedly
☐	☒	The statistical test(s) used AND whether they are one- or two-sided<br>*Only common tests should be described solely by name; describe more complex techniques in the Methods section.*
☒	☐	A description of all covariates tested
☐	☒	A description of any assumptions or corrections, such as tests of normality and adjustment for multiple comparisons
☐	☒	A full description of the statistical parameters including central tendency (e.g. means) or other basic estimates (e.g. regression coefficient) AND variation (e.g. standard deviation) or associated estimates of uncertainty (e.g. confidence intervals)
☐	☒	For null hypothesis testing, the test statistic (e.g. *F*, *t*, *r*) with confidence intervals, effect sizes, degrees of freedom and *P* value noted<br>*Give P values as exact values whenever suitable.*
☒	☐	For Bayesian analysis, information on the choice of priors and Markov chain Monte Carlo settings
☒	☐	For hierarchical and complex designs, identification of the appropriate level for tests and full reporting of outcomes
☐	☒	Estimates of effect sizes (e.g. Cohen's *d*, Pearson's *r*), indicating how they were calculated

Our web collection on statistics for biologists contains articles on many of the points above.

Software and code

Policy information about availability of computer code

Data collection	FACSDiva software used onboard the FACSaria instrument.
Data analysis	ChimeraX version 1.6<br>FlowJo version 10.8.0<br>matplotlib version 3.9.4<br>numpy version 2.0.2<br>seaborn version 0.13.2<br>pandas version 2.2.3<br>scipy version 1.8.0<br>sklearn version 1.0.2<br>statsmodels version 0.14.4<br>neutcurve version 2.1.0

For manuscripts utilizing custom algorithms or software that are central to the research but not yet described in published literature, software must be made available to editors and reviewers. We strongly encourage code deposition in a community repository (e.g. GitHub). See the Nature Portfolio guidelines for submitting code & software for further information.

Data

Policy information about availability of data

All manuscripts must include a data availability statement. This statement should provide the following information, where applicable:

- Accession codes, unique identifiers, or web links for publicly available datasets
- A description of any restrictions on data availability
- For clinical datasets or third party data, please ensure that the statement adheres to our policy

Files needed to reproduce analyses can be found at zenodo (https://zenodo.org/records/15363336). Raw sequencing reads can be found at Sequence Read Archive (accession number PRJNA1260170).

Research involving human participants, their data, or biological material

Policy information about studies with human participants or human data. See also policy information about sex, gender (identity/presentation), and sexual orientation and race, ethnicity and racism.

Reporting on sex and gender	Our study does not use any sex or gender aspect.
Reporting on race, ethnicity, or other socially relevant groupings	Our study does not use any race, ethnicity, or other socially relevant groupings.
Population characteristics	This study does not use human research participants.
Recruitment	There were no participants in this study.
Ethics oversight	Not applicable.

Note that full information on the approval of the study protocol must also be provided in the manuscript.

Field-specific reporting

Please select the one below that is the best fit for your research. If you are not sure, read the appropriate sections before making your selection.

☒ Life sciences ☐ Behavioural & social sciences ☐ Ecological, evolutionary & environmental sciences

For a reference copy of the document with all sections, see nature.com/documents/nr-reporting-summary-flat.pdf

Life sciences study design

All studies must disclose on these points even when the disclosure is negative.

Sample size	We sought to include all single amino acid substitutions of b2AR in this study.
Data exclusions	Surface expression variant measurements in which the variant was estimated to be present in less than 50 cells was excluded. Variants with a frequency less than 2e-5 were excluded from recruitment analyses. Variants were filtered from the dose response analysis based on filters described in the manuscript.
Replication	All experimental conditions were replicated, and results were in agreement.
Randomization	This is not relevant- the same library of variants was used in all conditions.
Blinding	Blinding is not relevant to this experiment. All variants in all conditions are analyzed with the exact same procedure.

Reporting for specific materials, systems and methods

We require information from authors about some types of materials, experimental systems and methods used in many studies. Here, indicate whether each material, system or method listed is relevant to your study. If you are not sure if a list item applies to your research, read the appropriate section before selecting a response.

Materials & experimental systems

n/a	Involved in the study
☐	☒ Antibodies
☐	☒ Eukaryotic cell lines
☒	☐ Palaeontology and archaeology
☒	☐ Animals and other organisms
☒	☐ Clinical data
☒	☐ Dual use research of concern
☒	☐ Plants

Methods

n/a	Involved in the study
☒	☐ ChIP-seq
☐	☒ Flow cytometry
☒	☐ MRI-based neuroimaging

Antibodies

Antibodies used: HA-Tag (6E2) monoclonal antibody Alexa Fluor 647 Conjugate (#3444, Cell Signaling Technologies)

Validation: This is a widely used antibody for a non-species specific epitope. Validation on supplier's website.

Eukaryotic cell lines

Policy information about cell lines and Sex and Gender in Research

Cell line source(s): The cell line is a derivative of HEK293T; described in this publication: https://academic.oup.com/nar/article/48/1/e1/5587635

Authentication: The cell line was not authenticated.

Mycoplasma contamination: The cell line tested negative for mycoplasma.

Commonly misidentified lines (See ICLAC register): None.

Plants

Seed stocks: We did not use seed stocks.

Novel plant genotypes: We did not use any plants.

Authentication: We did not use any plants.

Flow Cytometry

Plots

Confirm that:

☒ The axis labels state the marker and fluorochrome used (e.g. CD4-FITC).

☒ The axis scales are clearly visible. Include numbers along axes only for bottom left plot of group (a 'group' is an analysis of identical markers).

☒ All plots are contour plots with outliers or pseudocolor plots.

☒ A numerical value for number of cells or percentage (with statistics) is provided.

Methodology

Sample preparation: To dissociate cells, they were first washed once with PBS, then were incubated with Trypsin-EDTA (0.05%) for 4 minutes at room temperature. Then cells were washed off the plate with media, then pelleted and resuspended in blocking buffer (1% bovine serum albumin in phosphate buffered saline). Cells were counted and 30-50M cells were transferred to a new tube. Blocking buffer was added to attain 15M cells/mL. Then, cells were incubated on a rotating wheel at 4° for 30 minutes. Following this, HA-Tag (6E2) monoclonal antibody Alexa Fluor 647 Conjugate (#3444, Cell Signaling Technologies) was added to a final concentration of 1:100, then cells were again incubated on a rotating wheel at 4° for an additional 60 minutes. At

	this point, cells were pelleted and supernatant removed, then resuspended in 5 mL of blocking buffer with propidium iodide (1 µg/mL).
Instrument	Cells were sorted on a BD FACSaria II.
Software	onboard FACSDiva software was used to analyze data. Flowjo was used to visualize data.
Cell population abundance	After the gating for recombined and alive cells, all cells were sorted and collected.
Gating strategy	Cells were first filtered by forward scattering area and side scattering area, then single cells were isolated with forward scattering width and height. BFP positive cells were filtered as unrecombined landing pad cells, and propidium iodide-positive cells were filtered as dead cells. Then, the remaining population of cells was sorted into four bins, based on Alexa Fluor 647 signal intensity, that were designed to result in a similar number of cells in each bin.

☒ Tick this box to confirm that a figure exemplifying the gating strategy is provided in the Supplementary Information.
