# Supplementary material for: GPCR-MAPS: high-resolution functional and allosteric mapping of G-protein-coupled receptor activation functions and bias
Source: Nat Struct Mol Biol. 2026 Nov 11;33(11):1718–32. doi: 10.1038/s41594-026-01907-3 (PMC13759325; doi:10.1038/s41594-026-01907-3)
Supplement: Supplementary file 2 [file 41594_2026_1907_MOESM2_ESM.pdf]

nature portfolio

Peer Review File

GPCR-MAPS: high-resolution functional and allosteric mapping of G protein-coupled receptor activation functions and bias

Corresponding Author: Professor Ben Lehner

A version of this paper was originally rejected for publication by Nature Structural & Molecular Biology, however that decision was reconsidered after appeal by the authors.

Version 0:

Decision Letter:

Nature Structural & Molecular Biology NSMB-A51389

2nd Sep 2025

Dear Dr. Lehner,

Thank you for submitting your manuscript, "A complete functional map of a G protein-coupled receptor". Please accept my apologies for the delay in this decision. Nevertheless, the comments of 3 expert referees are below. You will see that while the reviewers express some support for the manuscript, they also list concerns which will prevent us from further consideration of the manuscript at Nature Structural & Molecular Biology

Specifically, they list concern regarding the novelty of the approach, its application to membrane proteins, as well as the advance in the biological insights into biology of β_2AR. Based on these comments, I am afraid we cannot offer to publish the study in Nature Structural & Molecular Biology. We hope the referees' comments will be useful to you in revising the manuscript for submission elsewhere.

However, considering the strength of the study, we consulted our editorial colleagues at EMBO Journal and Nature Communications. Both of the journals are interested in the study and invite you to transfer to their journal for further consideration. Please see below for details.

>>While we cannot continue with this manuscript at our journal, we have discussed the study with our colleagues at The EMBO Journal. As you may know, The EMBO Journal is a non-profit, fully open-access journal published by EMBO Press and offers rapid and transparent peer review with a single major revision round policy. The handling editor Ieva Gailite would be happy to consider your manuscript for publication at The EMBO Journal after its revision in response to the reviewers' comments. If you are interested in this option, please submit the revised manuscript to The EMBO Journal via the transfer link below. Upon transfer, Ieva will contact the original reviewers for a rapid assessment of your revised manuscript. Please feel free to contact Ieva at i.gailite@embojournal.org if you have any questions about the transfer, The EMBO Journal or its policies.

>>Although we regret that we cannot offer to publish your paper, I have discussed your manuscript and the reviewers' comments with our colleagues at Nature Communications. They will send an appropriately revised version out for further review if you transfer the revised manuscript to Nature Communications. Should you wish to have your revised paper considered by Nature Communications, please use the link to the Springer Nature manuscript transfer service in the footnote once the revision is ready, and include a point-by-point response to the reviewers' concerns.

Your handling editor at Nature Communications would be Dr Sophie McKenna (sophie.mckenna@nature.com). If there is anything you would like to discuss before transferring the paper, please don't hesitate to contact her by e-mail. Please note

that Nature Communications is a fully open access journal. For information about article processing charges, open access funding, and advice and support from Nature Portfolio, you may consult the Nature Communications Open Access page.

I am sincerely sorry we could not be more positive on this occasion.

Sincerely,
Kat

Katarzyna Ciazynska, PhD
(she/her)
Senior Editor
Nature Structural & Molecular Biology
https://orcid.org/0000-0002-9899-2428

Reviewer #1 (Remarks to the Author):

In "A complete functional map of a G-protein coupled receptor", Mighell and Lehner describe a massive high-throughput screen of single point variants of a GPCR, with readout including effects on two downstream effectors and variation of ligand concentrations. It's an experimental tour de force leading to a massive dataset.

The assay, newly developed for this work, aims at streamlined detection and is reduced to the essentials (protein-protein interaction rather than full endogenous signalling pathways). For molecular details, this is a good idea, though the flipside likely is that this also reduces the findings to direct molecular consequences, and potential buffering or other regulatory elements are stripped away. For a detailed molecular map, this may be considered an advantage, in practice though this may also highlight regions that aren't as promising in the endogenous system. The authors should discuss this in the manuscript.

It is worth noting that the authors are not the first to study GPCRs in a MAVE fashion, and while several previous articles are cited, there is no direct comparison to prior results. These should be added (with visualisations like scatter plots at least in supplementary) and discussed.

In addition to the classic loss of functon(LoF) category, the authors separate other non-wt-like variants into Gain-of-functon(GoF), where all substitutions lead to increased signalling (or at least, increased readout) and Change-of-functon(CoF), where there are differential effects based on the nature of the amino acid that is substituted in. This "new" category is perhaps the most interesting from a fundamental protein biochemistry perspective, and it may be worth diving a bit more into which amino acids have which consequences, similar to Fig. 4j.

Additional comments:

Fig. 2: How do you explain the variants that score 0 for surface expression, but above zero for Nb80 and beta-arrestin signalling?
P13, the observation about GoF residues being dispersed throughout the structure while LoF residues are clustered appears in contrast to (Gerasimavicius et al. 2025) - any thoughts on that?
P13: a reader from outside the GPCR field won't be familiar with all the motif designations; only one is mentioned in the introduction. If they are relevant for interpretation, please introduce and ideally also illustrate all.
Fig. 5d, 1.5nM looks like total loss of signalling. The wild-type tiles are still indicated in white. Does that mean the wt sequence is still functional "as normal"? How about
Fig. 5f-i, how are the different categories defined?
P20, "there is substantial overlap" does not match the 4/29 overlapping GoF positions, please either rephrase or explain how this is to be read. Even 23/59 overlapping LoF is borderline for my interpretation of "substantial".
I don't see references to Figs 6f/g/h in the text until a few pages later than the rest of this Figure. It seems surprising that the LoF case has high surface expression for all but one variant, while the GoF variant has low expression and low EC50. Please elaborate on the proposed molecular mechanism.
Fig. 6, some labels say EC50, others log EC50, yet the numerical ranges are the same. Presumably the log got lost in some.
P21, "mutations in the second shell are likelier to reduce affinity than to increase it", is that corrected for the general background distribution of mutational effects?
The section on pockets and target mechanisms is much shorter than one would expect from the abstract and introduction.

Reviewer #2 (Remarks to the Author):

Mighell and Lehner investigate how sequence variation in a G protein–coupled receptor influences function, applying deep mutational scanning (DMS) to characterize mutational effects on receptor signaling. Their premise is that a "functional map" can reveal fundamental features of GPCR function, identify potential allosteric sites for drug discovery, and ultimately guide

precision GPCR therapeutics. As a model, they use the β_2-adrenergic receptor, a well-characterized GPCR with extensive prior structural and mutagenesis data. This work builds on several recent studies that have applied saturation DMS or comprehensive scanning approaches to GPCRs. The authors introduce some methodological innovations, notably a large-scale DMS at 12 agonist concentrations to assess mutational effects on dose–response relationships.

The technical execution is strong, but in several places the novelty of the conclusions is limited because many findings recapitulate known mutational effects from earlier β_2AR studies. In particular, while the authors identify some new functionally important residues, these are not validated prospectively, and several broader claims about the impact of DMS on drug discovery are not fully substantiated. Overall, I think the manuscript is likely to be of interest to NSMB readers as a great example of the power of DMS to understand membrane protein function, but could be strengthened by the points outlined below.

Major points:
1) The authors point out that prior DMS studies are limited by their use of signaling systems because these have non-linearities and feedback that influence the data. Instead the authors set up a system to measure protein recruitment directly, using an active-state specific nanobody (Nb80) and beta-arrestin as proxies for function. The rationale for using Nb80 and β-arrestin recruitment as readouts is sound, especially in avoiding non-linearities and feedback seen in transcriptional signaling assays. However, the manuscript equates Nb80 recruitment with "G protein signaling" or "Nb80 signaling" and β-arrestin recruitment with "arrestin signaling," and interprets mutation effects as "Nb80-biased" or "arrestin-biased." This framing misleads readers, since Nb80 recruitment does not necessarily share the same allosteric requirements as G protein activation. The authors could strengthen their case by emphasizing the advantages of the recruitment assays (e.g., reduced signal amplification) rather than implying direct equivalence to canonical signaling pathways.

2) The system the authors have set up is quite complex - I think readers would benefit from understanding why such a complex system is necessary (e.g. why not just remove the whole LOV-luciferase system. It's could intuit based on our own experience in the GPCR field, but likely will not be apparent to all. I think it would also be very high yield for the authors to move the wild-type dose response curve for isoproterenol shown in Fig 5f (ideally with replicates) to the first figure to highlight the robustness of the system. Since the authors want to connect signaling to their assay, the manuscript could be improved by comparing the EC50 for iso in the transcriptional assay to a cAMP assay.

3) The DMS identifies interesting functional residues, such as N-terminal glycosylation sites and C-terminal positions affecting signaling. These could be the most novel findings of the work. Even limited prospective validation (for example, testing a subset of mutations in a simple cAMP assay) would greatly enhance confidence that the approach can generate actionable predictions, not just retrospective rationalizations.

4) The functional meaning of constitutively active (COF) mutations is unclear. For example, for S120, can the authors rationalize which substitutions yield gain- vs. loss-of-function? Additional targeted assays, even for a few sites, would clarify how predictive the DMS is for COF phenotypes.

5) For the Nb80 vs arrestin comparison - a major reason for this difference could come down to mutations that influence receptor expression levels. Did the authors account for surface expression when making this comparison? For example, it is known that W99 is an important residue for GPCR folding - is the "Nb80 bias" simply because mutations at this residue lead to less receptor expression and it is easier to see Nb80 recruitment at low expression levels compared to arrestin recruitment?

6) The dose response curve work is heroic and I applaud the authors for this. But at some level it's a bit less impactful if the major conclusions could have been predicted by an alanine scan (though it seems that the majority of the correlation in Fig. 6b is driven by a small subset of LOF sites). I think perhaps some of the most interesting results here are the COF sites. The authors point out F282 as an interesting COF site, which it does seem to be, e.g. even very lowly expressed mutations at this position seem to have high iso potency. This would be an example of a fascinating new observation to dig deeper - given the important structural changes known to happen at this site, can they at least rationalize with the mutations observed?

7) Figure 7 is challenging to interpret due to color schemes and unclear selection thresholds. Showing the entire receptor surface, with consistent coloring and explicit criteria for site selection, would help assess whether identified sites are truly discrete and novel.

Minor points:
1) Lines 22-24: the majority of GPCR drug discovery failures are not because molecules target the orthosteric pocket. In general, orthosteric targeting has been incredibly fruitful for GPCR drug discovery and there are many orthosteric molecules that have entered Phase2 and Phase 3 trials. The vast majority of failures are because we simply don't understand the human biology well enough or the preclinical models were not predictive.

2) Line 54 - for many GPCRs, phosphorylation is not just limited to the C tail; often can happen in other intracellular loops.

3) Figure 4e/f legend is flipped

4) In the volcano plots, it would be helpful to have labels for the residues that are called out in the figure - otherwise it's a bit unclear how well these scored relative to the other sites

Reviewer #3 (Remarks to the Author):

In their manuscript, Mighell and Lehner describe the generation of functional maps of GPCRs covering the complete coding sequence of a receptor. They measured surface expression of and arrestin and nanobody 80 (Nb80) interaction for a DMS library of mutants of the beta-2-adrenergic receptor. Residues were then classified as loss-of-functon(LoF), gain-of-functon(GoF), and change-of-functon(CoF) where the function differed clearly from the expected function at the given expression level. The LoF, GoF and CoF positions were then analysed in the context of GPCR structures, the LoF residues were used to define a ‘core activation network’. The authors identified residues with a higher functional impact on either arrestin or Nb80 and analysed their positions in structures of the beta-2-adrenergic receptor, where they identified ECL2 as a hotspot for biased agonism. Finally, they rationalised the binding sites of allosteric modulators in context of the whole data set.

The authors have developed a new method termed GPCR-MAPS, which allows direct quantification of functional consequences of mutations in GPCRs for saturation mutagenesis libraries. This is a major advancement over the previously used transcriptional reporters far downstream of direct GPCR signalling. In addition, twelve concentrations of ligand were used, which is an improvement over the four concentrations used in previous GPCR-DMS experiments (ref 26).

Instead of signalling, protein-protein interaction was quantified. While this certainly helps with comparisons of amplified G protein signalling pathways and non-amplified β-arrestin recruitment, it should still be made clearer that interaction rather than signalling was measured. In addition, Nb80 serves as a surrogate for G protein activation in this work. While Nb80 binding stabilises an active receptor state, it is not signalling competent. Nb80 and any G protein are very different proteins, which also differ in their binding mode and interactions, as can be seen in a comparison of the respective complex structures (3P0G and 3SN6). Nb80 binding could possibly be used as a surrogate for receptor activation potential, but not as a surrogate for G protein activation. Here, heterotrimeric G protein or, as mentioned, at least mini-Gs should be used to evaluate the functional consequences of all mutations to justify the claim that ‘signaling through both canonical pathways’ was tested.

As expected, transmembrane helices were more sensitive to mutations compared to loops. Losses of function were stronger than gains of function, which also meets expectations. All ligand-binding site residues were identified as having a strong impact on function, this serves as a validation of the results. It is surprising, though, that concentration-response curves could not be fitted for the vast majority of mutations in the ligand-binding site.
Here, the authors should ideally compare their results with results from a previous DMS paper on beta-2-adrenergic receptor (ref 26) and the alanine-scan based results (ref 18). Do the results differ, and if yes, how? What is the threshold at which concentration-response curves can still be fitted?

It is not quite as striking (cf. line 295) that the majority of loss-of-function residues form an activation network connecting ligand- and transducer binding sites, as this was shown before, for GPCRs in general and for the beta-2-adrenergic receptor. Here, the authors could compare their network to previously determined networks, including general GPCR activation networks (Zhou et al., 10.7554/eLife.50279) and highlight the differences and similarities to previously identified networks.

Possibly the most novel of the findings in this manuscript is the identification of biasing residues, as this has not been reported for the beta-2-adrenergic receptor before at this scale.

The known allosteric modulator binding sites were analysed in the alanine scan paper on beta-2-adrenergic receptor (ref 18). Were the findings in this study different from the findings in the previous study, and if yes, how?

The predicted active pocket (Fig. 7) almost perfectly matches Orphan Site 5 identified in a paper by Hedderich et al. (10.1038/s41467-022-29609-6).

Overall, the figures would benefit from additional explanations and clarifications for readers from the GPCR field who are not DMS experts.

Comments:
Line 30: please be more specific about 7,500 proteins
Line 30 and throughout the manuscript: please replace ‘dose-response curve’ with ‘concentration-response curve’
Lines 89-98: The authors mention that ‘these systems have only been established for G protein mediated signaling pathways’ but fail to mention that two of the three papers using alanine scanning and signalling assays have employed β-arrestin recruitment assays.
Line 98-99: The paper by Jones et al. (10.7554/eLife.54895) at least tested four concentrations of ligand against 7800 mutations of the beta2-adrenergic receptor. Is the claim ‘Finally, these multiplexed studies were not extended to formal dose-response analysis’ down to whether or not authors of previous papers have fitted curves for each mutant?
Lines 106-109 and 212-213: Were all variants indeed tested for G protein and arrestin pathways? Lines 212-213 suggest that instead of G protein, Nb80 was used as a surrogate. While binding of Gs and Nb80, respectively, stabilises an active state of the receptor, the binding modes and interactions in the binding site differ. If Nb80 was tested rather than mini-Gs, please adapt the text accordingly.
Line 119: **of** all receptor variants

Line 161: ‘we found that substantially increased times were necessary’ – for what? Please clarify.
Line 201-203: Could you please comment on receptor variants that increase cell-surface expression compared to wild type?
Line 227: is it possible to identify mutations with increased signalling compared to wild type?
Line 276: Please speifiy what you mean by ‘highly variable effects on signaling’? Are these positions where the effect of introducing a mutation depends on the nature of the mutation?
Line 360: Could you please provide examples of concentration-response curves for at least some biased residues for readers to gauge the strength of the bias?
Line 361: please define ‘bias switch’
Lines 369-371: please list the residues which make up the β-arrestin binding interface. If these are listed somewhere, the reference is missing
Lines 444-446: While the data presented in this manuscript increases the available data >10 fold compared to alanine scans of β2AR, CB2R and AT1R, the β2AR deep mutational scanning paper by Jones et al. is not cited or taken into account in this calculation (https://doi.org/10.7554/eLife.54895). Of course, the earlier deep scanning study only tested four concentrations of ligand compared to the twelve concentrations in this study.
Line 454: Could you explain why the slope is 0.84, where one might expect a slope of 1?
Lines 455-458: Which variants were filtered and how were they treated in the analysis? These variants may be quite important
Lines 465-466: please provide the correlation of Nb80 and arrestin data as a comparison.
Throughout the manuscript: please use GPCRdb numbers in addition to residue numbers to facilitate comparison.
Supplementary data: please add binding site annotations (for ligand, Nb80, Gs, arrestin, allosteric modulators) to the data table S1.
Lines 489-490: How does the effect of mutations in the ligand-binding site in this work compare to the alanine scan data for the binding site?

Figure 1 (and elsewhere): Nb80 does not signal, therefore I would strongly recommend replacing ‘Nb80 signaling’ with a more appropriate term. Please include GPCRdb numbers in detail figures (e.g. 1 j, k, l) and label the scales directly.

Figure 6: a) The unit of the concentration is missing. b) Comparison of logEC50s: could the fitted line be added? When comparing logEC50s, did the authors take into account that different ligands (adrenaline and isoprenaline, respectively) were used in the two studies? In addition, please change the axis titles to ‘logEC50 […]’, as the displayed values do correspond to logEC50s, not EC50s. c) It is not clear whether EC50 and Emax values of Nb80 or arrestin or both are compared here. Same for d). e) would benefit from additional explanation and/or annotation.

Figure 7: This figure would benefit from additional annotation of the colour scales. Which mutations bias towards arrestin and Nb80? Why do the scales range from -1 to 1 rather than 0 to 1?

Figure 8: It would be interesting to show the components of the network here, purple lines alone are not very informative. Overall, the figure should be designed in such a way that the major findings can be obtained without reading the manuscript in detail. At present, this figure does not improve my understanding of the results.

** As a service to authors, Springer Nature Limited provides authors with the ability to transfer a manuscript that one journal cannot offer to publish to another journal, without the author having to upload the manuscript data again. To transfer your manuscript to another NPG journal using this service, please click on Link Redacted

** For Springer Nature Limited general information and news for authors, see http://npg.nature.com/authors.

Version 1:

Decision Letter:

Our ref: NSMB-A51389A-Z

14th Jul 2026

Dear Dr. Lehner,

Thank you for submitting your revised manuscript "GPCR-MAPS: high-resolution functional and allosteric mapping of G protein-coupled receptor activation functions and bias" (NSMB-A51389A-Z). It has now been seen by the original referees and their comments are below. Unfortunately, Reviewer #1 was unavailable to re-review the manuscript. We asked Reviewer #2 to assess your responses to comments of Reviewer #1, and they did not have any further concerns to raise. The reviewers find that the paper has improved in revision, and therefore we'll be happy in principle to publish it in Nature Structural & Molecular Biology, pending minor revisions to satisfy the referees' final requests and to comply with our editorial and formatting guidelines.

We are now performing detailed checks on your paper and will send you a checklist detailing our editorial and formatting requirements in a couple of weeks. Please do not upload the final materials and make any revisions until you receive this

additional information from us.

Thank you again for your interest in Nature Structural & Molecular Biology Please do not hesitate to contact me if you have any questions.

Sincerely,

Katarzyna Ciazynska, PhD
(she/her)
Senior Editor
Nature Structural & Molecular Biology
https://orcid.org/0000-0002-9899-2428

Reviewer #2 (Remarks to the Author):

The authors have answered my concerns appopriately. This work is ready for publication from my perspective.

Reviewer #3 (Remarks to the Author):

Many thanks to the authors for making substantial improvements on their manuscript.
I only have minor comments:

I previously commented that '7,500 proteins' is confusing. The authors have changed this wording in the original position (line 30, first submission), but re-introduced a similar phrase in the new manuscript (line 239, '>7,500 different receptors' and line 353, 'for thousands of different receptors'). Precise wording would help the reader in all of these cases.

Line 738 ff.: The presented dataset does not reveal the importance of the extracellular vestibule for G-protein biased activation. This would only be the case if all positions were tested in a G protein assay, not a Nb80-based assay. Please change the wording.

INTERNAL USE ONLY AIP modules required: ["Life Science","Chemistry"]

Open Access This Peer Review File is licensed under a Creative Commons Attribution 4.0 International License, which permits use, sharing, adaptation, distribution and reproduction in any medium or format, as long as you give appropriate credit to the original author(s) and the source, provide a link to the Creative Commons license, and indicate if changes were

made.
In cases where reviewers are anonymous, credit should be given to 'Anonymous Referee' and the source.
The images or other third party material in this Peer Review File are included in the article’s Creative Commons license, unless indicated otherwise in a credit line to the material. If material is not included in the article’s Creative Commons license and your intended use is not permitted by statutory regulation or exceeds the permitted use, you will need to obtain permission directly from the copyright holder.
To view a copy of this license, visit https://creativecommons.org/licenses/by/4.0/

Reviewer #1 (Remarks to the Author):

In "A complete functional map of a G-protein coupled receptor", Mighell and Lehner describe a massive high-throughput screen of single point variants of a GPCR, with readout including effects on two downstream effectors and variation of ligand concentrations. It's an experimental tour de force leading to a massive dataset.

We thank the referee for their enthusiasm and very constructive suggestions.

The assay, newly developed for this work, aims at streamlined detection and is reduced to the essentials (protein-protein interaction rather than full endogenous signalling pathways). For molecular details, this is a good idea, though the flipside likely is that this also reduces the findings to direct molecular consequences, and potential buffering or other regulatory elements are stripped away. For a detailed molecular map, this may be considered an advantage, in practice though this may also highlight regions that aren't as promising in the endogenous system. The authors should discuss this in the manuscript.

We agree with this critique and have made updates to the text to clarify these points:

Results, "GPCR-MAPS: multiplexed assay with pathway specificity" section, paragraph 1:

> *Our goal is to read out receptor states as directly as possible. In contrast to other approaches[27–30], we therefore focused on quantifying direct binding interactions with receptors. Quantifying direct binding has proven powerful for understanding signaling by soluble proteins[35] and GPCRs[36] and avoids the confounding influence of non-linearities, feedback, and differential signal amplification when quantifying activity downstream of endogenous signaling networks.*

It is worth noting that the authors are not the first to study GPCRs in a MAVE fashion, and while several previous articles are cited, there is no direct comparison to prior results. These should be added (with visualisations like scatter plots at least in supplementary) and discussed.

Thanks for this suggestion. We have added now scatterplot or hexbin plots comparing our recruitment scores with previous alanine scanning surface expression data (Heydenreich 2023) and deep mutational scanning (Jones 2020) in supplementary figure 1i-k. We made corresponding additions to text:

Results, "β2AR surface expression" section, paragraph 2:

> *Our surface expression estimates are in line with previously estimated surface expression levels of alanine substitutions[18] (Spearman's ρ = 0.43, Supplementary Figure 1i).*

Results, "β-arrestin and Nb80 recruitment" section, paragraph 2:

> *The Nb80 and β-arrestin recruitment scores are also correlated with each other (Spearman's ρ = 0.87), as well as with previous multiplexed measurements of β2AR variants signaling through the G_s pathway[27] (Spearman's ρ = 0.44 and 0.47 for Nb80 and β-arrestin recruitment, respectively, Supplementary Figure 1j-k).*

In addition to the classic loss of functon(LoF) category, the authors separate other non-wt-like variants into Gain-of-functon(GoF), where all substitutions lead to increased signalling (or at least, increased readout) and Change-of-functon(CoF), where there are differential effects based on the nature of the amino acid that is substituted in. This "new" category is perhaps the most interesting from a fundamental protein biochemistry perspective, and it may be worth diving a bit more into which amino acids have which consequences, similar to Fig. 4j.

Thanks for this suggestion. We have now made changes to the figures and text to highlight how different amino acids have different functional effects. Specifically, we have added amino acid identity annotations to the plots in Figure 2k, 3d, 3e, and 6h. We also made corresponding text updates:

Results, “Complete functional maps of Nb80 and β-arrestin recruitment” section, paragraph 1:

> *An example of a strong CoF residue is L75^{2x46} (Figure 2k), which is near the allosteric sodium binding site and neighbors LoF residue A76^{2x47}. At this position, mutations to small, uncharged residues (G, S, T, A, C) decrease surface expression, but increase Nb80 and β-arrestin recruitment relative to the modeled expectation. On the other hand, positively charged residues (H, R, K) are well expressed but strongly decrease recruitment of both proteins.*

Results, “Loss-of-function residues define a core activation network” section, paragraph 3:

> *Interestingly, of the six residues shared by the other two networks that are not part of our core activation network, we identify four of these as either CoF (S120^{3x39} and F332^{8x50}) or GoF (L124^{3x43} and D130^{3x43}), emphasizing the value of empirically assaying all 19 substitutions per position. At S120^{3x39}, which neighbors I121^{3x40} of the PIF motif, positively charged substitutions (R, K, H) yield stable receptors with significantly reduced recruitment, while the E and Q also yield stable receptors but significantly increase recruitment (Figure 3d). At L124^{3x43}, most variants decrease surface expression but increase relative recruitment, with the exception of the aromatic amino acids (W, F, and Y) and proline which recruit Nb80 less than expected given their expression level (Figure 3e).*

Results, “Potency tuning”, paragraph 6:

> *Changing the F to other aromatics (Y or W) at position 282 yield stable receptors with strongly reduced potency, suggesting these mutations could stabilize the inactive state of the receptor (Figure 6h). Other mutations change expression-adjusted potency in either direction, suggesting idiosyncratic effects on the active and/or inactive conformations.*

Additional comments:

Fig. 2: How do you explain the variants that score 0 for surface expression, but above zero for Nb80 and beta-arrestin signalling?

We believe that the surface expression assay and signaling (recruitment) assay, as implemented in this case, have different dynamic ranges. In particular, we think that the surface expression signal is compressed at the low end, so that variants with small amount of expression are indistinguishable from those with 0 expression.

P13, the observation about GoF residues being dispersed throughout the structure while LoF residues are clustered appears in contrast to (Gerasimavicius et al. 2025) - any thoughts on that?

We think these findings are not necessarily in conflict. First, our definitions of GoF/LoF are different- Gerasimavicius et al. are using human disease classifications, while we are using direct molecular measurements.

An important distinction here is that when we call GoF/LoF, we are operating on recruitment levels already corrected for abundance level. In other words, a variant that is moderately destabilized but with greater recruitment than modeled expectation will be called GoF by us but may not actually have a GoF phenotype in human cells/tissues.

Another important distinction is that our threshold for calling GoF is different from theirs: our position-based statistical test, followed by FDR correction, may be a more lenient threshold than their threshold of causing detectable human disease.

Finally, Gerasimavicius et al. do find that GoF are enriched on the surface of proteins (compared to core) which is consistent with what we find.

P13: a reader from outside the GPCR field won’t be familiar with all the motif designations; only one is mentioned in the introduction. If they are relevant for interpretation, please introduce and ideally also illustrate all.

Thanks for this suggestion. We have added text:
Introduction, paragraph 4:

> *This has provided important mechanistic insights, including descriptions of conserved "microswitch" motifs, such as the CWxP, DRY, PIF, and NPxxY motifs, which are sites where local structural rearrangements occur upon GPCR activation[16]. However, these structural descriptions are only the starting point for assigning a functional role for the hundreds of individual amino acids in each highly dynamic receptor.*

Fig. 5d, 1.5nM looks like total loss of signalling. The wild-type tiles are still indicated in white. Does that mean the wt sequence is still functional "as normal"? How about

In Figure 5d the white tiles are actually missing data. Wild-type-like variants are colored in gray.

Fig. 5f-i, how are the different categories defined?

All of the data was subjected to hierarchical clustering, the result of which is shown in Figure 5e. Then the members of the three largest clades are plotted together, and labeled according to their apparent phenotype, in 5f-i.

P20, "there is substantial overlap" does not match the 4/29 overlapping GoF positions, please either rephrase or explain how this is to be read. Even 23/59 overlapping LoF is borderline for my interpretation of "substantial".

We agree that calling this "substantial" is unsubstantiated.
We have updated this to read:

Results, "Potency tuning" section, paragraph 1:

> *There is overlap between these sets and the sets identified from the saturating concentration experiment (23/59 LoF shared, 4/29 GoF shared).*

I don't see references to Figs 6f/g/h in the text until a few pages later than the rest of this Figure. It seems surprising that the LoF case has high surface expression for all but one variant, while the GoF variant has low expression and low EC50. Please elaborate on the proposed molecular mechanism.

Thanks, we have added figure callouts to the proper spots.

Based on our scheme to normalize recruitment by abundance, we don't consider it surprising to have well expressed LoF or relatively poorly expressed GoF. By definition, these are positions where mutations have different functional effects compared to the expectation based on abundance, regardless of the abundance value.

Fig. 6, some labels say EC50, others log EC50, yet the numerical ranges are the same. Presumably the log got lost in some.

Thanks for spotting this. We have updated to logEC50 in Figure 6b and c. We do keep EC50 in panels i and j as here we are using the term to denote a category (EC50 loss of function, EC50 gain of function, etc).

P21, "mutations in the second shell are likelier to reduce affinity than to increase it", is that corrected for the general background distribution of mutational effects?

This statement is based on the fact that there are many more LoF residues in the second shell, and LoF are enriched as a class in the second shell (OR=4.12, $p=1.9 \times 10^{-4}$) while there is only one GoF residue in the second shell and GoF are not enriched as a class in the second shell.

The section on pockets and target mechanisms is much shorter than one would expect from the abstract and introduction.

Based on this and other critiques, we decided to re-write the introduction to give a more holistic view of the value of our method beyond existing approaches, as well as the data and analyses we generated:

Introduction:

G-protein coupled receptors (GPCRs) are the largest family of transmembrane proteins that receive and transmit signals from the outside to the inside of cells. These signals are very diverse and include neurotransmitters, metabolites, immune system molecules, pH, and photons of light[1]. The topology of GPCRs is highly conserved and consists of seven transmembrane (TM) helices connected by alternating intra- and extracellular loops (ECLs and ICLs). Binding of an agonist to the extracellular portion of the receptor results in an allosteric conformational change to the intracellular portion of the receptor[2], which enables binding and activation of one or more members of the family of heterotrimeric G-proteins, which subsequently activate diverse downstream signaling pathways[3]. Receptor activation also results in phosphorylation of the receptor, including in the C-terminal tail, and recruitment of β-arrestin, which terminates G-protein signaling by internalizing the receptor, and also scaffolds G-protein independent signaling[4]. Because of their role as master signal transducers and their availability on the cell surface, GPCRs are the most important class of drug targets, with one third of current clinical therapeutics targeting GPCRs[5,6].

The vast majority of established GPCR drugs act by binding to the orthosteric site and activating or inactivating the receptor[7]. However, alternative modalities for modulating GPCRs exist, such as biased agonism[8] (also called "functional selectivity") and allosteric modulation[9]. Biased agonists are defined in opposition to balanced agonists; a biased agonist preferentially signals through one downstream pathway over another (e.g. G-protein over β-arrestin), compared to balanced agonists. In cases where signaling through one pathway leads to an unwanted side effect, biased agonists may achieve improved therapeutic efficacy. A therapeutic benefit for biased agonism has been proposed for at least thirty GPCRs[10].

Allosteric drugs are those that bind the receptor at a spatially distinct site from the orthosteric site. The high structural conservation of orthosteric sites makes it difficult to target one receptor without also engaging multiple family members. This non-specificity can result in toxicity and other side-effects that result in clinical failure[7]. In contrast, allosteric drugs targeting non-conserved sites can have high specificity and reduced toxicity. Further, since allosteric drugs modulate responses to physiological ligands, their effects temporally and spatially match physiological signaling processes[11]. Due to these major advantages, increasing effort is being put toward development of biased and allosteric modulators[12]. However, with our current understanding of GPCR function, these molecules are typically only found by blind screening or iteration upon previously discovered molecules[13,14].

In order to design better GPCR drugs, an improved understanding of the rich functional and allosteric architecture linking agonist binding with intracellular signaling is required. Much work has focused on using the structures of active and inactive states of receptors to try and understand this allosteric mechanism[15,16]. This has provided important mechanistic insights, including descriptions of conserved "microswitch" motifs, such as the CWxP, DRY, PIF, and NPxxY motifs, which are sites where local structural rearrangements occur upon GPCR activation[16]. However, these structural descriptions are only the starting point for assigning a functional role for the hundreds of individual amino acids in each highly dynamic receptor.

A powerful complementary approach for understanding how proteins work is the use of mutagenesis followed by functional characterization[17]. Serial experiments have measured the effects of hundreds of alanine substitutions on GPCR signaling through one[18] or two[19,20] pathways, revealing a rich diversity of signaling responses to mutations to only a single amino acid. Pooled approaches have been used to characterize surface expression[21–25], ligand binding[23,26], and single or few concentration transcriptional responses downstream of GPCRs[27–30]. However, the limited throughput[18–20], focus on only one of the 19 possible chemical changes at each residue[18-20], and lack of quantification of multi-modal responses and signaling parameters limit the insights that can be obtained by these approaches[21–30].

Many of the most basic questions in GPCR signaling remain unanswered. Challenges include describing and understanding how the sequence of a receptor determines multi-modal and non-linear

signaling properties and the causal mapping from sequence to biophysics to signaling characteristics. Properties to understand include receptor abundance, maximum response, basal signaling, and potency of response of a receptor to ligand binding (the amount of agonist needed to elicit a response). Mutating receptor residues to alanine has provided important insights into this, but testing only one alternative amino acid provides limited perturbation at each position. An additional challenge is to test how perturbations across a receptor's structure alter bias in the recruitment of different transducer proteins[31].

With respect to drug discovery, a key challenge is to understand how ligands modulate receptor signaling. Are all ligand-contacting sites equally important for a response or just a subset of "hotspot"[32] residues? Are all ligand contacts equally important for different functional responses to a ligand or are some contacts more important for particular responses? Where else in the receptor mediates the response to a ligand? Are residues important for the response to ligand equally important for different functional responses? Where, across the structure of a receptor can perturbations alter signaling bias?

A major goal in GPCR drug discovery is to identify allosteric sites on a receptor where perturbations can inhibit, activate, or modulate signaling. GPCRs, like all proteins, have many small pockets to potentially therapeutically target[33]. For each receptor it would be extremely useful to know at which of these sites perturbations alter multi-modal receptor responses and how, including modulation of quantitative signaling parameters and bias.

Here we present a general high-throughput method, GPCR-MAPS (GPCR multiplexed assays with pathway specificity), to rapidly generate comprehensive functional maps of GPCRs. As a first application, we apply GPCR-MAPS to the beta-2 adrenergic receptor (β2AR), one of the most extensively characterized receptors. β2AR is expressed in the cardiovascular system where it modulates smooth muscle relaxation and bronchodilation; Many β2AR agonists have been developed and they are used for treating asthma as well as chronic obstructive pulmonary disease[34].

GPCR-MAPS has four components: First, we generate a complete site-saturation library of a receptor, consisting of all possible amino acid substitutions in all positions. Second, we quantify the cell surface expression of the library of variants to quantify defects in fold stability and trafficking. Third, we use pooled experiments to quantify the recruitment of conformation-specific interaction partners to every receptor mutant in response to ligand treatment. Using ligand titrations allows the complete concentration-response activation functions of all possible receptor variants to be quantified in a single experiment. Measuring the recruitment of different interaction partners quantifies changes in receptor bias. Fourth, we integrate the resulting data to generate rich multi-modal functional maps of how the quantitative and multi-modal functions of a receptor are altered by chemically diverse perturbations throughout its entire structure.

Applied to β2AR, GPCR-MAPS quantifies the activation functions and multi-modal characteristics of >7,500 different receptors in pooled experiments. The resulting multi-modal maps reveal a modular receptor architecture, with a core activation network surrounded by residues controlling quantitative parameters and bias. The multi-modal maps also identify multiple allosteric surface pockets, including the pockets previously identified by the serendipitous discovery of β2AR allosteric ligands and a novel pocket with no known ligands. We believe application of the GPCR-MAPS approach across the GPCR superfamily will advance our understanding of the largest family of human membrane receptors and enable rational, precision drug design.

Reviewer #2 (Remarks to the Author):

Mighell and Lehner investigate how sequence variation in a G protein–coupled receptor influences function, applying deep mutational scanning (DMS) to characterize mutational effects on receptor signaling. Their premise is that a "functional map" can reveal fundamental features of GPCR function, identify potential allosteric sites for drug discovery, and ultimately guide precision GPCR therapeutics. As a model, they use the β_2-adrenergic receptor, a well-characterized GPCR with extensive prior structural and mutagenesis data. This work builds on several recent studies that have applied saturation DMS or comprehensive scanning approaches to GPCRs. The authors introduce some methodological innovations, notably a large-scale DMS at 12 agonist concentrations to assess mutational effects on dose–response relationships.

The technical execution is strong, but in several places the novelty of the conclusions is limited because many findings recapitulate known mutational effects from earlier β_2AR studies. In particular, while the authors identify some new functionally important residues, these are not validated prospectively, and several broader claims about the impact of DMS on drug discovery are not fully substantiated. Overall, I think the manuscript is likely to be of interest to NSMB readers as a great example of the power of DMS to understand membrane protein function, but could be strengthened by the points outlined below.

We thank the referee for their enthusiasm and very constructive suggestions.

Major points:
1) The authors point out that prior DMS studies are limited by their use of signaling systems because these have non-linearities and feedback that influence the data. Instead the authors set up a system to measure protein recruitment directly, using an active-state specific nanobody (Nb80) and beta-arrestin as proxies for function. The rationale for using Nb80 and β-arrestin recruitment as readouts is sound, especially in avoiding non-linearities and feedback seen in transcriptional signaling assays. However, the manuscript equates Nb80 recruitment with "G protein signaling" or "Nb80 signaling" and β-arrestin recruitment with "arrestin signaling," and interprets mutation effects as "Nb80-biased" or "arrestin-biased." This framing misleads readers, since Nb80 recruitment does not necessarily share the same allosteric requirements as G protein activation. The authors could strengthen their case by emphasizing the advantages of the recruitment assays (e.g., reduced signal amplification) rather than implying direct equivalence to canonical signaling pathways.

We appreciate this critique and totally agree. We have made updates throughout the text to clarify that we are measuring recruitment and not signaling and we have made changes to clarify that our observations only pertain to Nb80 and not necessarily G-protein.

We have updated the text:

Abstract:

> *The approach, GPCR-MAPS, quantifies direct recruitment of proteins to a receptor and deconvolves the substantial effects of mutations on receptor expression.*

Introduction, paragraph 10:

> *Third, we use pooled experiments to quantify the recruitment of conformation-specific interaction partners to every receptor mutant in response to ligand treatment. Using ligand titrations allows the complete dose-response activation functions of thousands of receptor variants to be quantified. Measuring the recruitment of different interaction partners quantifies changes in receptor bias.*

Results, "GPCR-MAPS: multiplexed assay with pathway specificity" section, first paragraph:

> *Our goal is to read out receptor states as directly as possible. In contrast to other approaches[27–30], we therefore focused on quantifying direct binding interactions with receptors.*

Results, "GPCR-MAPS: multiplexed assay with pathway specificity" section, paragraph 4:

> *Additional selections then quantify direct binding of receptor interaction partners. To quantify recruitment of receptor interaction partners in response to ligand treatment we developed a modular protein interaction assay based on a previously described platform[41] but extensively re-engineered to enable massively multiplexed experiments (see Materials and Methods).*

2) The system the authors have set up is quite complex - I think readers would benefit from understanding why such a complex system is necessary (e.g. why not just remove the whole LOV-luciferase system. It's could intuit based on our own experience in the GPCR field, but likely will not be apparent to all. I think it would also be very high yield for the authors to move the wild-type dose response curve for isoproterenol shown in Fig 5f (ideally with replicates) to the first figure to highlight the robustness of the system. Since the authors want to connect signaling to their assay, the manuscript could be improved by comparing the EC50 for iso in the transcriptional assay to a cAMP assay.

We agree that explaining why these additional components are necessary is a good idea and have changed the text accordingly:

Results, “GPCR-MAPS: multiplexed assay with pathway specificity” section, paragraph 5:

> *Cleavage therefore relies on coincidence of the NLuc:eLOV interaction as well as the GPCR:binding partner interactions, reducing background signal[41].*

We appreciate the suggestion of moving the dose response curve to figure 1, but we have decided not to implement this change as we think it fits better in figure 5, where we are introducing dose response analysis.

Also, we agree that comparing the iso EC50 in the transcriptional assay to that measured via other means is valuable, which is why we have included the comparison of our EC50 estimates with estimates from a previous alanine scanning study that used not cAMP assay but a BRET assay that monitors G-protein activation in Figure 6b.

3) The DMS identifies interesting functional residues, such as N-terminal glycosylation sites and C-terminal positions affecting signaling. These could be the most novel findings of the work. Even limited prospective validation (for example, testing a subset of mutations in a simple cAMP assay) would greatly enhance confidence that the approach can generate actionable predictions, not just retrospective rationalizations.

We appreciate that the reviewer finds the N-terminal and C-terminal functional residues interesting- indeed, we did not expect this result. As this publication is focused on the development and validation of the new GPCR-MAPS method, we made the decision to work on the well-studied b2AR so that we could do extensive retrospective validation/rationalization. Indeed, we think the fact that our data is consistent with decades of work on b2AR is good reason to believe that the approach will indeed generate actionable predictions. However, we leave it to future work by us and others in the community to explore the many implications of this dataset in more detail.

4) The functional meaning of constitutively active (COF) mutations is unclear. For example, for S120, can the authors rationalize which substitutions yield gain- vs. loss-of-function? Additional targeted assays, even for a few sites, would clarify how predictive the DMS is for COF phenotypes.

Change of function (CoF) sites are those where different mutations have highly divergent functional effects. We agree that the discussion of these sites could benefit from unpacking a bit exactly which residues are leading to which outcome, and especially if there is some physiochemical rationalization for the behavior. Therefore, in the figures highlighting CoF residues, we labeled the identities of the variants so that readers can explore the trends (Figure 2k, Figure 3d, and Figure 6h). We also added text to highlight some patterns:

Results, section “Complete functional maps of Nb80 and β-arrestin recruitment”, first paragraph:

> *An example of a strong CoF residue is L75^{2x46} (Figure 2k), which is near the allosteric sodium binding site and neighbors LoF residue A76^{2x47}. At this position, mutations to small, uncharged residues (G, S, T, A, C) decrease surface expression, but increase Nb80 and β-arrestin recruitment relative to the modeled expectation. On the other hand, positively charged residues (H, R, K) are well expressed but strongly decrease recruitment of both proteins.*

Results, section “Complete functional maps of Nb80 and β-arrestin recruitment”, first paragraph:

> *At S120^{3x39}, which neighbors I121^{3x40} of the PIF motif, positively charged substitutions (R, K, H) yield stable receptors with significantly reduced recruitment, while the E and Q also yield stable receptors but significantly increase recruitment (Figure 3d).*

Results, section “Potency tuning”, paragraph 6:

> *Changing the F to other aromatics (Y or W) at position 282 yield stable receptors with strongly reduced potency, suggesting these mutations could stabilize the inactive state of the receptor (Figure 6h). Other*

mutations change expression-adjusted potency in either direction, suggesting idiosyncratic effects on the active and/or inactive conformations.

5) For the Nb80 vs arrestin comparison - a major reason for this difference could come down to mutations that influence receptor expression levels. Did the authors account for surface expression when making this comparison? For example, it is known that W99 is an important residue for GPCR folding - is the "Nb80 bias" simply because mutations at this residue lead to less receptor expression and it is easier to see Nb80 recruitment at low expression levels compared to arrestin recruitment?

Thanks for this critique. To evaluate if expression level was having an effect on the apparent bias of mutations, we re-did the analysis while directing taking the expression level into consideration. Namely, instead of directly comparing Nb80 and β-arrestin recruitment, we first normalized by surface expression (LOWESS fit, taking residual of recruitment to expression, as in Figures 2 and 3), and then compared these two recruitment: expression residuals. We fit a LOWESS curve and calculated residuals just as in the direct comparison method, and calculated Nb80: β-arrestin residuals from this line. Finally, we directly compare the set of residuals calculated the original way (direct comparison) and the way explicitly taking expression into account, and they are very highly correlated (Pearson's r = 0.96, see figure below). Since these two methods yield such similar outcomes, we have decided to keep the analysis as originally performed.

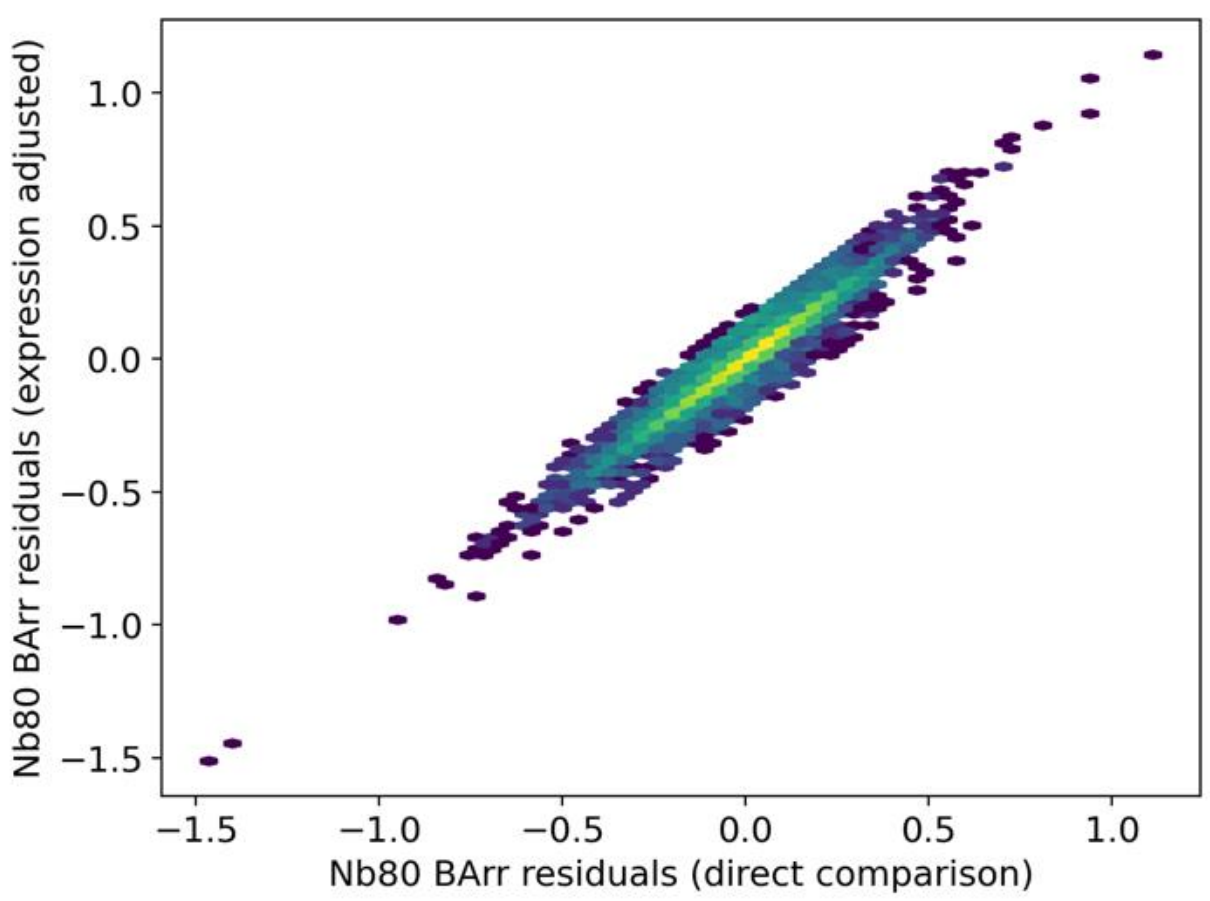

However, upon your suggestion we took a closer look at W99 and variants at this position are indeed lowly expressed, calling into question the functional relevance of bias at this position. Therefore, we have decided to remove the callout to W99 in the figure and the text:

Results, "The extracellular vestibule allosterically controls recruitment bias" section, paragraph 1, **Text removed**:

The most strongly Nb80 biased residue, W99^{23x50}, was previously identified as particularly mutationally intolerant[27]; however only by measuring recruitment of both proteins could we uncover its strongly biased effects (Figure 4f).

6) The dose response curve work is heroic and I applaud the authors for this. But at some level it's a bit less impactful if the major conclusions could have been predicted by an alanine scan (though it seems that the majority of the correlation in Fig. 6b is driven by a small subset of LOF sites). I think perhaps some of the most interesting results here are the COF sites. The authors point out F282 as an interesting COF site, which it does seem to be, e.g. even very lowly expressed mutations at this position seem to have high iso potency. This would be an example of a fascinating new observation to dig deeper - given the important structural changes known to happen at this site, can they at least rationalize with the mutations observed?

Thanks for acknowledging the scale of the concentration-response experiment! We use the previous alanine scanning data to validate the accuracy of our measurements, but we do not believe that the major conclusions could have been predicted by an alanine scan. F282 is an excellent example of this- only by measuring all amino acid changes at this site can we see that different amino acids have wildly different functional effects.

We have provided some new rationalization in the text:

Results, “Potency tuning” section, paragraph 6:

> *F282^{6x44} of the PIF motif, which undergoes structural rearrangement upon activation, is the strongest EC_{50} CoF residue (Figure 6h) consistent with previous data showing multiple constitutive activating mutations and at least one attenuating mutation[70]. Changing the F to other aromatics (Y or W) at position 282 yield stable receptors with strongly reduced potency, suggesting these mutations could stabilize the inactive state of the receptor (Figure 6h). Other mutations change expression-adjusted potency in either direction, suggesting idiosyncratic effects on the active and/or inactive conformations.*

7) Figure 7 is challenging to interpret due to color schemes and unclear selection thresholds. Showing the entire receptor surface, with consistent coloring and explicit criteria for site selection, would help assess whether identified sites are truly discrete and novel.

We agree that the figure was difficult to interpret. Therefore, we have simplified the main figure and moved heatmaps to the supplement. In the main figure, we have now labeled the different colors, with the criteria for site selection being the same as in previous sections of the paper. We have moved the heatmaps to the supplement (supplemental Figure 7).

Minor points:
1) Lines 22-24: the majority of GPCR drug discovery failures are not because molecules target the orthosteric pocket. In general, orthosteric targeting has been incredibly fruitful for GPCR drug discovery and there are many orthosteric molecules that have entered Phase2 and Phase 3 trials. The vast majority of failures are because we simply don’t understand the human biology well enough or the preclinical models were not predictive.

Thanks for this comment- we agree that our statement, as written, was not quite right. We have re-written this sentence to hopefully clarify:

Abstract:

> *Most GPCR drugs bind a conserved orthosteric pocket, which can result in non-specificity, toxicity and clinical failure*

2) Line 54 - for many GPCRs, phosphorylation is not just limited to the C tail; often can happen in other intracellular loops.

Good point! We have made the change:

Introduction, first paragraph:

> *Receptor activation also results in phosphorylation of the receptor, including in the C-terminal tail, and recruitment of β-arrestin, which terminates G-protein signaling by internalizing the receptor, and also scaffolds G-protein independent signaling[4].*

3) Figure 4e/f legend is flipped

Thanks for catching this! We have corrected this error.

4) In the volcano plots, it would be helpful to have labels for the residues that are called out in the figure - otherwise it’s a bit unclear how well these scored relative to the other sites

Great suggestion! We have added these labels to Figure 2f, 2g, 4d, and 6e.

Reviewer #3 (Remarks to the Author):

In their manuscript, Mighell and Lehner describe the generation of functional maps of GPCRs covering the complete coding sequence of a receptor. They measured surface expression of and arrestin and nanobody 80 (Nb80) interaction for a DMS library of mutants of the beta-2-adrenergic receptor. Residues were then classified as loss-of-functon(LoF), gain-of-functon(GoF), and change-of-functon(CoF) where the function differed clearly from the expected function at the given expression level. The LoF, GoF and CoF positions were then analysed in the context of GPCR structures, the LoF residues were used to define a 'core activation network'. The authors identified residues with a higher functional impact on either arrestin or Nb80 and analysed their positions in structures of the beta-2-adrenergic receptor, where they identified ECL2 as a hotspot for biased agonism. Finally, they rationalised the binding sites of allosteric modulators in context of the whole data set.

The authors have developed a new method termed GPCR-MAPS, which allows direct quantification of functional consequences of mutations in GPCRs for saturation mutagenesis libraries. This is a major advancement over the previously used transcriptional reporters far downstream of direct GPCR signalling. In addition, twelve concentrations of ligand were used, which is an improvement over the four concentrations used in previous GPCR-DMS experiments (ref 26).

We thank the referee for their enthusiasm and very constructive suggestions.

Instead of signalling, protein-protein interaction was quantified. While this certainly helps with comparisons of amplified G protein signalling pathways and non-amplified β-arrestin recruitment, it should still be made clearer that interaction rather than signalling was measured. In addition, Nb80 serves as a surrogate for G protein activation in this work. While Nb80 binding stabilises an active receptor state, it is not signalling competent. Nb80 and any G protein are very different proteins, which also differ in their binding mode and interactions, as can be seen in a comparison of the respective complex structures (3P0G and 3SN6). Nb80 binding could possibly be used as a surrogate for receptor activation potential, but not as a surrogate for G protein activation. Here, heterotrimeric G protein or, as mentioned, at least mini-Gs should be used to evaluate the functional consequences of all mutations to justify the claim that 'signaling through both canonical pathways' was tested.

Thanks for this critique, which was shared by reviewer 2. We appreciate this critique and totally agree. We have made updates throughout the text to clarify that we are measuring recruitment and not signaling and we have made changes to clarify that our observations only pertain to Nb80 and not necessarily G-protein.

We have updated the text:

Abstract:

> *The approach, GPCR-MAPS, quantifies direct recruitment of proteins to a receptor and deconvolves the substantial effects of mutations on receptor expression.*

Introduction, paragraph 10:

> *Third, we use pooled experiments to quantify the recruitment of conformation-specific interaction partners to every receptor mutant in response to ligand treatment. Using ligand titrations allows the complete dose-response activation functions of thousands of receptor variants to be quantified. Measuring the recruitment of different interaction partners quantifies changes in receptor bias.*

Results, "GPCR-MAPS: multiplexed assay with pathway specificity" section, first paragraph:

> *Our goal is to read out receptor states as directly as possible. In contrast to other approaches[27–30], we therefore focused on quantifying direct binding interactions with receptors.*

Results, "GPCR-MAPS: multiplexed assay with pathway specificity" section, paragraph 4:

> *Additional selections then quantify direct binding of receptor interaction partners. To quantify recruitment of receptor interaction partners in response to ligand treatment we developed a modular protein interaction assay based on a previously described platform[41] but extensively re-engineered to enable massively multiplexed experiments (see Materials and Methods).*

As expected, transmembrane helices were more sensitive to mutations compared to loops. Losses of function were stronger than gains of function, which also meets expectations. All ligand-binding site residues were identified as having a strong impact on function, this serves as a validation of the results. It is surprising, though, that concentration-response curves could not be fitted for the vast majority of mutations in the ligand-binding site.
Here, the authors should ideally compare their results with results from a previous DMS paper on beta-2-adrenergic receptor (ref 26) and the alanine-scan based results (ref 18). Do the results differ, and if yes, how? What is the threshold at which concentration-response curves can still be fitted?

We lay out the quality control criteria in supplementary figure 5e. Namely, for a curve to be successfully fit, we require that $R^2 > 0.9$, EC50 is within measured range (i.e. between 10pM and 10uM), and Emax <1.5x of the max measured value for that variant. We acknowledge that the curve fitting procedure was a challenge and we had to make tradeoffs. These criteria are meant to avoid very noisy parameter estimates that come from variants with reduced activation.

The mutation data within the orthosteric binding site is similar to the previous datasets. A direct comparison with b2AR DMS (ref 26) reveals strong correlation of effects of mutations at ligand binding residues (Spearman's $\rho = 0.64$ for ligand binding residues (compared with 0.44 for the whole dataset)).

For the alanine scan work (ref 18), overall the findings are similar, in that the ligand binding residues are highly sensitive to mutation. The sets of residues are slightly different in our analyses as they are using adrenaline as the agonist while we use isoprenaline. They found D113A to have no signaling, which is consistent with our finding of D113 being severely detrimental to signaling. We also find S204 to have severely detrimental effects, but they do not classify this residue as ligand binding. They also compare efficacy versus potency affecting residues which we do not do as the activation curves of the majority of mutations in the ligand binding site are not well fitted according to our quality control metrics.

Discrepancy in the number of variants quality-filtered can be attributed to different QC procedures: they used "visual inspection", which can be considered a reasonable approach for hundreds of variants but not for thousands.

It is not quite as striking (cf. line 295) that the majority of loss-of-function residues form an activation network connecting ligand- and transducer binding sites, as this was shown before, for GPCRs in general and for the beta-2-adrenergic receptor. Here, the authors could compare their network to previously determined networks, including general GPCR activation networks (Zhou et al., 10.7554/eLife.50279) and highlight the differences and similarities to previously identified networks.

Thanks for this critique. We have indeed now included comparisons between our identified network and the ones previously identified. We included new figure panels (Figure 3c-e) to first show the similarities and differences between ours and previous networks. Then, we highlight two interesting residue examples that highlight the value of our comprehensive approach- these are residues identified by the other two networks that we also identify, but as Change of function (S120) or even Gain of function (L124). In other words, these residues are indeed very important for signaling, but changes at these sites don't lead to simple loss of function. Finally, we also showed the identified networks on b2AR structures in Supplementary Figure 2e-g.

We also added text describing these new analyses/figures:

Results, "Loss-of-function residues define a core activation network" section, paragraph 3:

> *Previous work has used structural analysis to define an activation network for class A GPCRs[51] and alanine scanning mutagenesis to define an activation network for β2AR[18]. About half of the residues identified in our network are present in at least one of the others (19/39, Figure 3c). We identify more residues near the ligand binding site compared to the other networks (Supplementary Figure 2e-g). Interestingly, of the six residues shared by the other two networks that are not part of our core activation network, we identify four of these as either CoF ($S120^{3x39}$ and $F332^{8x50}$) or GoF ($L124^{3x43}$ and $D130^{3x43}$), emphasizing the value of empirically assaying all 19 substitutions per position. At $S120^{3x39}$, which neighbors $I121^{3x40}$ of the PIF motif, positively charged substitutions (R, K, H) yield stable receptors with significantly*

reduced recruitment, while the E and Q also yield stable receptors but significantly increase recruitment (Figure 3d). At L124^{3x43}, most variants decrease surface expression but increase relative recruitment, with the exception of the aromatic amino acids (W, F, and Y) and proline which recruit Nb80 less than expected given their expression level (Figure 3e).

Possibly the most novel of the findings in this manuscript is the identification of biasing residues, as this has not been reported for the beta-2-adrenergic receptor before at this scale.

Thanks for this perspective!

The known allosteric modulator binding sites were analysed in the alanine scan paper on beta-2-adrenergic receptor (ref 18). Were the findings in this study different from the findings in the previous study, and if yes, how?

Our findings around the pocket are more comprehensive and more nuanced in the sense that we can separate several different functional classifications. This is possible by way of our much larger dataset, our expression-normalization scheme, and our direct measure of recruitment bias.
For the AS408 NAM pocket, ref 18 makes two annotations: I214^{5x53} and C125^{3x44} are functionally important. We identify those residues as functionally important, but also several others, for a total of 7 annotations:

I214^{5x53} is LoF for EC_{50}
C125^{3x44} is CoF for EC_{50}
V129^{3x48} is LoF for maximum recruitment and LoF for EC_{50}
E122^{3x41} is CoF for maximum recruitment and CoF for EC_{50}
P211^{5x50} is biased toward Nb80 recruitment

Likewise, for the Cmpd-15 PAM pocket, ref 18 finds one residue as functionally important, D130^{3x49}. We also identify D130^{3x49} as functionally important as well as two other residues:

D130^{3x49} is GoF for both maximum recruitment and EC_{50}
K149^{4x41} is CoF for EC_{50}
F133^{3x52} is CoF for EC_{50}

To highlight that ref 18 has done a conceptually similar analysis, we added this text:

Results, “Annotation of surface pockets” section, paragraph 1:

Finally, similar to previous work with alanine scan data[18] we used our β2AR data to illustrate how GPCR-MAPS can be used to functionally annotate and prioritize potential pockets in receptors.

The predicted active pocket (Fig. 7) almost perfectly matches Orphan Site 5 identified in a paper by Hedderich et al. (10.1038/s41467-022-29609-6).

Well spotted! Thanks for this and we have added this information to the text.

Results, “Annotation of surface pockets” section, paragraph 3:

Indeed, this pocket overlaps with orphan site 5 of the GPCR “pocketome” dataset[33]; the pocket was proposed to be allosterically active based on a subset of mutations within the pocket affecting receptor activation, but mutation effects on receptor abundance were not ruled out.

Overall, the figures would benefit from additional explanations and clarifications for readers from the GPCR field who are not DMS experts.

We have made several changes to labeling and annotations throughout the figures, taking reviewer critiques into consideration, that we think have improved readability of the figures.

Comments:
Line 30: please be more specific about 7,500 proteins

We agree our wording was confusing, what we meant was that we assayed 7,500 variant sequences, in other words 7,500 unique proteins. But as this is confusing, we updated the text to say “7,500 unique variants”:

Abstract:

> *Applying GPCR-MAPS to the β2 adrenergic receptor generates >150,000 phenotypic measurements, including the full activation functions for all possible amino acid substitutions (>7,500 unique variants) in a single experiment.*

Line 30 and throughout the manuscript: please replace ‘dose-response curve’ with ‘concentration-response curve’

Thanks for the suggestion! We have made this change throughout the manuscript.

Lines 89-98: The authors mention that ‘these systems have only been established for G protein mediated signaling pathways’ but fail to mention that two of the three papers using alanine scanning and signalling assays have employed β-arrestin recruitment assays.

Thanks for this critique. We have re-written the description of previous studies to hopefully better communicate what has been done previously.

Introduction, paragraph 5:

> *A powerful complementary approach for understanding how proteins work is the use of mutagenesis followed by functional characterization[17]. Serial experiments have measured the effects of hundreds of alanine substitutions on GPCR signaling through one[18] or two[19,20] pathways, revealing a rich diversity of signaling responses to mutations to only a single amino acid. Pooled approaches have been used to characterize surface expression[21–25], ligand binding[23,26], and single or few concentration transcriptional responses downstream of GPCRs[27–30]. However, the limited throughput[18–20], focus on only one of the 19 possible chemical changes at each residue[18–20], and lack of quantification of multi-modal responses and signaling parameters limit the insights that can be obtained by these approaches[21–30].*

Line 98-99: The paper by Jones et al. (10.7554/eLife.54895) at least tested four concentrations of ligand against 7800 mutations of the beta2-adrenergic receptor. Is the claim ‘Finally, these multiplexed studies were not extended to formal dose-response analysis’ down to whether or not authors of previous papers have fitted curves for each mutant?

Essentially, yes, the claim is indeed down to the fact that the authors previously did not fit curves. But the point is also that curves fit to only four data points would not produce reliable estimates of the curve parameters. So our advance is fitting curves but also that we generated enough data (12 points) to produce high quality curve fits.

Lines 106-109 and 212-213: Were all variants indeed tested for G protein and arrestin pathways? Lines 212-213 suggest that instead of G protein, Nb80 was used as a surrogate. While binding of Gs and Nb80, respectively, stabilises an active state of the receptor, the binding modes and interactions in the binding site differ. If Nb80 was tested rather than mini-Gs, please adapt the text accordingly.

Thanks for this critique. We have updated this section and several places throughout the manuscript to clarify we are measuring recruitment of Nb80.

Line 119: **of all receptor variants**

Thanks! Fixed.

Line 161: ‘we found that substantially increased times were necessary’ – for what? Please clarify.

We have updated this text to clarify that increased times were necessary “to obtain detectable signal”.

Results, Development of GPCR-MAPS recruitment assays section, first paragraph:

> *While the original assay used transient transfection to introduce transgenic material and took place over the course of minutes, we found that substantially increased times were necessary to obtain detectable signal, presumably due to the reduction of copy number of the receptor and reporter constructs (24 hours, Supplementary Figure 1a).*

Line 201-203: Could you please comment on receptor variants that increase cell-surface expression compared to wild type?

There are 76 putative expression increasing variants, identified with the criteria that they have 95% confidence interval greater than 95 percent of synonymous WT variants expression level (this expression value is 1.21). We plotted the surface expression of each variant at each position below, with the putative expression increasers highlighted in red. 27 out of the 76 variants are localized within transmembrane domains (indicated as gray bands below). This proportion is not different from the amount of non-expression increasers: 2,773 variants within transmembrane domains out of a total of 6,715, p=0.345, Fisher’s exact test, suggesting expression increasers equally likely to fall within or without transmembrane domains.

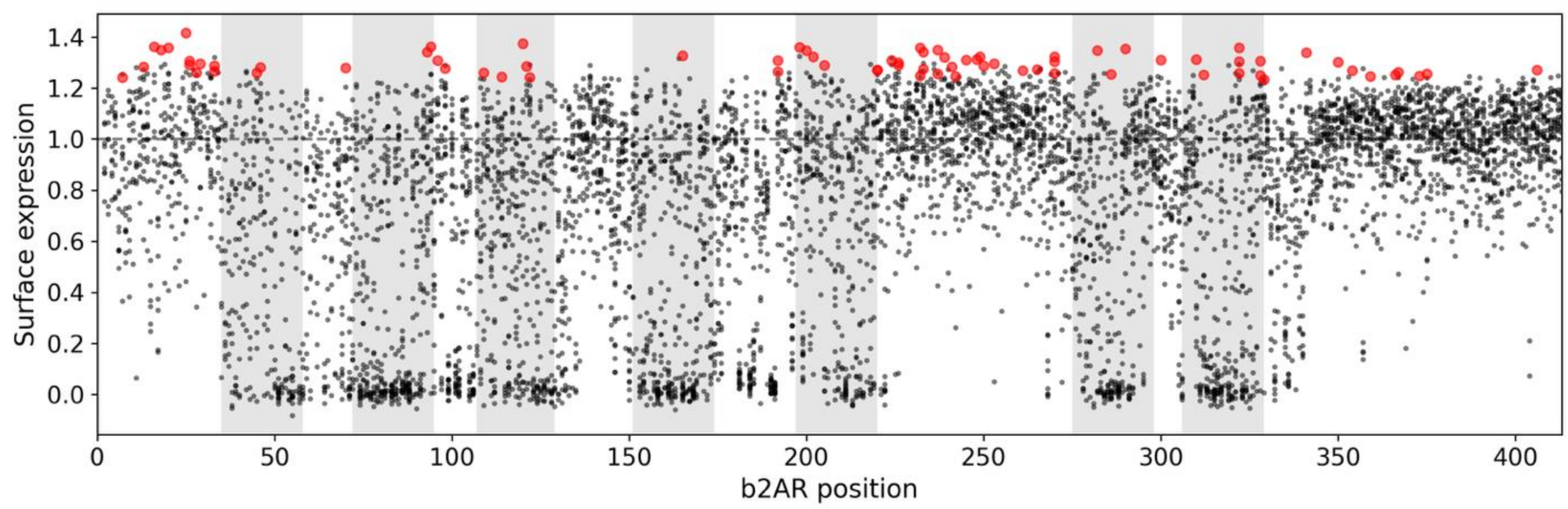

Line 227: is it possible to identify mutations with increased signalling compared to wild type?

We haven’t paid particular attention to this class of variants. Instead, we use a more global approach to find any variant that has increased activation compared to the null expectation based on expression level. In our view, in terms of understanding how the receptor functions, there is no distinction between variants that increase signaling relative to the null expectation based on expression level, regardless of the absolute level of expression.

Line 276: Please speifiy what you mean by ‘highly variable effects on signaling’? Are these positions where the effect of introducing a mutation depends on the nature of the mutation?

Thanks for pointing out this unclear language. Yes, indeed we are referring to positions where the effect of introducing a mutation depends on the nature of the mutation. We have changed the text to better communicate our meaning:

Results, “Complete functional maps of Nb80 and β-arrestin recruitment” section, paragraph 1:

> *Finally, we extended this analysis to highlight residues where mutations have highly variable effects on recruitment.*

Line 360: Could you please provide examples of concentration-response curves for at least some biased residues for readers to gauge the strength of the bias?

We have only generated full concentration-response curves for recruitment of Nb80 so we cannot make this comparison.

Line 361: please define 'bias switch'
Good suggestion, we have now defined the term:

Results, "Genetic control of receptor recruitment bias", first paragraph:

> *As before, we then compared the IQR of all remaining residues to find sites where variants have highly variable effects on bias, which we call "bias switches". To more closely match the number of biased residues, we prioritized the top 5% of residues as recruitment bias switches (Supplementary Figure 4a).*

Lines 369-371: please list the residues which make up the β-arrestin binding interface. If these are listed somewhere, the reference is missing

The residues making up the β-arrestin binding interface (from the simulation in ref. 50) are shown in Supplementary Figure 4c. We added a callout to this supplementary figure to guide the reader:

Results, "Genetic control of receptor recruitment bias" section, paragraph 2:

> *The β-arrestin binding interface is predicted to be larger, encompassing 28 residues across ICLs 1, 2, 3, and TMs 6 and 7, with 10 residues overlapping the Nb80 interface (Supplementary Figure 4c).*

Lines 444-446: While the data presented in this manuscript increases the available data >10 fold compared to alanine scans of β2AR, CB2R and AT1R, the β2AR deep mutational scanning paper by Jones et al. is not cited or taken into account in this calculation ([https://doi.org/10.7554/eLife.54895 [doi.org]](https://doi.org/10.7554/eLife.54895)). Of course, the earlier deep scanning study only tested four concentrations of ligand compared to the twelve concentrations in this study.

Thanks, we have removed this:

Results, "Activation functions for >7,500 β2AR variants" section, paragraph 2:

> *This allowed us to quantify concentration-response relationships for 7,680 different receptor variants.*

Line 454: Could you explain why the slope is 0.84, where one might expect a slope of 1?

While the theoretical slope in this case might be assumed to be 1, we used an agnostic, empirical curve fitting procedure for all variants where the best fit for the WT receptor yielded a slope of 0.84. We chose this approach (instead of constraining all curves to be fit with slope = 1) to accommodate potential differences in behavior among the thousands of variants in the library. We interpret a calculated slope of 0.84 to be quite close to 1, and we expect that if we ran the experiment several more times, the values would coalesce around 1.

Lines 455-458: Which variants were filtered and how were they treated in the analysis? These variants may be quite important

We have changed the text here to more clearly communicate how many variants were filtered and we call out Methods and Supplementary Figure 5e-f, which lay out the criteria for filtering variants. The criteria is $R^2 > 0.9$, EC_{50} within measured range (i.e. 10pM – 10uM), Emax <1.5x measured max for that variant, and slope < 2.

We also emphasize that variants with reduced activation tend to produce curves with poorer fit, meaning the filtered variants are predominantly loss-of-function, which we indeed consider to be important variants. The challenge is that curves fit to these variants tend to have noisy/misleading parameter estimates.

Results, "Activation functions for >7,500 β2AR variants", paragraph 4:

Then we fit curves to all variants; after quality control (Supplementary Figure 5e-f, Materials and Methods), this resulted in 5,588 high quality concentration-response curves with well-estimated parameters (Figure 5g-i). We note that variants with reduced activation level are more poorly fit and therefore likelier to be filtered (Supplementary Figure 5e). Our EC_{50} measurements correlate well with the results of individually assayed substitutions[18] (Pearson r=0.74, n=317 variants, Figure 6b).

Lines 465-466: please provide the correlation of Nb80 and arrestin data as a comparison.

In this case only Nb80 is compared because we only generated EC50 estimates for Nb80.

Throughout the manuscript: please use GPCRdb numbers in addition to residue numbers to facilitate comparison.

Thanks, we have done this.

Supplementary data: please add binding site annotations (for ligand, Nb80, Gs, arrestin, allosteric modulators) to the data table S1.

Great suggestion! We have done this.

Lines 489-490: How does the effect of mutations in the ligand-binding site in this work compare to the alanine scan data for the binding site?

Overall the findings are similar, in that the ligand binding residues are highly sensitive to mutation. The sets of residues are slightly different in our analyses as they are using adrenaline as the agonist while we use isoprenaline. They found D113A to have no signaling, which is consistent with our finding of D113 being severely detrimental to signaling. We also find S204 to have severely detrimental effects, but they do not classify this residue as ligand binding. They also compare efficacy versus potency affecting residues which we do not do as the activation curves of the majority of mutations in the ligand binding site are not well fitted according to our quality control metrics.

Figure 1 (and elsewhere): Nb80 does not signal, therefore I would strongly recommend replacing 'Nb80 signaling' with a more appropriate term. Please include GPCRdb numbers in detail figures (e.g. 1 j, k, l) and label the scales directly.

We have changed all relevant uses of "signaling" to recruitment. While adding the GPCRdb numbering could be useful here, due to space/font size constraints we have chosen to omit GPCRdb numbering from these figure axes. We have added direct labels to the scales.

Figure 6: a) The unit of the concentration is missing. b) Comparison of logEC50s: could the fitted line be added? When comparing logEC50s, did the authors take into account that different ligands (adrenaline and isoprenaline, respectively) were used in the two studies? In addition, please change the axis titles to 'logEC50 […]', as the displayed values do correspond to logEC50s, not EC50s. c) It is not clear whether EC50 and Emax values of Nb80 or arrestin or both are compared here. Same for d). e) would benefit from additional explanation and/or annotation.

Unit of concentration has been added to (a). The fitted line is shown in f, g, and h. The different ligands were not taken into consideration- we assume that overall effects will be similar for these two agonists, which is borne out by the data. Titles have been changed to "logEC50". We have added "Nb80" to axis labels in 6b, c, d, e, f, g, and h. We modified text to hopefully convey better the analysis taking place in Figure 6e.

Results, "Potency tuning" section, paragraph 1:

To isolate mutational effects on EC_{50} beyond those caused by changes in expression we again used LOWESS regression to model the relationship between surface expression and EC_{50} (Supplementary Figure 6a). We quantified residuals to that model to identify positions where mutations have consistently higher or lower EC_{50} than expected from their impact on expression.

Figure 7: This figure would benefit from additional annotation of the colour scales. Which mutations bias towards arrestin and Nb80? Why do the scales range from -1 to 1 rather than 0 to 1?

We have moved the relevant panels to Supplementary figures and added labels to the colour scales. The scales are -1 to 1 because the residuals can be in either direction.

Figure 8: It would be interesting to show the components of the network here, purple lines alone are not very informative. Overall, the figure should be designed in such a way that the major findings can be obtained without reading the manuscript in detail. At present, this figure does not improve my understanding of the results.

We have removed this figure.

Reviewer #3 (Remarks to the Author):

Many thanks to the authors for making substantial improvements on their manuscript.

I only have minor comments:

I previously commented that ‘7,500 proteins’ is confusing. The authors have changed this wording in the original position (line 30, first submission), but re-introduced a similar phrase in the new manuscript (line 239, ‘>7,500 different receptors’ and line 353, ‘for thousands of different receptors’). Precise wording would help the reader in all of these cases.

We have made the following changes:

Introduction, final paragraph:

> *Applied to β2AR, GPCR-MAPS quantifies the activation functions and multi-modal characteristics of >7,500 different receptor variants in pooled experiments.*

Results, “GPCR-MAPS: multiplexed assay with pathway specificity” section, last paragraph:

> *[…] ligand titrations allowing the construction of full concentration-response curves for thousands of different receptor variants in a single pooled experiment.*

Line 738 ff.: The presented dataset does not reveal the importance of the extracellular vestibule for G-protein biased activation. This would only be the case if all positions were tested in a G protein assay, not a Nb80-based assay. Please change the wording.

We have made the following change:

Results, “The extracellular vestibule allosterically controls recruitment bias” section, second paragraph:

> *The importance of the extracellular vestibule for Nb80 biased activation revealed in our mutational data is also consistent with structural data.*
